# The COVID-19 Pandemic and Associated Inequities in Acute Myocardial Infarction Treatment and Outcomes

**DOI:** 10.1001/jamanetworkopen.2023.30327

**Published:** 2023-08-25

**Authors:** Laurent G. Glance, Karen E. Joynt Maddox, Jingjing Shang, Patricia W. Stone, Stewart J. Lustik, Peter W. Knight, Andrew W. Dick

**Affiliations:** 1 Department of Anesthesiology and Perioperative Medicine, University of Rochester School of Medicine, Rochester, New York; 2 Department of Public Health Sciences, University of Rochester School of Medicine, Rochester, New York; 3 RAND Health, RAND, Boston, Massachusetts; 4 Department of Medicine, Washington University in St. Louis, St. Louis, Missouri; 5 Center for Health Economics and Policy at the Institute for Public Health, Washington University in St. Louis, St. Louis, Missouri; 6 Center for Health Policy, Columbia University School of Nursing, New York, New York; 7 Department of Surgery, Cardiac, University of Rochester School of Medicine, Rochester, New York

## Abstract

**Importance:**

The COVID-19 pandemic disrupted usual care for emergent conditions, such as acute myocardial infarction (AMI). Understanding whether Black and Hispanic individuals experiencing AMI had greater increases in poor outcomes compared with White individuals during the pandemic has important equity implications.

**Objective:**

To investigate whether the COVID-19 pandemic was associated with increased disparities in treatment and outcomes among Medicare patients hospitalized with AMI.

**Design, Setting, and Participants:**

This cross-sectional study used Medicare data for patients hospitalized with AMI between January 2016 and November 2020. Patients were categorized as Hispanic, non-Hispanic Black, and non-Hispanic White. The association between race and ethnicity and outcomes as a function of the proportion of hospitalized patients with COVID-19 was evaluated using interrupted time series. Data were analyzed from October 2022 to June 2023.

**Exposure:**

The main exposure was a hospital’s proportion of hospitalized patients with COVID-19 on a weekly basis as a proxy for care disruption during the pandemic.

**Main Outcomes and Measures:**

Revascularization, 30-day mortality, 30-day readmission, and nonhome discharges.

**Results:**

A total of 1 319 273 admissions for AMI (579 817 females [44.0%]; 122 972 Black [9.3%], 117 668 Hispanic [8.9%], and 1 078 633 White [81.8%]; mean [SD] age, 77 [8.4] years) were included. For patients with non–ST segment elevation MI (NSTEMI) overall, the adjusted odds of mortality and nonhome discharges increased by 51% (adjusted odds ratio [aOR], 1.51; 95% CI, 1.29-1.76; *P* < .001) and 32% (aOR, 1.32; 95% CI, 1.15-1.52; *P* < .001), respectively, and the odds of revascularization decreased by 27% (aOR, 0.73; 95% CI, 0.64-0.83; *P* < .001) among patients hospitalized during weeks with a high hospital COVID-19 burden (>30%) vs patients hospitalized prior to the pandemic. Black individuals with NSTEMI experienced a clinically insignificant 7% greater increase in the odds of mortality (aOR, 1.07; 95% CI, 1.00-1.15; *P* = .04) for each 10% increase in the COVID-19 hospital burden but no increases in readmissions or nonhome discharges or reductions in revascularization rates compared with White individuals. There were no differential increases in adverse outcomes among Hispanic compared with White patients with NSTEMI based on hospital COVID-19 burden. Increases in hospital COVID-19 burden were not associated with changes in outcomes or the use of revascularization in STEMI overall or by racial or ethnic group.

**Conclusions and Relevance:**

This study found that while hospital COVID-19 burden was associated with worse treatment and outcomes for NSTEMI, race and ethnicity–associated inequities did not increase significantly during the pandemic. These findings suggest the need for additional efforts to mitigate outcomes associated with the COVID-19 pandemic for patients admitted with AMI when the hospital COVID-19 burden is substantially increased.

## Key Points

**Question:**

Was the COVID-19 pandemic associated with increases in disparities in treatment and outcomes among Medicare patients hospitalized with acute myocardial infarction (AMI)?

**Findings:**

In this cross-sectional study of 1 319 273 AMI admissions, the COVID-19 pandemic was associated with significant increases in mortality and nonhome discharges and reduction in revascularization for patients admitted with non–ST segment elevation MI but not patients admitted with ST-segment elevation MI. These findings did not differ significantly by patient race or ethnicity.

**Meaning:**

This study found that while the pandemic was associated with worse treatment and outcomes in patients with AMI, race and ethnicity–associated inequities did not increase significantly.

## Teaser

This cross-sectional study investigates the association of the COVID-19 pandemic with treatment and outcome disparities among patients with acute myocardial infarction.

## Introduction

The COVID-19 pandemic has placed unprecedented stress on the US health care system.^1,2^ In addition to nearly 100 million confirmed cases of COVID-19 and more than 1 million deaths in the US,^3^ COVID-19 created disruptions and delays to usual care, particularly for urgent and emergent conditions. Prior studies^4,5,6,7^ suggested significant excess mortality from causes other than COVID-19 during the pandemic, including cardiovascular disease. Furthermore, direct and indirect outcomes associated with COVID-19 have been more pronounced among people from racially and ethnically minoritized groups. Black and Hispanic individuals experienced a 60% higher cumulative death rate from COVID-19 than White individuals,^8^ and individuals from minority groups accounted for 70% of excess deaths indirectly associated with COVID-19.^9,10^

Understanding the association of COVID-19 with health care outcomes is essential to efforts to limit the number of excess non–COVID-19 deaths, especially for members of historically marginalized groups who have been the most affected by COVID-19. It is possible that excess deaths during the pandemic were predominantly due to delays in care, with patients seeking or receiving care later in their disease course due to care availability or concerns about contracting COVID-19.^7,11,12,13,14,15^ It is also possible that patients presenting with acute illnesses did not receive usual treatments due to staff or bed shortages.^16,17^ The COVID-19 pandemic presents an opportunity to investigate whether health care systems under stress, as indicated by a high proportion of inpatients with COVID-19, provided lower-quality cardiovascular care and whether this decrease in quality of care was disproportionately large among members of racial and ethnic minority groups.

Prior investigators have shown that mortality rates after emergency surgery were higher in hospitals with a high number of COVID-19 cases,^18^ although these findings were similar across racial and ethnic groups.^18,19^ Others have previously reported that acute myocardial infarction (AMI) mortality increased during the COVID-19 pandemic.^15^ It is not known if the COVID-19 pandemic has been associated with a disproportionate increase in mortality for cardiovascular hospitalizations among racial and ethnic minority groups.

Therefore, using national Medicare data, this study aimed to answer 2 questions: Did patients hospitalized for AMI have different rates of revascularization, mortality, readmissions, or nonhome discharges in hospitals during weeks with a high COVID-19 burden compared with weeks with a low COVID-19 burden? If so, were these changes more deleterious among Black and Hispanic individuals than White individuals? The findings of this study may inform efforts by health care professionals and policymakers to create a more equitable health care system and improve the care of all patients with cardiovascular disease during the current pandemic and future pandemics.

## Methods

Columbia University approved this cross-sectional study and determined that informed consent was not required because it was exempt research. The Strengthening the Reporting of Observational Studies in Epidemiology (STROBE) reporting guideline was used to guide the reporting of this study.

### Data Source

This retrospective cross-sectional study used data from the 100% Medicare Provider Analysis and Review File and the Master Beneficiary Summary File between 2016 and 2020. These databases included beneficiary demographic information (age, sex, and self-reported race and ethnicity [Asian, Black, Hispanic, North American Native, White, and other], which is captured at the time of Social Security enrollment); *International Statistical Classification of Diseases and Related Health Problems, Tenth Revision* (*ICD-10*) diagnosis and procedure codes*; *source of admission and date of admission; admission status; urgency of admission; discharge destination, date of death; and hospital identifier. These patient-level data were merged with data from Centers for Medicare and Medicaid Services (CMS) Impact Files, which included information on hospital characteristics (geographic region, rurality, number of beds, mean daily census, disproportionate share hospital percentage, and resident-to-bed ratio).

### Study Population

We identified 1 512 924 Hispanic, non-Hispanic Black (hereafter, *Black*), and non-Hispanic White (hereafter, *White*) individuals aged 65 years and older admitted with non–ST-elevation myocardial infarction (NSTEMI) or ST-elevation myocardial elevation myocardial infarction (STEMI) between January 1, 2016, and December 31, 2020 (eFigure 1 and eTable 1 in Supplement 1). Patients were categorized as Black, non-Hispanic Black, and non-Hispanic White using the Research Triangle Institute (RTI) race code.^20^ Analyses were limited to these race and ethnicity groups owing to small sample sizes in other groups. We excluded 19 152 admissions in December 2020 to allow for assessing 30-day outcomes, 70 447 elective admissions, 102 022 readmissions occurring within 30 days of index admission, 76 transfers in from hospice, and 1954 admissions of patients to hospitals not included in CMS Impact Files. The final data set consisted of 1 319 273 observations (1 022 430 admissions with NSTEMI and 296 834 admissions with STEMI) in 3078 hospitals.

### Statistical Analysis

Our first aim was to investigate whether patients admitted with AMI had different rates of revascularization (percutaneous coronary intervention or coronary artery bypass grafting during the index admission), 30-day mortality, 30-day all-cause readmission, or nonhome discharges (death or discharge to a skilled nursing facility or nursing home, inpatient rehabilitation facility, long-term care hospital, or hospital transfer) in hospitals during times with high weekly COVID-19 burdens compared with hospitals during times with low weekly COVID-19 burdens. Consider the exemplar outcome revascularization during the index admission for STEMI admissions: We specified an interrupted time-series model in which the underlying time trend for the outcome of interest is interrupted by an exogenous shock (ie, the start of the pandemic) at a particular time. The underlying time trend represents the hypothetical scenario in which the pandemic had not occurred (ie, the counterfactual). Typically, the intervention does not vary with time, and the intervention-associated outcome can be estimated as an intercept shift from the underlying time trend. In our case, however, the intensity of the COVID-19 shock (ie, the hospital weekly COVID-19 burden) varied in intensity and over time.^21^

The model specifications are shown as follows for the baseline model (model 1):

*f*[*E*(*Y_ikt_*)] = β_0_ + β_1_*X_it_* + β_2_*RaceEthnic_it_* + β_3_Week_t_ + β_4_*COVIDmonth_t_* + β_5t_*Month_t_* + β_6_*COVID-19burden_kt_*

where *f* is the logit function, *Y_ikt_* is the outcome (revascularization) for patient *i* in hospital *k* at time *t*; *RaceEthnic_it_* is race and ethnicity (Black, Hispanic, or White), *X_it_* are patient-level covariates (age, sex, admission status [urgent or emergent], admission source [community, hospital, skilled nursing facility or nursing home, or other], dual-eligibility status, location of MI [anterior wall, lateral wall, or inferior wall], congestive heart failure [systolic, diastolic, systolic, and diastolic], right heart failure, biventricular heart failure, end-stage heart failure, complications of AMI [ventricular septal defect, left ventricle rupture, or papillary muscle rupture], prior procedure [percutaneous cardiac intervention, coronary artery bypass grafting, or heart valve surgery], dialysis, COVID-19, and individual Elixhauser comorbidities), *week_t_* is the underlying linear weekly time trend, and *Month_t_* is a set of monthly indicators to identify seasonal variation around the weekly time trend (eTable 1 in Supplement 1). *COVIDmonth_t_* is a set of monthly indicators between March and November 2020 that identify deviations from the seasonally adjusted weekly time trend during the COVID-19 period (we excluded admissions in December 2020 to observe 30-day outcomes for patients admitted in November 2020). The exposure of interest, *COVID-19burden_kt_,* is a weekly measure of the hospital proportion of Medicare patients who tested positive for COVID-19, classified as 0% to 2.0%, 2.1% to 10.0%, 10.1% to 20.0%, 20.1% to 30.0%, and greater than 30.0%. We then expanded the baseline model to include hospital characteristics (rurality, resident-to-bed ratio, disproportionate share percentage, the proportion of Black and Hispanic patients, and the volume of patients with AMI [model 2]). We specified linear variables as categorical variables because we assumed that associations between outcomes and covariates would be nonlinear except for time trends. We separately estimated models for each outcome for patients admitted with NSTEMI and STEMI.

Our second aim was to investigate whether Black and Hispanic individuals admitted with NSTEMI or STEMI experienced greater changes in revascularization rates or poor outcomes compared with White individuals in hospitals with a high weekly COVID-19 burden compared with hospitals with a low weekly COVID-19 burden. To characterize differences in outcomes in Black and Hispanic individuals compared with White individuals, we expanded the baseline model to include an interaction between the hospital weekly COVID-19 burden (*COVID-19burden_kt_)* and the indicator for race and ethnicity (model 3). For simplicity of presentation, we also estimated a simpler model in which we instead expanded the baseline model to include an interaction between a linear specification of the hospital weekly COVID-19 burden and race and ethnicity (model 4).

We treated results of unconditional analyses in which we did not adjust for any patient factors (other than age) and hospital characteristics as the main findings when we examined underlying disparities in outcomes and use of revascularization. We did this because Black and Hispanic individuals may be admitted with AMI (1) with more advanced disease and greater comorbidity burden because of the impact of social determinants of health and structural racism^22^ and (2) to lower-volume and minority-serving hospitals. Hence, adjusting for disease severity, comorbidity burden, and hospital characteristics may have led us to underestimate the magnitude of the underlying disparities by adjusting away the effects of racism before hospital admission.

We also conducted post hoc analyses that were not planned as part of our original study design and were performed to address specific questions raised during the editorial review process. In these additional analyses, we examined changes in the number and severity (STEMI vs NSTEMI) of AMI admissions before and during the pandemic and changes in mortality outcomes over time during the early pandemic. We used negative binomial regression to estimate the weekly volume of AMI admissions as a function of period (pre–COVID-19 pandemic [January 2016 to February 2020] vs COVID-19 pandemic [March 2020 to November 2020] periods) and the type of AMI (NSTEMI vs STEMI). We expanded mortality models to include an interaction term between the weekly hospital COVID-19 burden (specified as a linear term) and the period during the pandemic (March to July 2020 vs August to November 2020) to investigate whether the earlier period was associated with a greater increase in mortality (at higher weekly hospital COVID-19 burdens) compared with the later period.

All statistical analyses were performed using Stata/MP statistical software version 17.0 (StataCorp). We used cluster robust variance estimators to account for the clustering of observations within hospitals. We estimated adjusted rates and outcomes using average marginal effects. The threshold for statistical significance was a 2-sided *P* < .05. A priori, we decided not to adjust for multiple comparisons as a conservative strategy^23,24,25^ to reduce the likelihood of falsely concluding that increases in the COVID-19 burden were not associated with increases in disparities (ie, increasing the chance of a type II error). We believe this approach is justified to reduce the risk of missing a significant finding. Data were analyzed from October 2022 to June 2023.

## Results

### Patient Population

Among 1 319 273 admissions for AMI (579 817 females [44.0%]; 122 972 Black [9.3%], 117 668 Hispanic [8.9%], and 1 078 633 White [81.8%]; mean [SD] age, 77 [8.4] years), there were 1 022 439 admissions with NSTEMI and 296 834 admissions with STEMI (Table; eFigure 1 in Supplement 1). White individuals were less likely to be dually enrolled (151 183 patients [14.0%]) compared with Black (48 827 patients [39.7%]) and Hispanic (49 180 patients [41.8%]) individuals and less likely to be on dialysis (20 726 patients [1.9%]) compared with Black (10 428 patients [8.6%]) and Hispanic (7377 patients [6.3%]) individuals.

**Table.** Patient Characteristics

Characteristic	Patients, No. (%)			
	Total (N = 1 319 273)	Black (n = 122 972 [9.3%])	Hispanic (n = 117 668 [8.9%])	White (n = 1 078 633 [81.8%])
Age, mean (SD), y	77 (8.4)	75.1 (8.5)	76.1 (8.3)	77.4 (8.4)
Sex				
Male	739 456 (56.1)	56 728 (46.1)	64 964 (55.2)	617 764 (57.3)
Female	579 817 (44)	66 244 (53.9)	52 704 (44.8)	460 869 (42.7)
Admission source				
Community	1 089 005 (82.6)	108 147 (87.9)	105 742 (89.9)	875 116 (81.1)
Hospital	193 176 (14.6)	11 462 (9.3)	9428 (8)	172 286 (16)
SNF or nursing home	15 696 (1.2)	1590 (1.29)	752 (0.6)	13 354 (1.2)
Other	21 396 (1.6)	1773 (1.4)	1746 (1.5)	17 877 (1.7)
Dual eligible	249 190 (18.9)	48 827 (39.7)	49 180 (41.8)	151 183 (14)
BMI				
Underweight	21 280 (1.6)	3150 (2.6)	1329 (1.1)	16 801 (1.6)
Morbid obesity	48 758 (3.7)	5700 (4.6)	2978 (2.5)	40 080 (3.7)
Myocardial infarction location				
Anterior wall	98 222 (7.5)	7182 (5.8)	7869 (6.7)	83 171 (7.7)
Inferior wall	137 151 (10.4)	8975 (7.3)	9928 (8.4)	118 248 (11)
Lateral wall	21 867 (1.7)	1501 (1.2)	1767 (1.5)	18 599 (1.7)
Unspecified	39 594 (3)	3742 (3)	4178 (3.6)	31 674 (2.9)
NSTEMI	1 022 439 (77.5)	101 572 (82.6)	93 926 (79.8)	826 941 (76.7)
Congestive heart failure				
Systolic	238 297 (18.1)	24 494 (19.9)	21 826 (18.6)	191 977 (17.8)
Diastolic	143 395 (10.9)	16 755 (13.6)	11 356 (9.7)	115 284 (10.7)
Systolic and diastolic	82 691 (6.3)	9414 (7.7)	7137 (6.1)	66 140 (6.1)
Unspecified	72 902 (5.5)	8325 (6.8)	8837 (7.5)	55 740 (5.2)
Right heart failure	1776 (0.1)	215 (0.2)	138 (0.1)	1423 (0.1)
Biventricular heart failure	1997 (0.2)	228 (0.2)	141 (0.1)	1628 (0.2)
End-stage heart failure	1175 (0.1)	163 (0.1)	170 (0.1)	842 (0.1)
Complications of AMI				
Ventricular septal defect	665 (0.1)	15 (0)	35 (0)	615 (0.1)
LV rupture	418 (0)	7 (0.01)	28 (0)	383 (0)
Papillary muscle rupture	237 (0)	10 (0.01)	9 (0.01)	218 (0)
Prior procedures				
PCI	249 477 (18.9)	22 186 (18)	18 984 (16.1)	208 307 (19.3)
CABG	176 877 (13.4)	12 925 (10.5)	15 152 (12.9)	148 800 (13.8)
Heart valve surgery	27 562 (2.1)	1662 (1.4)	1860 (1.6)	24 040 (2.2)
Dialysis	38 531 (2.9)	10 428 (8.5)	7377 (6.3)	20 726 (1.9)
AICD	30 373 (2.3)	4225 (3.4)	2883 (2.5)	23 265 (2.2)
COVID-19	1514 (0.1)	229 (0.2)	288 (0.2)	997 (0.1)
Functional status				
Using wheelchair	5858 (0.4)	869 (0.7)	500 (0.4)	4489 (0.4)
Supplemental oxygen	40 072 (3)	3213 (2.6)	2003 (1.7)	34 856 (3.2)
Dependent on caregiver	5914 (0.5)	1145 (0.9)	1282 (1.1)	3487 (0.3)
Elixhauser comorbidity				
Cardiac arrhythmia	453 158 (34.4)	36 613 (29.8)	31 343 (26.6)	385 202 (35.7)
Valvular heart disease	236 696 (17.9)	20 220 (16.4)	17 030 (14.5)	199 446 (18.5)
Pulmonary circulation	98 265 (7.5)	12 516 (10.2)	7417 (6.3)	78 332 (7.3)
Peripheral vascular disorder	166 890 (12.7)	15 525 (12.6)	12 904 (11)	138 461 (12.8)
Hypertension, uncomplicated	529 186 (40.1)	41 175 (33.5)	46 161 (39.2)	441 850 (41)
Hypertension, complicated	619 100 (46.9)	74 646 (60.7)	59 886 (50.9)	484 568 (44.9)
Paralysis	5515 (0.4)	765 (0.6)	551 (0.5)	4199 (0.4)
Neurologic disorder, other	97 497 (7.4)	11 490 (9.3)	7949 (6.8)	78 058 (7.2)
Chronic pulmonary disease	316 864 (24)	29 969 (24.4)	21 054 (17.9)	265 841 (24.7)
Diabetes, uncomplicated	223 197 (16.9)	23 692 (19.3)	26 582 (22.6)	172 923 (16)
Diabetes, complicated	339 662 (25.8)	43 486 (35.4)	43 226 (36.7)	252 950 (23.5)
Hypothyroidism	228 080 (17.3)	11 662 (9.5)	18 731 (15.9)	197 687 (18.3)
Kidney failure	408 284 (31)	53 606 (43.6)	39 344 (33.4)	315 334 (29.2)
Liver disease	34 009 (2.6)	3914 (3.2)	3786 (3.2)	26 309 (2.4)
Peptic ulcer disease	8013 (0.6)	803 (0.7)	708 (0.6)	6502 (0.6)
AIDS or HIV	911 (0.1)	320 (0.3)	136 (0.1)	455 (0)
Lymphoma	9016 (0.7)	1037 (0.8)	636 (0.5)	7343 (0.7)
Metastatic cancer	15 993 (1.2)	1658 (1.4)	1078 (0.9)	13 257 (1.2)
Solid tumor	40 240 (3.1)	4179 (3.4)	2826 (2.4)	33 235 (3.1)
Rheumatoid arthritis	44 128 (3.3)	4082 (3.3)	2915 (2.5)	37 131 (3.4)
Coagulopathy	63 707 (4.8)	6597 (5.4)	6048 (5.1)	51 062 (4.7)
Weight loss	51 439 (3.9)	7430 (6)	4301 (3.7)	39 708 (3.7)
Fluid and electrolyte disorder	293 641 (22.3)	34 379 (28)	27 745 (23.6)	231 517 (21.5)
Anemia, blood loss	8352 (0.6)	926 (0.8)	655 (0.6)	6771 (0.6)
Anemia, deficiency	49 036 (3.7)	6596 (5.4)	4172 (3.6)	38 268 (3.6)
Alcohol abuse	25 806 (2)	3050 (2.5)	2233 (1.9)	20 523 (1.9)
Drug abuse	16 232 (1.2)	3644 (3)	1260 (1.1)	11 328 (1.1)
Psychoses	6549 (0.5)	1356 (1.1)	743 (0.6)	4450 (0.4)
Depression	127 105 (9.6)	12 516 (6.2)	7417 (7.3)	78 332 (10.3)
Total beds, No.				
<50	14 513 (1.1)	971 (0.8)	824 (0.7)	12 718 (1.2)
51-149	195 615 (14.8)	11 317 (9.2)	13 894 (11.8)	170 404 (15.8)
150-249	305 966 (23.2)	23 592 (19.2)	29 358 (25)	253 016 (23.5)
250-499	520 901 (39.5)	51 227 (41.7)	47 975 (40.8)	421 699 (39.1)
≥500	282 278 (21.4)	35 865 (29.2)	25 617 (21.8)	220 796 (20.5)
Resident-to-bed ratio				
0	553 595 (42)	41 550 (33.8)	50 711 (43.1)	461 334 (42.8)
>0-0.10	371 616 (28.2)	28 270 (23)	32 262 (27.4)	311 084 (28.8)
0.11-0.20	148 004 (11.2)	13 514 (11)	12 384 (10.5)	122 106 (11.3)
0.21-0.40	133 596 (10.1)	17 633 (14.3)	10 120 (8.6)	105 843 (9.8)
0.41-	112 462 (8.5)	22 005 (17.9)	12 191 (10.4)	78 266 (7.3)
Rurality				
Rural hospital	102 823 (7.8)	6420 (5.2)	1860 (1.6)	94 543 (8.8)
Large urban hospital	601 612 (45.6)	74 083 (60.2)	71 082 (60.4)	456 447 (42.3)
Other urban hospital	614 838 (46.6)	42 469 (34.5)	44 726 (38)	527 643 (48.9)
Disproportionate share (DSH), %				
0-9.9	68 579 (5.2)	2632 (2.1)	5054 (4.3)	60 893 (5.7)
10.0-24.9	462 132 (35)	28 751 (23.4)	26 099 (22.2)	407 282 (37.8)
25.0-49.9	695 827 (52.7)	71 719 (58.3)	56 203 (47.8)	567 905 (52.7)
≥50.0	92 735 (7)	19 870 (16.2)	30 312 (25.8)	42 553 (4)
Patients with AMI who are members of minority populations, %				
<5	370 849 (28.1)	4979 (4.1)	4043 (3.4)	361 827 (33.5)
5.0-9.9	258 352 (19.6)	10 193 (8.3)	8065 (6.9)	240 094 (22.3)
10.0-24.9	371 375 (28.2)	38 705 (31.5)	21 502 (18.3)	311 168 (28.9)
25.0-49.9	215 721 (16.4)	43 061 (35)	32 267 (27.4)	140 393 (13)
≥50.0	102 976 (7.8)	26 034 (21.2)	51 791 (44)	25 151 (2.3)
Volume of AMI hospitalizations, No.				
<200	117 722 (8.9)	13 051 (10.6)	12 828 (10.9)	91 843 (8.5)
200-499	202 031 (15.3)	22 773 (18.5)	23 266 (19.8)	155 992 (14.5)
500-999	456 162 (34.6)	45 763 (37.2)	39 778 (33.8)	370 621 (34.4)
1000-1999	427 403 (32.4)	32 790 (26.7)	31 125 (26.5)	363 488 (33.7)
≥2000	115 955 (8.8)	8595 (7)	10 671 (9.1)	96 689 (9)
Hospital weekly proportion of COVID-19 cases, %^a^				
0-2.0	91 121 (54.2)	7317 (47.5)	8579 (53.9)	75 225 (55)
2.1-9.9	52 523 (31.2)	5347 (34.7)	4729 (29.7)	42 447 (31)
10.1-20.0	16 657 (9.9)	1919 (12.5)	1669 (10.5)	13 069 (9.6)
20.1-30.0	5208 (3.1)	538 (3.5)	506 (3.2)	4164 (3)
≥30.1%	2600 (1.6)	270 (1.8)	437 (2.7)	1893 (1.4)
Outcome				
30-d Mortality	148 659 (11.3)	12 874 (10.5)	13 559 (11.5)	122 226 (11.3)
30-d Readmission	273 981 (20.8)	28 715 (23.4)	26 772 (22.8)	218 494 (20.3)
Nonhome discharge	299 145 (22.7)	28 738 (23.4)	22 780 (0)	247 627 (23)
Revascularization	675 290 (51.2)	50 509 (41.1)	51 926 (44.1)	572 855 (53.1)

Abbreviations: AICD, automatic implantable cardioverter defibrillator; AMI, acute myocardial infarction; BMI, body mass index (calculated as weight in kilograms divided by height in meters squared); CABG, coronary artery bypass graft surgery; DSH, disproportionate share hospital; LV, left ventricle; NSTEMI, non–ST segment elevation myocardial infarction; PCI, percutaneous coronary intervention; SNF, skilled nursing facility.

^a^ Excludes cases before the COVID-19 pandemic.

Black (19 870 patients [16.2%]) and Hispanic (30 312 patients [25.8%]) individuals were more likely to be hospitalized in hospitals with a very high disproportionate share percentage (≥50%) compared with White individuals (42 553 patients [4.0%]). Black (51 791 patients [44.0%]) and Hispanic (26 034 patients [21.2%]) individuals were more likely to be hospitalized in hospitals with a high proportion of Black and Hispanic individuals (≥50%) compared with White individuals (25 151 patients [2.3%]). White individuals (94 543 patients [8.8%]) were more likely to be hospitalized in rural hospitals compared with Black (6420 patients [5.2%]) and Hispanic (1860 patients [1.6%]) individuals. Hospital characteristics are shown in eTable 2 in Supplement 1.

### Association of Hospital COVID-19 Burden With Changes in Revascularization

**Figure 1.**
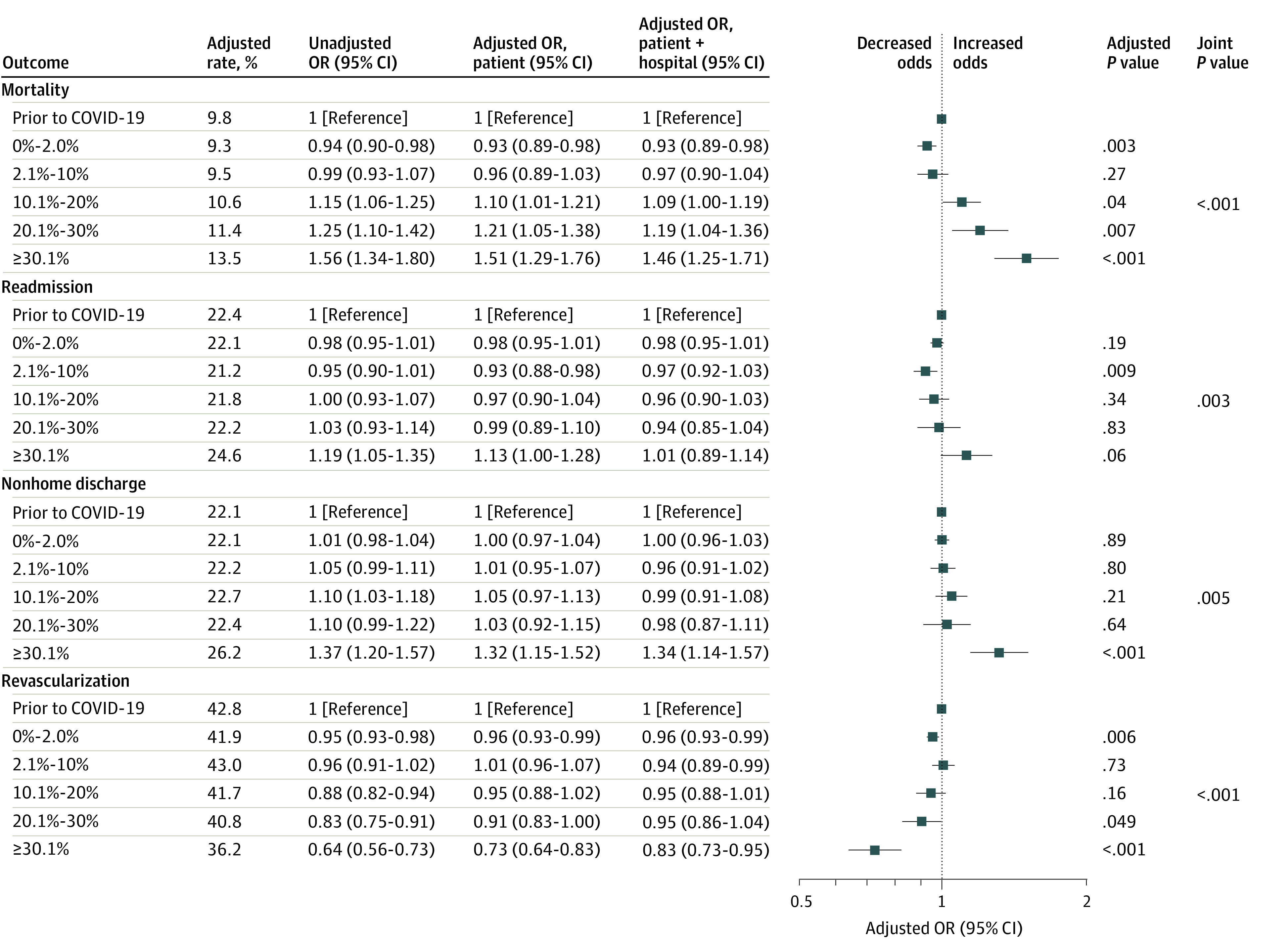
Changes in Outcomes and Revascularization by Hospital COVID-19 Burden for Non–ST Segment Elevation Myocardial Infarction The patient model was adjusted for patient age, race and ethnicity, and risk, and the patient + hospital model was adjusted for patient age, race and ethnicity, and risk and hospital characteristics. The unadjusted model was adjusted for patient age and race and ethnicity. *P* values and adjusted rates are based on the patient model, which is plotted. OR indicates odds ratio.

**Figure 2.**
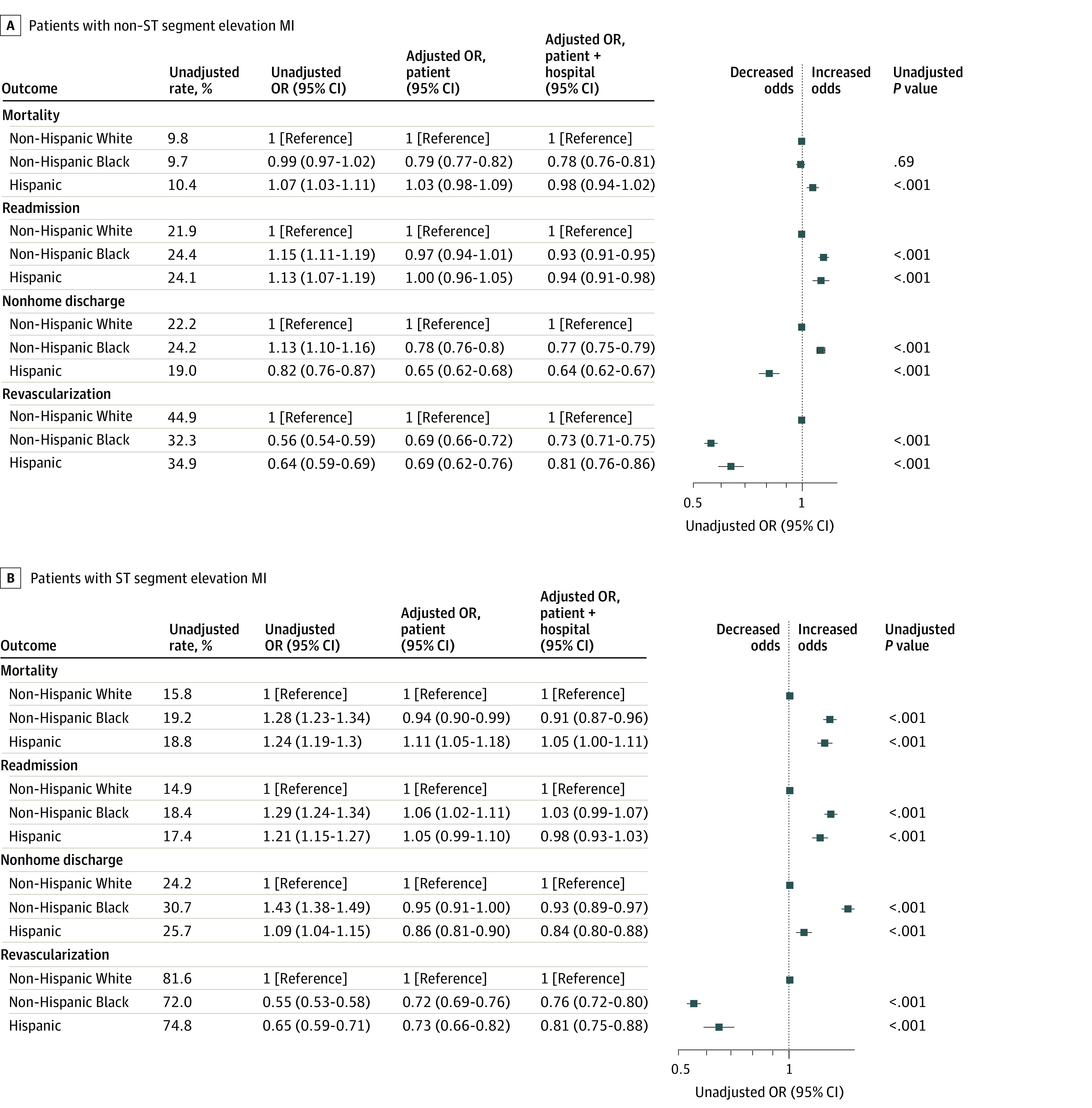
Association of Race and Ethnicity With Outcomes and Revascularization The patient model was adjusted for patient age, race and ethnicity, and risk, and the patient + hospital model was adjusted for patient age, race and ethnicity, and risk and hospital characteristics. The unadjusted model was adjusted for patient age and race and ethnicity. *P* values and unadjusted rates are based on the unadjusted model, which is plotted. OR indicates odds ratio.

**Figure 3.**
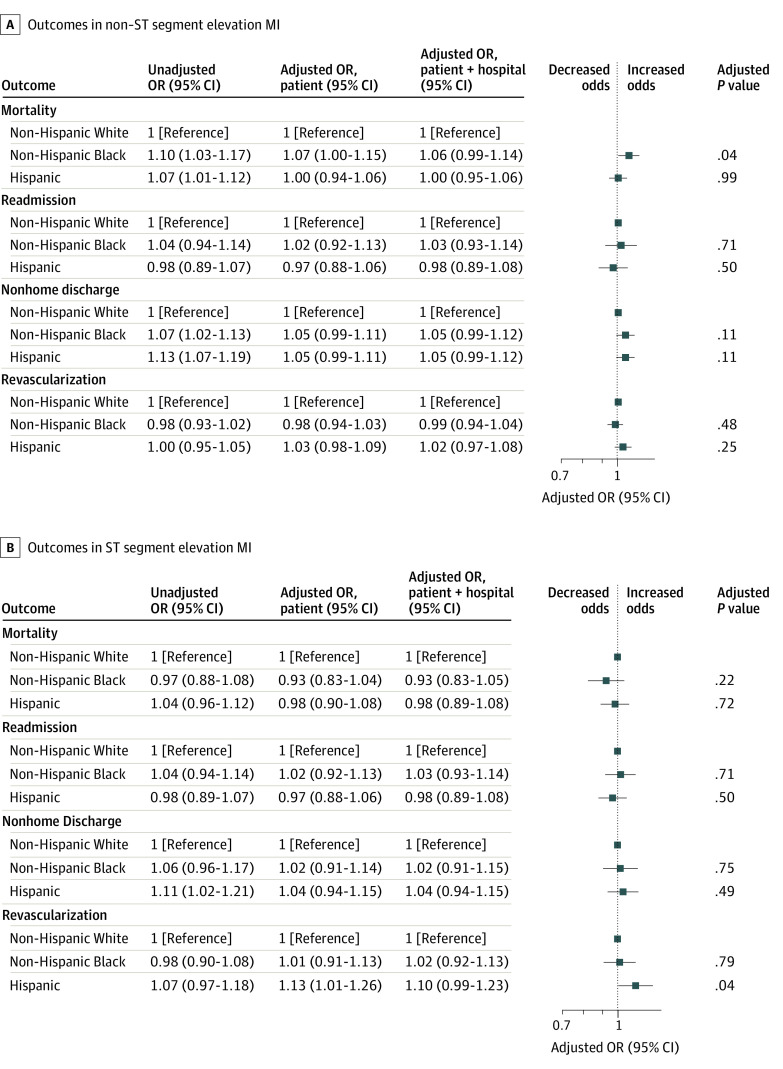
Changes in Outcomes and Revascularization Rates as a Function of Hospital COVID-19 Burden by Race and Ethnicity The patient model was adjusted for patient age, race and ethnicity, and risk, and the patient + hospital model was adjusted for patient age, race, ethnicity, and risk and hospital characteristics. The unadjusted model was adjusted for patient age and race and ethnicity. *P* values and adjusted rates are based on the patient model, which is plotted.

After adjusting for patient risk, the odds of revascularization among patients with NSTEMI overall decreased by 9% (adjusted odds ratio [aOR], 0.91; 95% CI, 0.83-1.00; *P* = .049) and 27% (aOR, 0.73; 95% CI, 0.64-0.83; *P* < .001) in patients hospitalized during weeks with a hospital COVID-19 burden of 20.1% to 30.0% and greater than 30.0%, respectively (Figure 1). Black individuals with NSTEMI were less likely to be revascularized (OR, 0.56; 95% CI, 0.54-0.59; *P* < .001) compared with White individuals, as were Hispanic patients (OR, 0.64; 95% CI, 0.59-0.69; *P* < .001) (Figure 2A). There was no differential change in revascularization during times of high COVID-19 burden for Black patients (aOR, 0.98; 95% CI, 0.94-1.03; *P* = .48) or Hispanic patients (aOR, 1.03; 95% CI, 0.98-1.09; *P* = .25) (Figure 3A).

Among patients with STEMI, Black patients (OR, 0.55; 95% CI, 0.53-0.58; *P* < .001) and Hispanic patients (OR, 0.65; 95% CI, 0.59-0.71; *P* < .001) were less likely to be revascularized compared with White patients (Figure 2B; eTable 6 in Supplement 1). However, there were no differences in the change in the use of revascularization based on hospital COVID-19 burden overall or by race or ethnicity (eFigure 2 in Supplement 1; Figure 3A and eTables 6-10 in Supplement 1).

### Association of Hospital COVID-19 Burden With Changes in Clinical Outcomes for NSTEMI

Among patients with NSTEMI overall, mortality, readmissions, and nonhome discharges increased more during weeks with high hospital COVID-19 burdens compared with hospitals before the pandemic (Figure 1; eTables 3-5 in Supplement 1). The adjusted odds of mortality increased by 10% (aOR, 1.10; 95% CI, 1.01-1.21; *P* = .04), 21% (aOR, 1.21; 95% CI, 1.05-1.38; *P* = .007), and 51% (aOR, 1.51; 95% CI, 1.29-1.76; *P* < .001) in patients hospitalized during weeks with COVID-19 burdens of 10.1% to 20.0%, 20.1% to 30.0%, and greater than 30.0%, respectively. The odds of readmission did not increase significantly (aOR, 1.13; 95% CI, 1.00-1.28; *P* = .06) in patients hospitalized during weeks with a COVID-19 burden of greater than 30% (Figure 1; eTable 4 in Supplement 1). The odds of nonhome discharge increased by 32% (aOR, 1.32; 95% CI, 1.15-1.52; *P* < .001) in patients hospitalized during weeks with a COVID-19 burden greater than 30%.

Black individuals hospitalized with NSTEMI were more likely to be readmitted (OR, 1.15; 95% CI, 1.11-1.19; *P* < .001) and discharged to a nonhome setting (OR, 1.13; 95% CI, 1.10-1.16; *P* < .001) compared with White individuals (Figure 2A; eTables 3-6 in Supplement 1). Hispanic individuals hospitalized with NSTEMI were more likely to die within 30 days of admission (OR, 1.07; 95% CI, 1.03-1.11; *P* < .001) or be readmitted (OR, 1.13; 95% CI, 1.07-1.19; *P* > .001) and less likely to be discharged to a nonhome setting (OR, 0.82; 95% CI, 0.76-0.87; *P* < .001) compared with White individuals (Figure 2A; eTables 3-5 in Supplement 1).

Black patients with NSTEMI experienced a 7% greater increase in the odds of mortality (aOR, 1.07; 95% CI, 1.00-1.15; *P* = .04) compared with White individuals for each 10% increase in the hospital COVID-19 burden (eg, when the hospital weekly COVID-19 burden increased from 10% to 20%) (Figure 3A). The adjusted mortality rates for Black individuals increased by 0.7 percentage points more than for White individuals during weeks with a 40% vs 30% COVID-19 burden. Adjusted NSTEMI mortality rates for Black and White individuals hospitalized when the COVID-19 burden was 30% were 12.8% (95% CI, 10.9%-14.7%) and 13.1% (95% CI, 12.1-14.2%), respectively; these rates increased to 14.8% (95% CI, 12.0%-17.6%) and 14.4% (95% CI: 12.9%-15.9%), respectively, during weeks with a burden of 40 %. Black patients hospitalized with NSTEMI did not experience greater increases in readmissions (aOR, 1.02; 95% CI, 0.92-1.13; *P* = .71) or nonhome discharges (aOR, 1.05; 95% CI, 0.99-1.11; *P* = .11) in hospitals during weeks with high COVID-19 burdens compared with White individuals (Figure 3A). Hispanic individuals hospitalized with NSTEMI did not experience greater increases in mortality (aOR, 1.00; 95% CI, 0.94-1.06; *P* = .99), readmissions (aOR, 0.97; 95% CI, 0.88-1.06; *P* = .50), or nonhome discharges (aOR, 1.05; 95% CI, 0.99-1.11; *P* = .11) during weeks with high hospital COVID-19 burdens compared with White individuals (Figure 3A). Results of the nonparametric model (which were consistent with the results of the linear model) are shown in eTables 7, 8, and 9 in Supplement 1.

### Association of Hospital COVID-19 Burden With Changes in Clinical Outcomes for STEMI

Among patients with STEMI, rates of readmissions and nonhome discharges were not significantly higher in hospitals during weeks with a high hospital COVID-19 burden (eFigure 2 and eTables 3-5 in Supplement 1). Odds of mortality did not increase significantly in patients hospitalized during weeks with a hospital COVID-19 burden greater than 30% (aOR, 1.28; 95% CI, 1.00-1.64; *P* = .05) (eFigure 2 and eTable 3 in Supplement 1).

Black individuals hospitalized with STEMI were more likely to die within 30 days of admission (OR, 1.28; 95% CI, 1.23-1.34; *P* < .001), be readmitted (OR,1.29; 95% CI, 1.24-1.34; *P* < .001), and be discharged to a nonhome setting (OR, 1.43; 95% CI, 1.38-1.49; *P* < .001) compared with White individuals (Figure 2B; eTables 3-5 in Supplement 1). Similarly, Hispanic individuals hospitalized with STEMI were more likely to die within 30 days of admission (OR, 1.24; 95% CI, 1.19-1.30; *P* < .001), be readmitted (OR, 1.21; 95% CI, 1.15-1.27; *P* < .001), or be discharged to a nonhome setting (OR, 1.09; 95% CI, 1.04-1.15; *P* < .001) compared with White individuals (Figure 2B; eTables 3-5 in Supplement 1). However, Black and Hispanic individuals hospitalized with STEMI did not experience greater increases in mortality, readmissions, or nonhome discharges in hospitals during weeks with high hospital COVID-19 burdens compared with White individuals (Figure 3B; eTables 7-9 in Supplement 1).

### Results of Post Hoc Analyses

**Figure 4.**
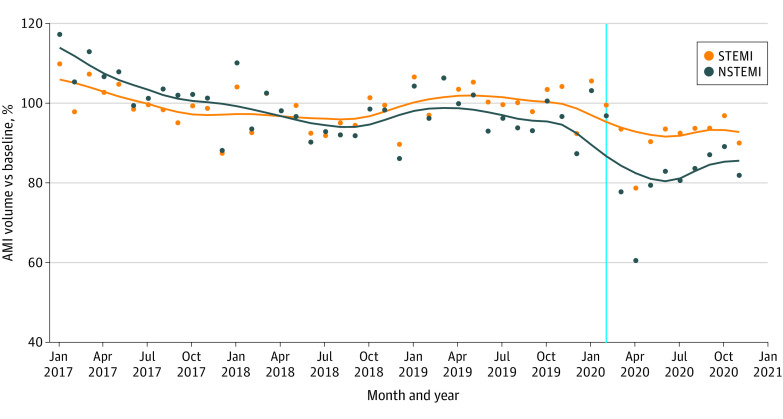
Weekly Acute Myocardial Infarction (AMI) Admissions Values are normalized to prepandemic volumes. NSTEMI indicates non–ST segment elevation myocardial infarction; STEMI, ST segment elevation myocardial infarction. Points indicate actual values. Lines represent locally weighted scatterplot smoothing (locally weighted regression) lines.

Compared with the prepandemic period, the weekly AMI admission rate decreased 5.2% during the pandemic (incident rate ratio [IRR], 0.95; 95% CI, 0.90-0.997; *P* = .04) (Figure 4). The overall IRR of STEMI to NSTEMI was 0.29 (95% CI, 0.28-0.29; *P* < .001), while the ratio of NSTEMI to STEMI decreased by 12.1% (IRR, 0.88; 95% CI, 0.83-0.93; *P* < .001) during the pandemic compared with before the pandemic. We did not find evidence that the 30-day mortality rate increased significantly between March to July 2020 vs August to November 2020 (reference period) for STEMI (aOR, 0.95; 95% CI, 0.86-1.04; *P* = .26) or NSTEMI (aOR, 1.05; 95% CI, 0.98-1.13; *P* = .16).

## Discussion

In this cross-sectional study of 1 319 924 hospitalizations for AMI, patients admitted with NSTEMI to hospitals during weeks with a high hospital COVID-19 burden were less likely to undergo revascularization and more likely to die within 30 days of admission and be discharged to a nonhome setting compared with patients admitted to hospitals during weeks with a low hospital COVID-19 burden. The early phase of the pandemic was associated with a 5.2% reduction in weekly AMI admissions, with greater reductions in NSTEMI admissions compared with STEMI admissions. There was no substantial evidence of differential COVID-19 spillover among Black or Hispanic individuals hospitalized with NSTEMI or STEMI in mortality, revascularization, readmissions, or nonhome discharges.

The finding that a high hospital COVID-19 burden was associated with worse outcomes in patients with AMI is consistent with previous research showing that the pandemic was associated with a higher risk of mortality in adult patients undergoing major surgery in the US^18^ and that AMI mortality increased during COVID-19 in a 12-hospital system.^15^ The reduction in the revascularization rate for NSTEMI during the pandemic is also consistent with the decrease in urgent and emergent surgeries reported previously.^19^ The lack of decrease in revascularization for STEMI may reflect the less discretionary nature of percutaneous coronary intervention in this clinical setting. Long-term clinical consequences, particularly for the development of heart failure and other sequelae of AMI, should be monitored closely at the population level in coming years. The lack of an increase in disparities in revascularization rates for AMI during the pandemic is consistent with previous research^19^ showing that Black and White individuals had similar reductions in elective cardiac surgery during the early phase of the pandemic.

Importantly, although the disparity in revascularization rates did not worsen during the pandemic in hospitals with high COVID-19 burdens, the existing gap in revascularization rates was nonetheless striking. Black and Hispanic individuals hospitalized after AMI had 35% to 45% lower odds of undergoing revascularization after AMI compared with White individuals. Given the proven, substantial clinical benefit of this treatment, the persistence of disparities in revascularization remains a significant cause of concern 3 decades after these disparities were first described.^26,27^ It is unlikely that patient preferences alone can explain these disparities given that similar disparities have been observed in the allocation of heart transplants and ventricular assist devices after accounting for patient preferences.^28^ These inequities likely reflect a combination of structural racism, which can impact access to high-quality care based on systematic historical divestment in predominantly Black communities, and individual or interpersonal racism, which can influence a clinician’s likelihood of referring patients for appropriate procedures.^29,30^

### Limitations

Our study has several potential limitations. First, in reporting our results on baseline disparities, we chose to present the results of our unadjusted analyses as the primary findings because Black individuals may present with more cardiovascular comorbidities compared with White individuals.^31^ As a result, adjusting for differences in baseline health may reduce the magnitude of the disparities between Black and White individuals. Second, our analysis is based on Medicare patients aged 65 years and older, and results may not be applicable to younger populations. In particular, many patients younger than age 65 years, especially if they are Black or Hispanic, lack health insurance or have inadequate coverage.^32^ In addition, our study may not include all Medicare Advantage patients, who are more likely to be Black or Hispanic.^33^ Medicare Advantage plans may attract more individuals with low incomes owing to the availability of plans with no premiums,^33^ and these patients may have been disproportionately affected by the pandemic compared with those in traditional Medicare. Third, our measure of hospital COVID-19 burden was based on Medicare patients and did not include all adult patients. Fourth, our analyses were conditional on hospital admission and did not account for disparities in AMI prevalence or death before hospital admission. Fifth, because this is a nonrandomized study and unmeasured confounding is likely, our findings cannot be used to make causal inferences.

## Conclusions

In this cross-sectional study of more than 1.3 million AMIs, patients hospitalized in hospitals during weeks with large numbers of patients with COVID-19 had a lower likelihood of undergoing revascularization and a higher likelihood of death within 30 days, readmission, and discharge to a nonhome setting. Race and ethnicity–associated inequities did not increase significantly during the pandemic. These findings suggest that policy and clinical interventions are needed to ensure that hospitals can continue to provide high-quality, evidence-based care for all patients, even in times of strain or stress.

## Notes

Supplement 1. **eFigure 1.** Flow Diagram Describing Selection of Admissions in Analytic Cohort **eFigure 2.** Changes in Outcomes in Patients With Non–ST Segment Elevation Myocardial Infarction by Hospital COVID-19 Burden **eTable 1.** Codes Used to Identify Patient Cohorts, Comorbidities, and COVID-19 **eTable 2.** Hospital Characteristics **eTable 3.** Mortality at 30 d **eTable 4.** Readmission at 30 d **eTable 5. **Nonhome Discharges **eTable 6. **Revascularization **eTable 7.** Mortality at 30 d With Interaction of Race and Ethnicity and Hospital COVID-19 Burden **eTable 8.** Readmission at 30 d With Interaction of Race and Ethnicity and Hospital COVID-19 Burden **eTable 9. **Nonhome Discharges With Interaction of Race and Ethnicity and Hospital COVID-19 Burden **eTable 10. **Revascularization With Interaction of Race and Ethnicity and Hospital COVID-19 Burden

Supplement 2.
Data Sharing Statement
