# Supplementary material for: The COVID-19 Pandemic and Associated Inequities in Acute Myocardial Infarction Treatment and Outcomes
Source: JAMA Netw Open. 2023 Aug 25;6(8):e2330327. doi: 10.1001/jamanetworkopen.2023.30327 (PMC10457721; doi:10.1001/jamanetworkopen.2023.30327)
Supplement: Supplement 1. — eFigure 1. Flow Diagram Describing Selection of Admissions in Analytic Cohort eFigure 2. Changes in Outcomes in Patients With Non–ST Segment Elevation Myocardial Infarction by Hospital COVID-19 Burden eTable 1. Codes Used to Identify Patient Cohorts, Comorbidities, and COVID-19 eTable 2. Hospital Characteristics eTable 3. Mortality at 30 d eTable 4. Readmission at 30 d eTable 5. Nonhome Discharges eTable 6. Revascularization eTable 7. Mortality at 30 d With Interaction of Race and Ethnicity and Hospital COVID-19 Burden eTable 8. Readmission at 30 d With Interaction of Race and Ethnicity and Hospital COVID-19 Burden eTable 9. Nonhome Discharges With Interaction of Race and Ethnicity and Hospital COVID-19 Burden eTable 10. Revascularization With Interaction of Race and Ethnicity and Hospital COVID-19 Burden [file jamanetwopen-e2330327-s001.pdf]

Supplemental Online Content

Glance LG, Joynt Maddox KE, Shang J, et al. The COVID-19 pandemic and associated inequities in acute myocardial infarction treatment and outcomes. *JAMA Netw Open*. 2023;6(8):e2330327. doi:10.1001/jamanetworkopen.2023.30327

eFigure 1. Flow Diagram Describing Selection of Admissions in Analytic Cohort
eFigure 2. Changes in Outcomes in Patients With Non–ST Segment Elevation Myocardial Infarction by Hospital COVID-19 Burden
eTable 1. Codes Used to Identify Patient Cohorts, Comorbidities, and COVID-19
eTable 2. Hospital Characteristics
eTable 3. Mortality at 30 d
eTable 4. Readmission at 30 d
eTable 5. Nonhome Discharges
eTable 6. Revascularization
eTable 7. Mortality at 30 d With Interaction of Race and Ethnicity and Hospital COVID-19 Burden
eTable 8. Readmission at 30 d With Interaction of Race and Ethnicity and Hospital COVID-19 Burden
eTable 9. Nonhome Discharges With Interaction of Race and Ethnicity and Hospital COVID-19 Burden
eTable 10. Revascularization With Interaction of Race and Ethnicity and Hospital COVID-19 Burden

This supplemental material has been provided by the authors to give readers additional information about their work.

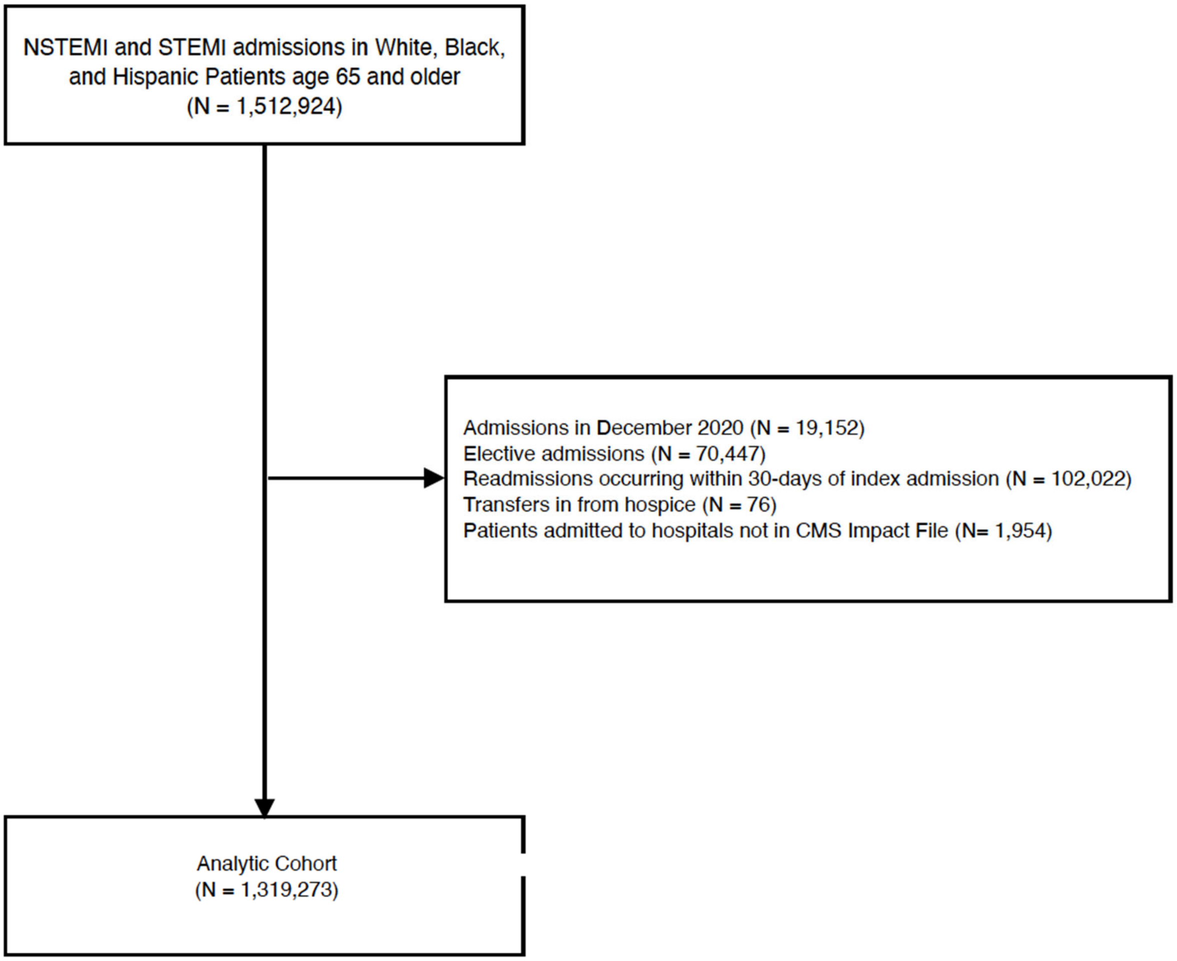

eFigure 1. Flow diagram describing the selection of cases in the analytic cohort.

© 2023 Glance LG et al. *JAMA Network Open.*

	Adjusted Rate, %	Unadjusted OR (95% CI)	Adjusted OR Patient (95% CI)	Adjusted OR Patient+ Hospital (95% CI)	P value Adjusted	Joint P value Adjusted
**Mortality**						
Prior to COVID-19	16.3	reference	reference	reference		
0-2.0%	16.0	1 (0.93 ,1.06)	0.97 (0.9 ,1.04)	0.97 (0.9 ,1.04)	0.41	
2.1- 10%	15.8	1.01 (0.91 ,1.12)	0.95 (0.85 ,1.07)	0.95 (0.85 ,1.07)	0.44	
10.1-20%	17.0	1.1 (0.96 ,1.25)	1.07 (0.92 ,1.24)	1.05 (0.9 ,1.21)	0.40	
20.1-30%	15.4	1.02 (0.85 ,1.23)	0.92 (0.75 ,1.13)	0.9 (0.73 ,1.11)	0.41	
30.1%-	19.2	1.31 (1.05 ,1.63)	1.28 (1 ,1.64)	1.23 (0.96 ,1.58)	0.050	0.05
**Readmission**						
Prior to COVID-19	15.3	reference	reference	reference		
0-2.0%	15	0.98 (0.91 ,1.04)	0.98 (0.91 ,1.05)	0.97 (0.91 ,1.04)	0.51	
2.1- 10%	15.7	1.05 (0.95 ,1.17)	1.03 (0.93 ,1.15)	1.03 (0.93 ,1.15)	0.54	
10.1-20%	16.8	1.13 (0.99 ,1.3)	1.12 (0.98 ,1.28)	1.09 (0.95 ,1.24)	0.10	
20.1-30%	16.7	1.14 (0.95 ,1.37)	1.11 (0.93 ,1.34)	1.05 (0.87 ,1.26)	0.25	
30.1%-	15.2	1.01 (0.79 ,1.3)	1 (0.78 ,1.28)	0.93 (0.72 ,1.2)	0.98	0.24
**non-Home Discharge**						
Prior to COVID-19	24.8	reference	reference	reference		
0-2.0%	24.6	1 (0.95 ,1.06)	0.98 (0.92 ,1.05)	0.99 (0.92 ,1.05)	0.64	
2.1- 10%	24	1 (0.91 ,1.1)	0.94 (0.84 ,1.05)	0.92 (0.82 ,1.03)	0.28	
10.1-20%	25	1.04 (0.93 ,1.17)	1.01 (0.88 ,1.16)	0.99 (0.86 ,1.14)	0.85	
20.1-30%	21.1	0.88 (0.73 ,1.05)	0.75 (0.61 ,0.92)	0.74 (0.6 ,0.91)	0.006	
30.1%-	24.5	1.04 (0.84 ,1.3)	0.98 (0.77 ,1.25)	0.96 (0.76 ,1.23)	0.86	0.054
**Revascularization**						
Prior to COVID-19	80.5	reference	reference	reference		
0-2.0%	79.6	0.91 (0.86 ,0.97)	0.93 (0.87 ,1)	0.94 (0.88 ,1.01)	0.04	
2.1- 10%	81	0.97 (0.88 ,1.08)	1.04 (0.93 ,1.16)	1.01 (0.9 ,1.13)	0.47	
10.1-20%	79.9	0.9 (0.79 ,1.03)	0.96 (0.83 ,1.1)	0.96 (0.83 ,1.1)	0.53	
20.1-30%	81.4	0.93 (0.77 ,1.12)	1.08 (0.88 ,1.33)	1.1 (0.89 ,1.36)	0.47	
30.1%-	79.4	0.84 (0.66 ,1.06)	0.92 (0.71 ,1.19)	1.01 (0.78 ,1.3)	0.52	0.02

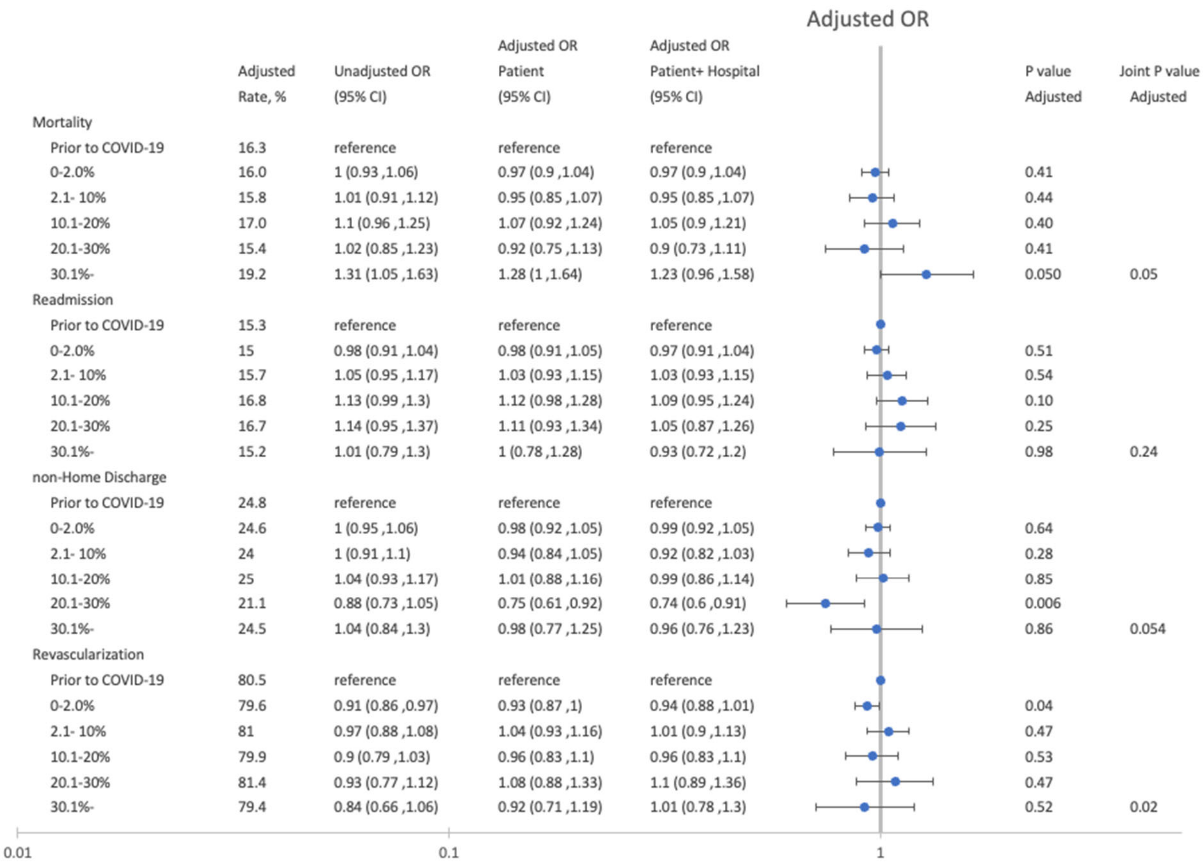

eFigure 2. Changes in Mortality, Readmissions, Non-Home Discharges, and Revascularization in STEMI Patients as a Function of the Hospital COVID-19 Burden.

The *Patient Model* was adjusted for patient risk (see text), and the *Patient + Hospital Model* was adjusted for patient risk and hospital characteristics (see text). The *Unadjusted Model* was adjusted for the patient age, race, and ethnicity. P values and adjusted rates are based on the *Patient Model*.

© 2023 Glance LG et al. *JAMA Network Open.*

AM classification	
STEMI	
Anterior wall	I210, I220
Inferior wall	I211, I221
lateral wall & other sites	I212, I228
unspecified	I213, I229
NSTEMI	I214, I222
AMI, unspecified	I219
Other types of MI	I21A
Body mass index	
Underweight	Z681
Morbid obesity	Z684
Congestive heart failure	
Diastolic	I503
Systolic	I502
Systolic & diastolic	I504
unspecified	I501, I5089, I509
Right heart failure	I5081
Biventricular failure	I5082
End-stage heart failure	I5084
Complications of AMI	
Ventricular septal defect	I232
Ventricular rupture	I233
Rupture of papillary muscle	I235
Prior cardiac surgery or procedure	
PCI	Z955, Z9861
CABG	Z951
Heart valve surgery	Z95.2; Z95.3; Z95.4
AICD	Z95810
Dialysis	Z992
Revascularization during hospitalization	
CABG	0210, 0211, 0212, 0213
PCI	0270,0271,0272, 0273, 0274
Functional status	
Wheel-chair	Z993
Supplemental oxygen	Z9981
Dependent on provider	Z74
COVID-19	U071

eTable 1. ICD-10-CM codes used to identify patient cohorts, comorbidities, and COVID-19.

© 2023 Glance LG et al. *JAMA Network Open.*

eTable 2. Hospital Characteristics

Characteristics	Hospitals, No. (%)
Total number of beds	
<50	440 (14.3)
51-149	1,117 (36.3)
150-249	651 (21.2)
250-499	651 (21.2)
500-	219 (7.1)
Resident-to-bed ratio	
0	1,995 (64.8)
>0-0.10	515 (16.7)
0.11-0.20	198 (6.4)
0.21-0.40	190 (6.2)
0.41-	180 (5.9)
Rurality	
Rural hospital	756 (24.6)
Large urban hospital	1,258 (40.9)
Other urban hospital	1,064 (34.6)
Region	
New England	129 (4.2)
Middle Atlantic	346 (11.2)
South Atlantic	555 (18)
East North Central	469 (15.2)
East South Central	280 (9.1)
West North Central	232 (7.5)
West South Central	430 (14)
Mountain	205 (6.7)
Pacific	383 (12.4)
Puerto Rico	49 (1.6)
Disproportionate share percentage (DSH)	
0-9.9%	199 (6.5)
10.0-24.9%	1,057 (34.3)
25.0-49.9%	1,473 (47.9)
50.0%-	349 (11.3)
Proportion of minority patients (AMI)	
<5%	1,071 (34.8)
5.0-9.9%	470 (15.3)
10.0-24.9	689 (22.4)
25.0-49.9%	490 (15.9)
50.0%-	358 (11.6)
Volume of AMI hospitalizations	
<200	1,521 (49.4)
200-499	550 (17.9)
500-999	644 (20.9)
1000-1999	315 (10.2)
2000-	48 (1.6)

© 2023 Glance LG et al. *JAMA Network Open.*

eTable 3. 30-day Mortality for STEMI and NSTEMI

	Unadjusted				Patient Charactistics				Patient and Hospital Characteristics			
	STEMI		NSTEMI		STEMI		NSTEMI		STEMI		NSTEMI	
	OR (95% CI)	P	OR (95% CI)	P	OR (95% CI)	P	OR (95% CI)	P	OR (95% CI)	P	OR (95% CI)	P
Race												
White	reference				reference				reference		reference	
Black	1.28 (1.23 ,1.34)	0.000	0.99 (0.97 ,1.02)	0.69	0.94 (0.9 ,0.99)	0.02	0.79 (0.77 ,0.82)	0.000	0.91 (0.87 ,0.96)	0.000	0.78 (0.76 ,0.81)	0.000
Hispanic	1.24 (1.19 ,1.3)	0.000	1.07 (1.03 ,1.11)	0.000	1.11 (1.05 ,1.18)	0.000	1.03 (0.98 ,1.09)	0.22	1.05 (1 ,1.11)	0.07	0.98 (0.94 ,1.02)	0.33
Age												
65-69	reference				reference				reference		reference	
70-74	1.16 (1.12 ,1.2)	0.000	0.97 (0.94 ,0.99)	0.02	1.24 (1.2 ,1.29)	0.000	1.17 (1.14 ,1.2)	0.000	1.24 (1.2 ,1.29)	0.000	1.17 (1.13 ,1.2)	0.000
75-79	1.52 (1.47 ,1.57)	0.000	1.28 (1.25 ,1.31)	0.000	1.59 (1.54 ,1.65)	0.000	1.45 (1.42 ,1.49)	0.000	1.59 (1.54 ,1.65)	0.000	1.45 (1.41 ,1.49)	0.000
80-84	2.13 (2.06 ,2.2)	0.000	1.74 (1.7 ,1.79)	0.000	2.18 (2.11 ,2.27)	0.000	1.9 (1.85 ,1.96)	0.000	2.18 (2.1 ,2.26)	0.000	1.9 (1.85 ,1.95)	0.000
85-89	3.17 (3.06 ,3.28)	0.000	2.53 (2.48 ,2.59)	0.000	3.18 (3.06 ,3.31)	0.000	2.65 (2.58 ,2.72)	0.000	3.18 (3.05 ,3.31)	0.000	2.64 (2.57 ,2.71)	0.000
90-94	4.77 (4.59 ,4.96)	0.000	3.71 (3.61 ,3.81)	0.000	4.84 (4.63 ,5.07)	0.000	3.82 (3.71 ,3.94)	0.000	4.84 (4.62 ,5.06)	0.000	3.79 (3.68 ,3.91)	0.000
95-	7.29 (6.87 ,7.73)	0.000	5.5 (5.32 ,5.69)	0.000	7.51 (7.01 ,8.05)	0.000	5.58 (5.36 ,5.8)	0.000	7.47 (6.97 ,8)	0.000	5.52 (5.31 ,5.74)	0.000
Hospital Covid-19 burden												
Before Covid-19	reference				reference				reference		reference	
0.0-2.0%	1 (0.93 ,1.06)	0.91	0.94 (0.9 ,0.98)	0.004	0.97 (0.9 ,1.04)	0.41	0.93 (0.89 ,0.98)	0.003	0.97 (0.9 ,1.04)	0.39	0.93 (0.89 ,0.98)	0.003
2.1-10.0%	1.01 (0.91 ,1.12)	0.86	0.99 (0.93 ,1.07)	0.86	0.95 (0.85 ,1.07)	0.44	0.96 (0.89 ,1.03)	0.27	0.95 (0.85 ,1.07)	0.43	0.97 (0.9 ,1.04)	0.42
10.1-20.0%	1.1 (0.96 ,1.25)	0.17	1.15 (1.06 ,1.25)	0.001	1.07 (0.92 ,1.24)	0.40	1.1 (1.01 ,1.21)	0.04	1.05 (0.9 ,1.21)	0.55	1.09 (1 ,1.19)	0.06
20.1-30.0%	1.02 (0.85 ,1.23)	0.83	1.25 (1.1 ,1.42)	0.001	0.92 (0.75 ,1.13)	0.41	1.21 (1.05 ,1.38)	0.007	0.9 (0.73 ,1.11)	0.32	1.19 (1.04 ,1.36)	0.01
30.1%-	1.31 (1.05 ,1.63)	0.02	1.56 (1.34 ,1.8)	0.000	1.28 (1 ,1.64)	0.05	1.51 (1.29 ,1.76)	0.000	1.23 (0.96 ,1.58)	0.10	1.46 (1.25 ,1.71)	0.000
Sex												
Male					reference				reference		reference	
Female					1.17 (1.15 ,1.2)	0.000	0.93 (0.92 ,0.95)	0.000	1.17 (1.15 ,1.2)	0.000	0.93 (0.92 ,0.95)	0.000
Urgency												
Urgent					0.91 (0.87 ,0.95)	0.000	0.87 (0.84 ,0.9)	0.000	0.91 (0.88 ,0.95)	0.000	0.87 (0.84 ,0.89)	0.000
Emergent					reference				reference		reference	
Admission source												
Community					reference				reference		reference	
Hospital					1.08 (1.03 ,1.13)	0.001	1.08 (1.04 ,1.12)	0.000	1.1 (1.05 ,1.15)	0.000	1.14 (1.1 ,1.18)	0.000
SNF/Nursing Home					2.13 (1.92 ,2.35)	0.000	1.81 (1.72 ,1.9)	0.000	2.15 (1.95 ,2.38)	0.000	1.84 (1.75 ,1.94)	0.000
Other					1.12 (1.02 ,1.22)	0.01	1.23 (1.15 ,1.31)	0.000	1.12 (1.03 ,1.22)	0.01	1.25 (1.18 ,1.33)	0.000
Dual-eligible					1.05 (1.01 ,1.09)	0.000	0.92 (0.89 ,0.94)	0.000	1.03 (1 ,1.07)	0.000	0.91 (0.89 ,0.93)	0.000
Body Mass Index												
Underweight					1.02 (0.92 ,1.13)	0.77	1.18 (1.13 ,1.24)	0.000	1.02 (0.92 ,1.13)	0.75	1.19 (1.13 ,1.24)	0.000
Morbid obesity					1.15 (1.07 ,1.23)	0.000	1 (0.96 ,1.04)	0.95	1.15 (1.07 ,1.23)	0.000	1 (0.96 ,1.04)	0.99
Myocardial infarction location												
Inferior wall					reference		NA		reference		NA	
Anterior wall					1.38 (1.35 ,1.42)	0.000	NA		1.38 (1.35 ,1.42)	0.000	NA	
Lateral wall					1.4 (1.34 ,1.47)	0.000	NA		1.4 (1.34 ,1.46)	0.000	NA	
Unspecified					1.93 (1.87 ,1.99)	0.000	NA		1.9 (1.84 ,1.96)	0.000	NA	

© 2023 Glance LG et al. *JAMA Network Open.*

	Unadjusted				Patient Charactistics				Patient and Hospital Characteristics			
	STEMI		NSTEMI		STEMI		NSTEMI		STEMI		NSTEMI	
	OR (95% CI)	P	OR (95% CI)	P	OR (95% CI)	P	OR (95% CI)	P	OR (95% CI)	P	OR (95% CI)	P
Congestive heart failure												
Systolic					1.29 (1.24 ,1.33)	0.000	2.32 (2.27 ,2.37)	0.000	1.29 (1.24 ,1.34)	0.000	2.34 (2.28 ,2.39)	0.000
Diastolic					0.88 (0.83 ,0.93)	0.000	1.42 (1.38 ,1.46)	0.000	0.88 (0.84 ,0.94)	0.000	1.43 (1.4 ,1.47)	0.000
Systolic & Diastolic					1.15 (1.08 ,1.22)	0.000	2.11 (2.04 ,2.17)	0.000	1.15 (1.08 ,1.22)	0.000	2.12 (2.06 ,2.19)	0.000
Unspecified					2.32 (2.21 ,2.44)	0.000	2.54 (2.47 ,2.62)	0.000	2.3 (2.19 ,2.41)	0.000	2.5 (2.43 ,2.58)	0.000
Right heart failure					4.23 (3.47 ,5.15)	0.000	3.1 (2.64 ,3.64)	0.000	4.24 (3.48 ,5.17)	0.000	3.12 (2.65 ,3.66)	0.000
Biventricular heart failure					4.21 (3.35 ,5.29)	0.000	2.34 (2.01 ,2.73)	0.000	4.26 (3.4 ,5.34)	0.000	2.4 (2.06 ,2.78)	0.000
End-stage heart failure					2.74 (1.89 ,3.98)	0.000	2.91 (2.5 ,3.38)	0.000	2.73 (1.88 ,3.96)	0.000	2.91 (2.5 ,3.38)	0.000
Complications of AMI												
Ventricular septal defect					20.8 (16.3 ,26.4)	0.000	24.3 (14.2 ,41.8)	0.000	20.9 (16.4 ,26.5)	0.000	24.9 (14.5 ,42.7)	0.000
LV rupture					34.8 (25.2 ,48.1)	0.000	42.1 (22.4 ,79.1)	0.000	35.2 (25.5 ,48.6)	0.000	42.1 (22.4 ,79)	0.000
Papillary muscle rupture					10.6 (7.2 ,15.7)	0.000	7.5 (4.5 ,12.5)	0.000	10.7 (7.3 ,15.8)	0.000	7.8 (4.7 ,13)	0.000
Prior procedures												
PCI					0.82 (0.8 ,0.85)	0.000	0.73 (0.72 ,0.75)	0.000	0.82 (0.8 ,0.85)	0.000	0.74 (0.72 ,0.75)	0.000
CABG					1.15 (1.1 ,1.2)	0.000	0.99 (0.97 ,1.01)	0.47	1.15 (1.09 ,1.2)	0.000	0.99 (0.97 ,1.01)	0.33
Heart valve surgery					1.37 (1.25 ,1.51)	0.000	1.02 (0.98 ,1.07)	0.27	1.38 (1.25 ,1.52)	0.000	1.03 (0.99 ,1.08)	0.12
Dialysis					2.59 (2.38 ,2.83)	0.000	2.12 (2.04 ,2.2)	0.000	2.59 (2.37 ,2.82)	0.000	2.12 (2.04 ,2.21)	0.000
AICD					0.99 (0.88 ,1.1)	0.82	0.81 (0.78 ,0.85)	0.000	0.99 (0.88 ,1.1)	0.81	0.81 (0.78 ,0.85)	0.000
COVID					2.64 (2.08 ,3.35)	0.000	2.18 (1.82 ,2.61)	0.000	2.64 (2.09 ,3.35)	0.000	2.17 (1.81 ,2.59)	0.000
Functional Status												
Wheel-chair					1.34 (1.12 ,1.6)	0.001	1.25 (1.15 ,1.36)	0.000	1.35 (1.13 ,1.61)	0.001	1.27 (1.17 ,1.38)	0.000
Supplemental oxygen					1.4 (1.29 ,1.52)	0.000	1.38 (1.34 ,1.43)	0.000	1.4 (1.29 ,1.51)	0.000	1.38 (1.33 ,1.43)	0.000
Dependent on provider					2.15 (1.81 ,2.56)	0.000	2.19 (2.03 ,2.37)	0.000	2.12 (1.78 ,2.52)	0.000	2.14 (1.98 ,2.31)	0.000
Elixhauser comorbidities												
Cardiac arrhythmias					1.57 (1.54 ,1.61)	0.000	1.17 (1.16 ,1.19)	0.000	1.58 (1.54 ,1.61)	0.000	1.18 (1.16 ,1.2)	0.000
Valvular heart disease					0.93 (0.9 ,0.97)	0.000	1.06 (1.04 ,1.08)	0.000	0.94 (0.91 ,0.97)	0.000	1.07 (1.05 ,1.09)	0.000
Pulmonary circulation					1.16 (1.1 ,1.22)	0.000	1.12 (1.09 ,1.14)	0.000	1.16 (1.1 ,1.22)	0.000	1.12 (1.09 ,1.15)	0.000
Peripheral vascular disorder					1.34 (1.3 ,1.39)	0.000	1.16 (1.14 ,1.19)	0.000	1.35 (1.3 ,1.4)	0.000	1.17 (1.15 ,1.2)	0.000
Hypertension, uncomplicated					0.66 (0.64 ,0.68)	0.000	0.66 (0.64 ,0.68)	0.000	0.66 (0.64 ,0.68)	0.000	0.66 (0.65 ,0.68)	0.000
Hypertension, complicated					0.67 (0.64 ,0.69)	0.000	0.69 (0.67 ,0.71)	0.000	0.67 (0.64 ,0.69)	0.000	0.69 (0.67 ,0.71)	0.000
Paralysis					1.59 (1.37 ,1.84)	0.000	1.63 (1.49 ,1.78)	0.000	1.58 (1.36 ,1.83)	0.000	1.63 (1.49 ,1.79)	0.000
Neurologic disorder, other					3.43 (3.3 ,3.56)	0.000	2.23 (2.18 ,2.28)	0.000	3.44 (3.31 ,3.57)	0.000	2.23 (2.18 ,2.28)	0.000
Chronic pulmonary disease					1.12 (1.09 ,1.15)	0.000	1.11 (1.09 ,1.12)	0.000	1.12 (1.08 ,1.15)	0.000	1.1 (1.08 ,1.12)	0.000
Diabetes, uncomplicated					1.21 (1.17 ,1.25)	0.000	1.07 (1.05 ,1.09)	0.000	1.21 (1.17 ,1.24)	0.000	1.06 (1.04 ,1.09)	0.000

© 2023 Glance LG et al. *JAMA Network Open.*

	Unadjusted				Patient Charactistics				Patient and Hospital Characteristics			
	STEMI		NSTEMI		STEMI		NSTEMI		STEMI		NSTEMI	
	OR (95% CI)	P	OR (95% CI)	P	OR (95% CI)	P	OR (95% CI)	P	OR (95% CI)	P	OR (95% CI)	P
Diabetes, complicated					1.29 (1.25 ,1.33)	0.000	1.11 (1.09 ,1.13)	0.000	1.29 (1.25 ,1.33)	0.000	1.11 (1.09 ,1.14)	0.000
Hypothyroidism					0.85 (0.82 ,0.87)	0.000	0.88 (0.87 ,0.9)	0.000	0.85 (0.82 ,0.87)	0.000	0.88 (0.87 ,0.9)	0.000
Renal failure					1.25 (1.2 ,1.29)	0.000	1.37 (1.34 ,1.4)	0.000	1.25 (1.21 ,1.3)	0.000	1.37 (1.35 ,1.4)	0.000
Liver disease					2.67 (2.54 ,2.82)	0.000	2.32 (2.24 ,2.4)	0.000	2.68 (2.55 ,2.83)	0.000	2.34 (2.25 ,2.42)	0.000
Peptic ulcer disease					0.8 (0.67 ,0.95)	0.01	0.85 (0.78 ,0.92)	0.000	0.8 (0.67 ,0.95)	0.01	0.86 (0.79 ,0.93)	0.000
AIDS/HIV					1.09 (0.71 ,1.7)	0.69	1.19 (0.91 ,1.56)	0.21	1.09 (0.7 ,1.7)	0.69	1.23 (0.94 ,1.61)	0.13
Lymphoma					1.33 (1.16 ,1.53)	0.000	1.25 (1.16 ,1.34)	0.000	1.34 (1.16 ,1.54)	0.000	1.26 (1.18 ,1.36)	0.000
Metastatic cancer					2.56 (2.32 ,2.83)	0.000	2.96 (2.81 ,3.11)	0.000	2.57 (2.32 ,2.83)	0.000	2.97 (2.82 ,3.13)	0.000
Solid tumor					1.59 (1.49 ,1.69)	0.000	1.56 (1.51 ,1.63)	0.000	1.59 (1.49 ,1.69)	0.000	1.57 (1.51 ,1.63)	0.000
Rheumatoid arthritis					0.93 (0.87 ,1)	0.048	0.98 (0.95 ,1.02)	0.40	0.94 (0.88 ,1)	0.06	0.99 (0.95 ,1.03)	0.62
Coagulopathy					1.4 (1.33 ,1.48)	0.000	1.3 (1.26 ,1.33)	0.000	1.41 (1.33 ,1.48)	0.000	1.3 (1.26 ,1.34)	0.000
Weight loss					1.35 (1.26 ,1.45)	0.000	1.88 (1.81 ,1.94)	0.000	1.35 (1.26 ,1.45)	0.000	1.88 (1.82 ,1.95)	0.000
Fluid and electrolyte disorder					2.62 (2.54 ,2.69)	0.000	1.94 (1.91 ,1.97)	0.000	2.61 (2.54 ,2.69)	0.000	1.94 (1.91 ,1.97)	0.000
Anemia, blood loss					0.83 (0.71 ,0.98)	0.03	0.91 (0.84 ,0.99)	0.02	0.83 (0.7 ,0.97)	0.019	0.91 (0.84 ,0.99)	0.02
Anemia, deficiency					0.73 (0.68 ,0.79)	0.000	0.85 (0.82 ,0.88)	0.000	0.73 (0.68 ,0.79)	0.000	0.86 (0.83 ,0.89)	0.000
Alcohol abuse					0.99 (0.91 ,1.07)	0.81	0.85 (0.8 ,0.89)	0.000	0.99 (0.91 ,1.07)	0.78	0.85 (0.8 ,0.89)	0.000
Drug abuse					0.74 (0.66 ,0.84)	0.000	0.77 (0.72 ,0.84)	0.000	0.74 (0.66 ,0.84)	0.000	0.78 (0.72 ,0.84)	0.000
Psychoses					1.33 (1.12 ,1.57)	0.001	1.09 (0.99 ,1.2)	0.07	1.32 (1.12 ,1.56)	0.001	1.09 (0.99 ,1.2)	0.07
Depression					0.86 (0.82 ,0.89)	0.000	0.93 (0.91 ,0.95)	0.000	0.86 (0.82 ,0.9)	0.000	0.94 (0.91 ,0.96)	0.000
Volume of AMI hospitalizations												
<200									reference		reference	
200-499									0.83 (0.78 ,0.89)	0.000	0.92 (0.89 ,0.96)	0.000
500-999									0.85 (0.79 ,0.9)	0.000	0.91 (0.88 ,0.95)	0.000
1000-1999									0.84 (0.79 ,0.9)	0.000	0.88 (0.85 ,0.92)	0.000
2000-									0.8 (0.73 ,0.87)	0.000	0.82 (0.77 ,0.88)	0.000
Proportion of minority patients (AMI)												
<5%									reference		reference	
5.0-9.9%									1 (0.95 ,1.05)	0.99	1 (0.97 ,1.04)	0.966
10.0-24.9									1.07 (1.03 ,1.11)	0.001	1.05 (1.02 ,1.09)	0.003
25.0-49.9%									1.04 (0.99 ,1.1)	0.10	1.05 (1.01 ,1.09)	0.023
50.0%-									1.21 (1.1 ,1.32)	0.000	1.24 (1.15 ,1.32)	0.000
Disproportionate share percentage (DSH)												
0-9.9%									reference		reference	
10.0-24.9%									1.03 (0.96 ,1.11)	0.37	1.03 (0.96 ,1.09)	0.41
25.0-49.9%									1.1 (1.02 ,1.18)	0.02	1.07 (1 ,1.14)	0.04
50.0%-									1.08 (0.98 ,1.19)	0.12	0.98 (0.9 ,1.06)	0.56
Resident-to-bed ratio												
0									reference		reference	
>0-0.10									0.96 (0.92 ,1)	0.04	0.97 (0.95 ,1)	0.09
0.11-0.20									0.96 (0.92 ,1.01)	0.16	0.97 (0.94 ,1.01)	0.17
0.21-0.40									0.95 (0.9 ,1)	0.07	0.9 (0.86 ,0.94)	0.000
0.41-									0.97 (0.92 ,1.03)	0.33	0.85 (0.8 ,0.89)	0.000

© 2023 Glance LG et al. *JAMA Network Open.*

	Unadjusted				Patient Charactistics				Patient and Hospital Characteristics			
	STEMI		NSTEMI		STEMI		NSTEMI		STEMI		NSTEMI	
	OR (95% CI)	P	OR (95% CI)	P	OR (95% CI)	P	OR (95% CI)	P	OR (95% CI)	P	OR (95% CI)	P
Rurality												
Rural hospital									reference		reference	
Large urban hospital									0.89 (0.84 ,0.95)	0.000	0.84 (0.8 ,0.88)	0.000
Other urban hospital									0.97 (0.91 ,1.02)	0.25	0.91 (0.87 ,0.95)	0.000
Time	1 (1 ,1)	0.008	1 (1 ,1)	0.000	1 (1 ,1)	0.000	1 (1 ,1)	0.000	1 (1 ,1)	0.000	1 (1 ,1)	0.000
Admission month												
January	reference				reference				reference		reference	
February	1.04 (0.99 ,1.09)	0.12	1.01 (0.97 ,1.04)	0.75	1.03 (0.97 ,1.09)	0.31	1.02 (0.99 ,1.05)	0.23	1.03 (0.97 ,1.08)	0.31	1.02 (0.99 ,1.05)	0.20
March	0.98 (0.93 ,1.03)	0.38	0.98 (0.94 ,1.01)	0.17	0.96 (0.91 ,1.02)	0.21	0.99 (0.95 ,1.02)	0.51	0.96 (0.91 ,1.02)	0.20	0.99 (0.96 ,1.03)	0.60
April	0.98 (0.93 ,1.03)	0.50	0.95 (0.92 ,0.98)	0.003	0.98 (0.93 ,1.04)	0.58	0.97 (0.93 ,1)	0.07	0.98 (0.93 ,1.04)	0.57	0.97 (0.94 ,1.01)	0.10
May	0.98 (0.93 ,1.03)	0.44	0.92 (0.89 ,0.96)	0.000	0.96 (0.9 ,1.02)	0.15	0.94 (0.91 ,0.97)	0.001	0.96 (0.9 ,1.01)	0.14	0.94 (0.91 ,0.98)	0.001
June	0.99 (0.94 ,1.05)	0.77	0.91 (0.88 ,0.94)	0.000	0.99 (0.93 ,1.05)	0.69	0.93 (0.9 ,0.97)	0.000	0.99 (0.93 ,1.05)	0.70	0.93 (0.9 ,0.97)	0.000
July	0.97 (0.92 ,1.02)	0.29	0.89 (0.86 ,0.92)	0.000	0.95 (0.9 ,1.01)	0.10	0.91 (0.88 ,0.95)	0.000	0.95 (0.9 ,1.01)	0.10	0.91 (0.88 ,0.95)	0.000
August	0.96 (0.91 ,1.01)	0.11	0.92 (0.89 ,0.95)	0.000	0.94 (0.89 ,1)	0.04	0.94 (0.9 ,0.97)	0.000	0.94 (0.89 ,1)	0.04	0.94 (0.91 ,0.97)	0.001
September	0.99 (0.94 ,1.04)	0.65	0.93 (0.9 ,0.96)	0.000	0.97 (0.92 ,1.03)	0.35	0.95 (0.92 ,0.98)	0.005	0.97 (0.92 ,1.03)	0.36	0.95 (0.92 ,0.99)	0.009
October	1.01 (0.96 ,1.07)	0.63	0.99 (0.95 ,1.02)	0.44	1.01 (0.95 ,1.07)	0.71	1.01 (0.98 ,1.05)	0.56	1.01 (0.96 ,1.07)	0.69	1.01 (0.98 ,1.05)	0.47
November	1.05 (0.99 ,1.1)	0.09	1.01 (0.97 ,1.04)	0.70	1.02 (0.97 ,1.08)	0.40	1.03 (0.99 ,1.06)	0.15	1.03 (0.97 ,1.08)	0.38	1.03 (0.99 ,1.07)	0.12
December	1.11 (1.05 ,1.17)	0.000	1.02 (0.99 ,1.06)	0.20	1.15 (1.08 ,1.22)	0.000	1.09 (1.05 ,1.13)	0.000	1.15 (1.08 ,1.22)	0.000	1.09 (1.05 ,1.13)	0.000
COVID-19 month												
March	reference				reference				reference		reference	
April	1.15 (1.03 ,1.29)	0.01	1.2 (1.11 ,1.3)	0.000	1.2 (1.06 ,1.35)	0.005	1.25 (1.15 ,1.35)	0.000	1.19 (1.06 ,1.35)	0.005	1.24 (1.15 ,1.35)	0.000
May	1.02 (0.9 ,1.17)	0.73	1.1 (1 ,1.21)	0.04	1.04 (0.89 ,1.2)	0.65	1.12 (1.01 ,1.23)	0.03	1.04 (0.9 ,1.21)	0.59	1.11 (1.01 ,1.23)	0.03
June	1.02 (0.9 ,1.16)	0.70	1.11 (1.03 ,1.21)	0.009	1.02 (0.89 ,1.17)	0.79	1.11 (1.02 ,1.21)	0.02	1.02 (0.89 ,1.18)	0.75	1.11 (1.02 ,1.21)	0.02
July	1.07 (0.95 ,1.2)	0.27	1.13 (1.04 ,1.22)	0.004	1.03 (0.9 ,1.17)	0.66	1.16 (1.07 ,1.26)	0.001	1.03 (0.91 ,1.17)	0.64	1.15 (1.06 ,1.26)	0.001
August	1.07 (0.94 ,1.21)	0.33	1.16 (1.06 ,1.26)	0.001	1.1 (0.96 ,1.28)	0.18	1.15 (1.05 ,1.26)	0.003	1.11 (0.96 ,1.28)	0.15	1.15 (1.05 ,1.26)	0.004
September	1.05 (0.92 ,1.2)	0.49	1.15 (1.05 ,1.26)	0.003	1.06 (0.91 ,1.23)	0.46	1.15 (1.04 ,1.27)	0.005	1.06 (0.91 ,1.24)	0.44	1.14 (1.04 ,1.26)	0.007
October	1.02 (0.89 ,1.18)	0.78	1.1 (1 ,1.21)	0.04	0.97 (0.82 ,1.14)	0.70	1.13 (1.02 ,1.24)	0.02	0.97 (0.82 ,1.14)	0.71	1.12 (1.01 ,1.24)	0.03
November	1.12 (0.98 ,1.29)	0.10	1.04 (0.94 ,1.15)	0.41	1.09 (0.93 ,1.28)	0.28	1.06 (0.96 ,1.17)	0.28	1.09 (0.94 ,1.28)	0.26	1.06 (0.96 ,1.18)	0.26

© 2023 Glance LG et al. *JAMA Network Open.*

eTable 4. 30-day Readmission for STEMI and NSTEMI

	Unadjusted				Patient Charactistics				Patient and Hospital Characteristics			
	STEMI		NSTEMI		STEMI		NSTEMI		STEMI		NSTEMI	
	OR (95% CI)	P	OR (95% CI)	P	OR (95% CI)	P	OR (95% CI)	P	OR (95% CI)	P	OR (95% CI)	P
Race												
White	reference				reference				reference		reference	
Black	1.29 (1.24 ,1.34)	0.000	1.15 (1.11 ,1.19)	0.00	1.06 (1.02 ,1.11)	0.004	0.97 (0.94 ,1.01)	0.12	1.03 (0.99 ,1.07)	0.16	0.93 (0.91 ,0.95)	0.000
Hispanic	1.21 (1.15 ,1.27)	0.000	1.13 (1.07 ,1.19)	0.000	1.05 (0.99 ,1.1)	0.086	1 (0.96 ,1.05)	0.90	0.98 (0.93 ,1.03)	0.43	0.94 (0.91 ,0.98)	0.001
Age												
65-69	reference				reference				reference		reference	
70-74	1.08 (1.05 ,1.11)	0.000	0.97 (0.96 ,0.99)	0.00	1.1 (1.07 ,1.13)	0.000	1.06 (1.04 ,1.07)	0.000	1.1 (1.06 ,1.13)	0.000	1.05 (1.03 ,1.07)	0.000
75-79	1.24 (1.21 ,1.28)	0.000	1.05 (1.04 ,1.07)	0.000	1.22 (1.18 ,1.25)	0.000	1.12 (1.1 ,1.14)	0.000	1.22 (1.18 ,1.25)	0.000	1.11 (1.09 ,1.13)	0.000
80-84	1.33 (1.29 ,1.38)	0.000	1.06 (1.04 ,1.08)	0.000	1.26 (1.21 ,1.3)	0.000	1.11 (1.09 ,1.13)	0.000	1.25 (1.21 ,1.29)	0.000	1.08 (1.06 ,1.1)	0.000
85-89	1.33 (1.28 ,1.38)	0.000	1.01 (0.99 ,1.03)	0.506	1.21 (1.16 ,1.26)	0.000	1.04 (1.02 ,1.06)	0.000	1.19 (1.14 ,1.24)	0.000	0.98 (0.96 ,1)	0.09
90-94	1.23 (1.18 ,1.29)	0.000	0.84 (0.82 ,0.86)	0.000	1.09 (1.04 ,1.15)	0.001	0.85 (0.83 ,0.88)	0.000	1.06 (1 ,1.11)	0.03	0.78 (0.76 ,0.8)	0.000
95-	0.88 (0.81 ,0.95)	0.002	0.64 (0.61 ,0.67)	0.000	0.76 (0.7 ,0.83)	0.000	0.65 (0.62 ,0.68)	0.000	0.72 (0.66 ,0.79)	0.000	0.57 (0.54 ,0.6)	0.000
Hospital Covid-19 burden												
Before Covid-19	reference				reference				reference		reference	
0.0-2.0%	0.98 (0.91 ,1.04)	0.49	0.98 (0.95 ,1.01)	0.301	0.98 (0.91 ,1.05)	0.51	0.98 (0.95 ,1.01)	0.19	0.97 (0.91 ,1.04)	0.39	0.98 (0.95 ,1.01)	0.16
2.1-10.0%	1.05 (0.95 ,1.17)	0.32	0.95 (0.9 ,1.01)	0.075	1.03 (0.93 ,1.15)	0.54	0.93 (0.88 ,0.98)	0.01	1.03 (0.93 ,1.15)	0.53	0.97 (0.92 ,1.03)	0.30
10.1-20.0%	1.13 (0.99 ,1.3)	0.07	1 (0.93 ,1.07)	0.962	1.12 (0.98 ,1.28)	0.10	0.97 (0.9 ,1.04)	0.34	1.09 (0.95 ,1.24)	0.23	0.96 (0.9 ,1.03)	0.24
20.1-30.0%	1.14 (0.95 ,1.37)	0.17	1.03 (0.93 ,1.14)	0.609	1.11 (0.93 ,1.34)	0.25	0.99 (0.89 ,1.1)	0.83	1.05 (0.87 ,1.26)	0.63	0.94 (0.85 ,1.04)	0.26
30.1%-	1.01 (0.79 ,1.3)	0.92	1.19 (1.05 ,1.35)	0.006	1 (0.78 ,1.28)	0.98	1.13 (1 ,1.28)	0.06	0.93 (0.72 ,1.2)	0.58	1.01 (0.89 ,1.14)	0.89
Sex												
Male					reference				reference		reference	
Female					1.06 (1.03 ,1.08)	0.000	0.98 (0.96 ,0.99)	0.000	1.06 (1.03 ,1.08)	0.000	0.96 (0.95 ,0.97)	0.000
Urgency												
Urgent					0.99 (0.96 ,1.03)	0.78	0.97 (0.93 ,1.02)	0.20	1.01 (0.97 ,1.05)	0.581	0.97 (0.93 ,1.01)	0.10
Emergent					reference				reference		reference	
Admission source												
Community					reference				reference		reference	
Hospital					0.83 (0.8 ,0.87)	0.000	0.67 (0.64 ,0.7)	0.000	0.94 (0.9 ,0.98)	0.003	0.84 (0.81 ,0.87)	0.000
SNF/Nursing Home					0.67 (0.6 ,0.75)	0.000	0.89 (0.85 ,0.93)	0.000	0.66 (0.59 ,0.75)	0.000	0.85 (0.81 ,0.9)	0.000
Other					0.94 (0.86 ,1.03)	0.16	0.77 (0.72 ,0.83)	0.000	0.98 (0.9 ,1.07)	0.69	0.87 (0.81 ,0.93)	0.000
Dual-eligible					1.22 (1.18 ,1.26)	0.000	1.18 (1.16 ,1.2)	0.000	1.21 (1.17 ,1.24)	0.000	1.14 (1.12 ,1.15)	0.000
Body Mass Index												
Underweight					0.9 (0.81 ,1)	0.05	0.92 (0.88 ,0.96)	0.000	0.91 (0.82 ,1.01)	0.07	0.93 (0.89 ,0.97)	0.001
Morbid obesity					1 (0.94 ,1.06)	0.96	1 (0.97 ,1.02)	0.87	1 (0.94 ,1.07)	0.88	1.01 (0.98 ,1.03)	0.64
Myocardial infarction location												
Inferior wall					reference		NA		reference		NA	
Anterior wall					1.16 (1.13 ,1.18)	0.000	NA		1.15 (1.12 ,1.18)	0.000	NA	
Lateral wall					1.1 (1.06 ,1.15)	0.000	NA		1.1 (1.05 ,1.15)	0.000	NA	
Unspecified					1.27 (1.23 ,1.32)	0.000	NA		1.2 (1.16 ,1.24)	0.000	NA	

© 2023 Glance LG et al. *JAMA Network Open.*

	Unadjusted				Patient Charactistics				Patient and Hospital Characteristics			
	STEMI		NSTEMI		STEMI		NSTEMI		STEMI		NSTEMI	
	OR (95% CI)	P	OR (95% CI)	P	OR (95% CI)	P	OR (95% CI)	P	OR (95% CI)	P	OR (95% CI)	P
Congestive heart failure												
Systolic					1.51 (1.46 ,1.56)	0.000	1.25 (1.23 ,1.28)	0.000	1.54 (1.49 ,1.59)	0.000	1.28 (1.26 ,1.3)	0.000
Diastolic					1.35 (1.28 ,1.41)	0.000	1.26 (1.24 ,1.29)	0.000	1.35 (1.29 ,1.42)	0.000	1.28 (1.25 ,1.3)	0.000
Systolic & Diastolic					1.51 (1.43 ,1.6)	0.000	1.25 (1.22 ,1.28)	0.000	1.53 (1.45 ,1.61)	0.000	1.27 (1.24 ,1.3)	0.000
Unspecified					1.27 (1.21 ,1.33)	0.000	1.33 (1.3 ,1.36)	0.000	1.24 (1.18 ,1.3)	0.000	1.26 (1.24 ,1.29)	0.000
Right heart failure					1.29 (1.05 ,1.59)	0.014	0.94 (0.82 ,1.08)	0.000	1.31 (1.07 ,1.61)	0.01	0.93 (0.81 ,1.07)	0.29
Biventricular heart failure					0.99 (0.79 ,1.24)	0.94	0.86 (0.76 ,0.97)	0.40	1.03 (0.82 ,1.3)	0.78	0.91 (0.8 ,1.03)	0.14
End-stage heart failure					0.67 (0.45 ,1)	0.048	0.65 (0.55 ,0.77)	0.02	0.67 (0.46 ,1)	0.05	0.66 (0.56 ,0.79)	0.000
Complications of AMI												
Ventricular septal defect					1.9 (1.6 ,2.3)	0.000	1.8 (1.2 ,2.9)	0.01	2 (1.7 ,2.4)	0.000	2 (1.3 ,3.3)	0.003
LV rupture					0.6 (0.4 ,0.8)	0.004	0.6 (0.4 ,1.1)	0.10	0.6 (0.4 ,0.9)	0.005	0.7 (0.4 ,1.2)	0.16
Papillary muscle rupture					1.6 (1.1 ,2.4)	0.01	1.3 (0.8 ,2.2)	0.24	1.7 (1.2 ,2.5)	0.007	1.4 (0.9 ,2.4)	0.15
Prior procedures												
PCI					1.02 (0.99 ,1.05)	0.20	1 (0.98 ,1.01)	0.77	1.02 (0.99 ,1.05)	0.21	1.01 (1 ,1.03)	0.03
CABG					0.99 (0.95 ,1.03)	0.57	0.91 (0.9 ,0.93)	0.00	0.99 (0.95 ,1.03)	0.59	0.92 (0.9 ,0.93)	0.000
Heart valve surgery					1.14 (1.03 ,1.26)	0.01	1.12 (1.08 ,1.15)	0.00	1.15 (1.03 ,1.27)	0.009	1.13 (1.1 ,1.17)	0.000
Dialysis					1.64 (1.52 ,1.78)	0.000	1.58 (1.54 ,1.62)	0.000	1.62 (1.5 ,1.75)	0.000	1.58 (1.54 ,1.62)	0.000
AICD					1.17 (1.06 ,1.28)	0.00	0.98 (0.96 ,1.01)	0.28	1.16 (1.05 ,1.28)	0.002	0.99 (0.96 ,1.02)	0.52
COVID					0.93 (0.71 ,1.22)	0.58	0.97 (0.83 ,1.13)	0.69	0.94 (0.72 ,1.24)	0.68	0.97 (0.83 ,1.13)	0.67
Functional Status												
Wheel-chair					0.92 (0.76 ,1.1)	0.36	0.91 (0.85 ,0.97)	0.005	0.92 (0.77 ,1.11)	0.39	0.92 (0.85 ,0.98)	0.02
Supplemental oxygen					1.07 (0.99 ,1.15)	0.08	1.13 (1.1 ,1.16)	0.000	1.06 (0.98 ,1.14)	0.12	1.12 (1.09 ,1.15)	0.000
Dependent on provider					0.83 (0.69 ,1.01)	0.06	0.89 (0.83 ,0.96)	0.003	0.8 (0.66 ,0.97)	0.02	0.86 (0.8 ,0.93)	0.000
Elixhauser comorbidities												
Cardiac arrhythmias					1.09 (1.07 ,1.12)	0.000	1.05 (1.04 ,1.06)	0.000	1.1 (1.07 ,1.12)	0.000	1.06 (1.05 ,1.07)	0.000
Valvular heart disease					1.18 (1.15 ,1.22)	0.000	1.17 (1.15 ,1.19)	0.000	1.2 (1.17 ,1.24)	0.000	1.21 (1.2 ,1.23)	0.000
Pulmonary circulation					1.09 (1.04 ,1.15)	0.000	1.04 (1.02 ,1.06)	0.000	1.1 (1.04 ,1.15)	0.000	1.04 (1.02 ,1.06)	0.000
Peripheral vascular disorder					1.16 (1.12 ,1.2)	0.000	1.12 (1.1 ,1.14)	0.000	1.17 (1.13 ,1.21)	0.000	1.15 (1.13 ,1.17)	0.000
Hypertension, uncomplicated					1.08 (1.05 ,1.11)	0.000	0.98 (0.96 ,1)	0.040	1.08 (1.05 ,1.12)	0.000	1.01 (0.99 ,1.02)	0.41
Hypertension, complicated					1.14 (1.1 ,1.18)	0.000	1.02 (1 ,1.04)	0.032	1.15 (1.11 ,1.19)	0.000	1.06 (1.04 ,1.08)	0.000
Paralysis					1.56 (1.37 ,1.78)	0.000	1.35 (1.26 ,1.45)	0.000	1.56 (1.37 ,1.78)	0.000	1.38 (1.28 ,1.48)	0.000
Neurologic disorder, other					0.87 (0.83 ,0.91)	0.000	0.93 (0.91 ,0.95)	0.000	0.87 (0.83 ,0.9)	0.000	0.92 (0.9 ,0.94)	0.000
Chronic pulmonary disease					1.18 (1.15 ,1.22)	0.000	1.16 (1.14 ,1.17)	0.000	1.18 (1.15 ,1.21)	0.000	1.15 (1.13 ,1.16)	0.000
Diabetes, uncomplicated					1.14 (1.11 ,1.17)	0.000	1.17 (1.16 ,1.19)	0.000	1.13 (1.1 ,1.17)	0.000	1.16 (1.15 ,1.18)	0.000

© 2023 Glance LG et al. *JAMA Network Open.*

	STEMI		NSTEMI		STEMI		NSTEMI		STEMI		NSTEMI	
	OR (95% CI)	P	OR (95% CI)	P	OR (95% CI)	P	OR (95% CI)	P	OR (95% CI)	P	OR (95% CI)	P
Diabetes, complicated					1.29 (1.25 ,1.32)	0.000	1.23 (1.22 ,1.25)	0.000	1.3 (1.26 ,1.34)	0.000	1.26 (1.24 ,1.27)	0.000
Hypothyroidism					1.01 (0.98 ,1.04)	0.69	0.99 (0.97 ,1)	0.06	1.01 (0.98 ,1.04)	0.52	0.99 (0.98 ,1.01)	0.37
Renal failure					1.12 (1.08 ,1.16)	0.000	1.11 (1.09 ,1.13)	0.000	1.12 (1.08 ,1.16)	0.000	1.11 (1.09 ,1.13)	0.000
Liver disease					0.89 (0.84 ,0.94)	0.000	0.97 (0.94 ,1)	0.09	0.89 (0.84 ,0.95)	0.000	0.98 (0.95 ,1.01)	0.270
Peptic ulcer disease					1.11 (0.96 ,1.28)	0.16	1.16 (1.09 ,1.22)	0.000	1.1 (0.96 ,1.27)	0.17	1.18 (1.11 ,1.24)	0.000
AIDS/HIV					1.19 (0.85 ,1.67)	0.30	1.09 (0.92 ,1.3)	0.32	1.19 (0.85 ,1.67)	0.30	1.11 (0.93 ,1.32)	0.25
Lymphoma					1.1 (0.95 ,1.27)	0.18	1.21 (1.15 ,1.28)	0.000	1.12 (0.97 ,1.3)	0.11	1.23 (1.17 ,1.3)	0.000
Metastatic cancer					1.13 (1.02 ,1.25)	0.02	1.03 (0.98 ,1.08)	0.21	1.14 (1.03 ,1.26)	0.01	1.02 (0.97 ,1.07)	0.35
Solid tumor					1.19 (1.11 ,1.27)	0.000	1.13 (1.09 ,1.16)	0.000	1.18 (1.11 ,1.26)	0.000	1.13 (1.1 ,1.16)	0.000
Rheumatoid arthritis					1.09 (1.03 ,1.16)	0.004	1.06 (1.04 ,1.09)	0.00	1.1 (1.04 ,1.17)	0.001	1.08 (1.06 ,1.11)	0.00
Coagulopathy					1.08 (1.03 ,1.14)	0.003	1.02 (0.99 ,1.04)	0.21	1.09 (1.03 ,1.14)	0.002	1.03 (1.01 ,1.06)	0.008
Weight loss					1.13 (1.06 ,1.21)	0.000	1 (0.97 ,1.03)	0.89	1.12 (1.05 ,1.2)	0.001	0.97 (0.94 ,1.01)	0.12
Fluid and electrolyte disorder					1.03 (1.01 ,1.06)	0.02	1.07 (1.05 ,1.08)	0.000	1.02 (0.99 ,1.05)	0.15	1.05 (1.04 ,1.06)	0.000
Anemia, blood loss					1.55 (1.37 ,1.76)	0.000	1.23 (1.17 ,1.3)	0.000	1.53 (1.35 ,1.75)	0.000	1.22 (1.16 ,1.29)	0.00
Anemia, deficiency					1.28 (1.21 ,1.36)	0.000	1.13 (1.1 ,1.16)	0.000	1.29 (1.21 ,1.37)	0.000	1.14 (1.11 ,1.17)	0.000
Alcohol abuse					1.09 (1.01 ,1.17)	0.03	1.06 (1.02 ,1.1)	0.001	1.09 (1.01 ,1.18)	0.02	1.05 (1.01 ,1.09)	0.008
Drug abuse					1.22 (1.11 ,1.34)	0.000	1.1 (1.05 ,1.15)	0.000	1.2 (1.1 ,1.32)	0.000	1.09 (1.04 ,1.14)	0.000
Psychoses					1.34 (1.15 ,1.57)	0.000	1.23 (1.16 ,1.31)	0.00	1.32 (1.13 ,1.54)	0.001	1.18 (1.11 ,1.25)	0.00
Depression					1.08 (1.04 ,1.12)	0.000	1.01 (0.99 ,1.03)	0.22	1.09 (1.05 ,1.13)	0.000	1.02 (1.01 ,1.04)	0.009
Volume of AMI hospitalizations												
<200									reference		reference	
200-499									0.78 (0.73 ,0.83)	0.000	0.59 (0.56 ,0.63)	0.000
500-999									0.65 (0.61 ,0.69)	0.000	0.43 (0.41 ,0.46)	0.000
1000-1999									0.57 (0.53 ,0.61)	0.000	0.35 (0.33 ,0.37)	0.000
2000-									0.53 (0.49 ,0.58)	0.000	0.31 (0.29 ,0.34)	0.000
Proportion of minority patients (AMI)												
<5%									reference		reference	
5.0-9.9%									1.1 (1.04 ,1.16)	0.000	1.04 (0.98 ,1.1)	0.17
10.0-24.9									1.05 (1 ,1.1)	0.05	1 (0.95 ,1.06)	0.88
25.0-49.9%									1.05 (1 ,1.11)	0.07	1.02 (0.96 ,1.07)	0.59
50.0%-									1.11 (1.01 ,1.21)	0.03	1.09 (1 ,1.19)	0.06
Disproportionate share percentage (DSH)												
0-9.9%									reference		reference	
10.0-24.9%									1.09 (1.01 ,1.17)	0.03	1 (0.91 ,1.09)	0.93
25.0-49.9%									1.06 (0.98 ,1.14)	0.12	0.96 (0.88 ,1.05)	0.39
50.0%-									1.18 (1.07 ,1.3)	0.00	0.97 (0.87 ,1.08)	0.56
Resident-to-bed ratio												
0									reference		reference	
>0-0.10									0.89 (0.85 ,0.93)	0.000	0.93 (0.89 ,0.98)	0.002
0.11-0.20									0.9 (0.85 ,0.95)	0.000	0.94 (0.89 ,1)	0.05
0.21-0.40									0.9 (0.85 ,0.95)	0.000	0.98 (0.91 ,1.05)	0.56
0.41-									0.84 (0.79 ,0.89)	0.000	0.91 (0.85 ,0.97)	0.004
Rurality												

© 2023 Glance LG et al. *JAMA Network Open.*

	Unadjusted				Patient Charactistics				Patient and Hospital Characteristics			
	STEMI		NSTEMI		STEMI		NSTEMI		STEMI		NSTEMI	
	OR (95% CI)	P	OR (95% CI)	P	OR (95% CI)	P	OR (95% CI)	P	OR (95% CI)	P	OR (95% CI)	P
Rurality												
Rural hospital									reference		reference	
Large urban hospital									0.93 (0.87 ,1)	0.048	0.96 (0.9 ,1.03)	0.28
Other urban hospital									0.84 (0.79 ,0.9)	0.000	0.83 (0.78 ,0.89)	0.000
Time	1 (1 ,1)	0.019	1 (1 ,1)	0.000	1 (1 ,1)	0.000	1 (1 ,1)	0.000	1 (1 ,1)	0.002	1 (1 ,1)	0.12
Admission month												
January	reference				reference				reference		reference	
February	0.98 (0.93 ,1.03)	0.40	0.98 (0.96 ,1)	0.11	0.98 (0.93 ,1.02)	0.32	0.99 (0.97 ,1.01)	0.22	0.98 (0.93 ,1.02)	0.33	0.99 (0.97 ,1.01)	0.35
March	0.98 (0.93 ,1.03)	0.36	0.98 (0.96 ,1)	0.08	0.98 (0.93 ,1.03)	0.36	0.98 (0.96 ,1.01)	0.18	0.97 (0.93 ,1.03)	0.31	0.99 (0.97 ,1.01)	0.43
April	0.95 (0.9 ,1)	0.07	0.97 (0.94 ,0.99)	0.004	0.95 (0.9 ,1)	0.06	0.97 (0.95 ,1)	0.04	0.95 (0.9 ,1)	0.05	0.98 (0.96 ,1.01)	0.12
May	0.97 (0.92 ,1.02)	0.18	0.97 (0.95 ,0.99)	0.013	0.96 (0.91 ,1.01)	0.12	0.98 (0.96 ,1.01)	0.128	0.96 (0.91 ,1.01)	0.13	0.99 (0.97 ,1.01)	0.40
June	0.95 (0.91 ,1)	0.07	0.97 (0.94 ,0.99)	0.005	0.96 (0.91 ,1.01)	0.12	0.98 (0.96 ,1.01)	0.142	0.96 (0.91 ,1.01)	0.11	0.99 (0.97 ,1.02)	0.54
July	1 (0.95 ,1.06)	0.88	0.94 (0.92 ,0.97)	0.000	1.01 (0.96 ,1.06)	0.77	0.96 (0.94 ,0.99)	0.003	1.01 (0.95 ,1.06)	0.84	0.97 (0.95 ,1)	0.02
August	0.99 (0.94 ,1.04)	0.76	0.94 (0.92 ,0.97)	0.000	0.99 (0.94 ,1.04)	0.70	0.96 (0.94 ,0.98)	0.001	0.99 (0.94 ,1.04)	0.71	0.97 (0.94 ,0.99)	0.006
September	0.98 (0.94 ,1.04)	0.56	0.95 (0.93 ,0.98)	0.000	0.98 (0.93 ,1.03)	0.50	0.97 (0.95 ,0.99)	0.007	0.98 (0.93 ,1.03)	0.46	0.97 (0.95 ,1)	0.03
October	0.96 (0.92 ,1.01)	0.14	0.96 (0.94 ,0.98)	0.00	0.96 (0.91 ,1.01)	0.116	0.97 (0.95 ,1)	0.02	0.96 (0.91 ,1.01)	0.11	0.97 (0.95 ,1)	0.03
November	0.93 (0.88 ,0.98)	0.01	0.94 (0.92 ,0.96)	0.00	0.92 (0.87 ,0.97)	0.00	0.95 (0.93 ,0.97)	0.00	0.92 (0.87 ,0.97)	0.001	0.95 (0.93 ,0.97)	0.000
December	0.74 (0.7 ,0.79)	0.000	0.78 (0.76 ,0.8)	0.00	0.75 (0.71 ,0.79)	0.000	0.79 (0.77 ,0.81)	0.000	0.74 (0.7 ,0.79)	0.000	0.78 (0.76 ,0.8)	0.000
COVID-19 month												
March	reference				reference				reference		reference	
April	0.83 (0.73 ,0.94)	0.00	0.8 (0.76 ,0.85)	0.000	0.83 (0.73 ,0.94)	0.003	0.81 (0.76 ,0.85)	0.000	0.83 (0.74 ,0.94)	0.004	0.79 (0.75 ,0.84)	0.000
May	0.95 (0.83 ,1.09)	0.49	0.95 (0.88 ,1.01)	0.123	0.96 (0.83 ,1.11)	0.57	0.97 (0.9 ,1.04)	0.37	0.97 (0.85 ,1.12)	0.70	0.95 (0.89 ,1.02)	0.16
June	0.99 (0.88 ,1.11)	0.86	1.01 (0.95 ,1.07)	0.819	0.99 (0.88 ,1.12)	0.88	1.02 (0.96 ,1.09)	0.55	0.99 (0.88 ,1.12)	0.93	1 (0.94 ,1.06)	0.97
July	1.03 (0.92 ,1.16)	0.59	0.99 (0.93 ,1.05)	0.743	1.03 (0.91 ,1.16)	0.68	1 (0.95 ,1.06)	0.907	1.03 (0.91 ,1.16)	0.61	0.97 (0.92 ,1.03)	0.37
August	0.94 (0.82 ,1.07)	0.32	1.01 (0.94 ,1.08)	0.74	0.94 (0.82 ,1.07)	0.35	1.02 (0.95 ,1.1)	0.533	0.95 (0.83 ,1.09)	0.47	1 (0.94 ,1.07)	0.97
September	0.81 (0.71 ,0.93)	0.00	1.03 (0.96 ,1.1)	0.381	0.81 (0.71 ,0.93)	0.00	1.05 (0.98 ,1.12)	0.176	0.81 (0.71 ,0.93)	0.003	1.02 (0.96 ,1.09)	0.52
October	1.03 (0.89 ,1.18)	0.70	1.07 (1 ,1.15)	0.06	1.03 (0.89 ,1.19)	0.70	1.09 (1.01 ,1.16)	0.02	1.04 (0.9 ,1.2)	0.60	1.06 (0.99 ,1.14)	0.10
November	0.92 (0.79 ,1.06)	0.24	0.97 (0.9 ,1.04)	0.378	0.91 (0.79 ,1.06)	0.22	0.99 (0.92 ,1.07)	0.80	0.92 (0.8 ,1.07)	0.29	0.97 (0.9 ,1.04)	0.34

© 2023 Glance LG et al. *JAMA Network Open.*

eTable 5. Non-home Discharges for STEMI and NSTEMI

	Unadjusted				Patient Charactistics				Patient and Hospital Characteristics			
	STEMI		NSTEMI		STEMI		NSTEMI		STEMI		NSTEMI	
	OR (95% CI)	P	OR (95% CI)	P	OR (95% CI)	P	OR (95% CI)	P	OR (95% CI)	P	OR (95% CI)	P
Race												
White	reference				reference				reference		reference	
Black	1.43 (1.38 ,1.49)	0.000	1.13 (1.1 ,1.16)	0.000	0.95 (0.91 ,1)	0.034	0.78 (0.76 ,0.8)	0.000	0.93 (0.89 ,0.97)	0.001	0.77 (0.75 ,0.8)	0.000
Hispanic	1.09 (1.04 ,1.15)	0.000	0.82 (0.76 ,0.87)	0.000	0.86 (0.81 ,0.9)	0.000	0.65 (0.62 ,0.68)	0.00	0.84 (0.8 ,0.88)	0.000	0.65 (0.63 ,0.67)	0.00
Age												
65-69	reference				reference				reference		reference	
70-74	1.21 (1.18 ,1.25)	0.000	1.1 (1.08 ,1.12)	0.000	1.33 (1.29 ,1.38)	0.000	1.31 (1.29 ,1.34)	0.000	1.33 (1.29 ,1.38)	0.000	1.32 (1.29 ,1.34)	0.000
75-79	1.73 (1.68 ,1.78)	0.000	1.49 (1.46 ,1.52)	0.000	1.89 (1.83 ,1.95)	0.000	1.76 (1.73 ,1.8)	0.000	1.89 (1.83 ,1.95)	0.000	1.77 (1.74 ,1.8)	0.000
80-84	2.62 (2.55 ,2.7)	0.000	2.08 (2.04 ,2.12)	0.000	2.83 (2.74 ,2.93)	0.000	2.44 (2.39 ,2.49)	0.000	2.84 (2.75 ,2.93)	0.000	2.45 (2.4 ,2.51)	0.000
85-89	4.24 (4.11 ,4.38)	0.000	3.01 (2.94 ,3.07)	0.000	4.47 (4.3 ,4.64)	0.000	3.4 (3.33 ,3.48)	0.000	4.49 (4.32 ,4.66)	0.000	3.45 (3.37 ,3.53)	0.000
90-94	6.75 (6.49 ,7.02)	0.000	4.38 (4.28 ,4.5)	0.000	7.03 (6.71 ,7.37)	0.000	4.84 (4.7 ,4.98)	0.000	7.1 (6.78 ,7.45)	0.000	4.93 (4.79 ,5.07)	0.000
95-	10.61 (9.98 ,11.28)	0.000	6.89 (6.67 ,7.12)	0.000	10.95 (10.2 ,11.76)	0.000	7.32 (7.05 ,7.6)	0.000	11.13 (10.36 ,11.95)	0.000	7.51 (7.23 ,7.8)	0.000
Hospital Covid-19 burden												
Before Covid-19	reference				reference				reference		reference	
0.0-2.0%	1 (0.95 ,1.06)	0.89	1.01 (0.98 ,1.04)	0.50	0.98 (0.92 ,1.05)	0.64	1 (0.97 ,1.04)	0.892	0.99 (0.92 ,1.05)	0.65	1 (0.97 ,1.04)	0.822
2.1-10.0%	1 (0.91 ,1.1)	0.99	1.05 (0.99 ,1.11)	0.08	0.94 (0.84 ,1.05)	0.28	1.01 (0.95 ,1.07)	0.80	0.92 (0.82 ,1.03)	0.14	0.98 (0.92 ,1.04)	0.492
10.1-20.0%	1.04 (0.93 ,1.17)	0.48	1.1 (1.03 ,1.18)	0.006	1.01 (0.88 ,1.16)	0.85	1.05 (0.97 ,1.13)	0.21	0.99 (0.86 ,1.14)	0.92	1.03 (0.95 ,1.11)	0.48
20.1-30.0%	0.88 (0.73 ,1.05)	0.15	1.1 (0.99 ,1.22)	0.08	0.75 (0.61 ,0.92)	0.01	1.03 (0.92 ,1.15)	0.64	0.74 (0.6 ,0.91)	0.00	1.02 (0.91 ,1.14)	0.79
30.1%-	1.04 (0.84 ,1.3)	0.70	1.37 (1.2 ,1.57)	0.000	0.98 (0.77 ,1.25)	0.86	1.32 (1.15 ,1.52)	0.000	0.96 (0.76 ,1.23)	0.77	1.33 (1.16 ,1.53)	0.000
Sex												
Male					reference				reference		reference	
Female					1.31 (1.28 ,1.34)	0.000	1.08 (1.06 ,1.09)	0.000	1.32 (1.29 ,1.34)	0.000	1.08 (1.06 ,1.09)	0.000
Urgency												
Urgent					0.96 (0.92 ,1.01)	0.088	0.92 (0.88 ,0.95)	0.000	0.96 (0.92 ,1.01)	0.09	0.92 (0.88 ,0.95)	0.000
Emergent					reference				reference		reference	
Admission source												
Community					reference				reference		reference	
Hospital					1.18 (1.13 ,1.24)	0	1.31 (1.26 ,1.37)	0.000	1.16 (1.11 ,1.22)	0.000	1.29 (1.24 ,1.34)	0.000
SNF/Nursing Home					5.07 (4.43 ,5.81)	0.000	4.95 (4.65 ,5.26)	0.000	5.1 (4.44 ,5.87)	0.000	5.01 (4.71 ,5.33)	0.000
Other					1.25 (1.14 ,1.38)	0.00	1.47 (1.37 ,1.57)	0.000	1.24 (1.13 ,1.36)	0.000	1.45 (1.36 ,1.55)	0.000
Dual-eligible					1.54 (1.49 ,1.58)	0.000	1.64 (1.61 ,1.67)	0.000	1.53 (1.49 ,1.58)	0.000	1.65 (1.62 ,1.69)	0.000
Body Mass Index												
Underweight					0.95 (0.86 ,1.06)	0.38	1.1 (1.05 ,1.15)	0.000	0.95 (0.86 ,1.06)	0.36	1.1 (1.06 ,1.15)	0.000
Morbid obesity					1.51 (1.43 ,1.6)	0.000	1.42 (1.38 ,1.47)	0	1.52 (1.43 ,1.61)	0.000	1.43 (1.39 ,1.47)	0.00
Myocardial infarction location												
Inferior wall					reference		NA		reference		NA	
Anterior wall					1.2 (1.18 ,1.23)	0.000	NA		1.21 (1.18 ,1.24)	0.000	NA	
Lateral wall					1.23 (1.18 ,1.28)	0.000	NA		1.23 (1.19 ,1.28)	0.000	NA	
Unspecified					1.62 (1.57 ,1.67)	0.000	NA		1.63 (1.58 ,1.69)	0.000	NA	

© 2023 Glance LG et al. *JAMA Network Open.*

	Unadjusted				Patient Charactistics				Patient and Hospital Characteristics			
	STEMI		NSTEMI		STEMI		NSTEMI		STEMI		NSTEMI	
	OR (95% CI)	P	OR (95% CI)	P	OR (95% CI)	P	OR (95% CI)	P	OR (95% CI)	P	OR (95% CI)	P
Congestive heart failure												
Systolic					1.93 (1.86 ,1.99)	0.000	2.13 (2.09 ,2.17)	0.000	1.92 (1.86 ,1.99)	0.000	2.13 (2.09 ,2.17)	0.000
Diastolic					1.42 (1.35 ,1.49)	0.000	1.61 (1.58 ,1.65)	0.000	1.42 (1.35 ,1.49)	0.000	1.62 (1.59 ,1.65)	0.000
Systolic & Diastolic					1.91 (1.8 ,2.02)	0.000	2.13 (2.08 ,2.18)	0.000	1.91 (1.8 ,2.02)	0.000	2.13 (2.08 ,2.18)	0.000
Unspecified					2.26 (2.16 ,2.37)	0.000	1.82 (1.78 ,1.87)	0.000	2.27 (2.17 ,2.38)	0.000	1.84 (1.79 ,1.89)	0.000
Right heart failure					3.92 (3.17 ,4.86)	0.000	2.7 (2.34 ,3.12)	0.000	3.91 (3.16 ,4.84)	0.000	2.73 (2.36 ,3.15)	0.000
Biventricular heart failure					3.69 (2.96 ,4.61)	0.000	1.82 (1.59 ,2.08)	0.000	3.65 (2.93 ,4.56)	0.000	1.82 (1.6 ,2.08)	0.000
End-stage heart failure					2.88 (1.93 ,4.31)	0.000	2.91 (2.5 ,3.4)	0.000	2.88 (1.93 ,4.31)	0.000	2.91 (2.49 ,3.4)	0.000
Complications of AMI												
Ventricular septal defect					7.91 (6.37 ,9.82)	0.000	4.62 (2.75 ,7.76)	0.000	7.82 (6.3 ,9.71)	0.000	4.52 (2.71 ,7.55)	0.000
LV rupture					38.1 (25.8 ,56.4)	0.000	25.2 (13.5 ,47)	0.000	38.4 (26 ,56.9)	0.000	25.2 (13.5 ,47)	0.000
Papillary muscle rupture					9.44 (6.06 ,14.7)	0.000	9.86 (5.6 ,17.37)	0.000	9.31 (5.96 ,14.53)	0.000	9.92 (5.65 ,17.41)	0.000
Prior procedures												
PCI					0.8 (0.77 ,0.82)	0.000	0.66 (0.65 ,0.67)	0.000	0.8 (0.77 ,0.82)	0.000	0.66 (0.65 ,0.67)	0.000
CABG					0.92 (0.89 ,0.96)	0.000	0.73 (0.72 ,0.75)	0.00	0.92 (0.89 ,0.96)	0.000	0.73 (0.72 ,0.75)	0.000
Heart valve surgery					1.05 (0.95 ,1.15)	0.325	0.85 (0.82 ,0.89)	0.00	1.05 (0.95 ,1.15)	0.34	0.85 (0.82 ,0.88)	0.000
Dialysis					2.23 (2.05 ,2.43)	0.000	1.77 (1.71 ,1.83)	0.000	2.24 (2.06 ,2.44)	0.000	1.77 (1.71 ,1.83)	0.000
AICD					0.74 (0.67 ,0.82)	0.00	0.66 (0.63 ,0.68)	0.000	0.74 (0.67 ,0.82)	0.000	0.66 (0.63 ,0.68)	0.000
COVID					2.77 (2.19 ,3.51)	0.000	2.33 (2 ,2.71)	0.000	2.78 (2.19 ,3.52)	0.000	2.34 (2.01 ,2.73)	0.000
Functional Status												
Wheel-chair					1.73 (1.45 ,2.07)	0	1.73 (1.61 ,1.85)	0.000	1.73 (1.45 ,2.07)	0.000	1.72 (1.6 ,1.84)	0.000
Supplemental oxygen					1.29 (1.2 ,1.4)	0.000	1.08 (1.05 ,1.11)	0.000	1.3 (1.2 ,1.4)	0.000	1.09 (1.06 ,1.12)	0.000
Dependent on provider					2.01 (1.68 ,2.41)	0.000	2.18 (2.01 ,2.36)	0.000	2.01 (1.67 ,2.41)	0.000	2.19 (2.02 ,2.38)	0.000
Elixhauser comorbidities												
Cardiac arrhythmias					1.62 (1.58 ,1.65)	0.000	1.17 (1.15 ,1.18)	0.000	1.62 (1.58 ,1.65)	0.000	1.17 (1.15 ,1.18)	0.000
Valvular heart disease					0.95 (0.92 ,0.99)	0.005	1.04 (1.02 ,1.05)	0.000	0.95 (0.92 ,0.98)	0.003	1.03 (1.02 ,1.05)	0.000
Pulmonary circulation					1.17 (1.11 ,1.23)	0.000	1.14 (1.11 ,1.16)	0.000	1.17 (1.11 ,1.23)	0.000	1.14 (1.11 ,1.16)	0.000
Peripheral vascular disorder					1.32 (1.27 ,1.37)	0.000	1.1 (1.08 ,1.13)	0.000	1.32 (1.27 ,1.37)	0.000	1.1 (1.08 ,1.12)	0.000
Hypertension, uncomplicated					0.74 (0.72 ,0.76)	0.000	0.78 (0.77 ,0.8)	0.000	0.74 (0.72 ,0.76)	0.000	0.78 (0.76 ,0.79)	0.000
Hypertension, complicated					0.79 (0.76 ,0.81)	0.000	0.84 (0.82 ,0.85)	0.000	0.78 (0.75 ,0.81)	0.000	0.83 (0.81 ,0.85)	0.000
Paralysis					3.73 (3.19 ,4.37)	0.000	2.95 (2.74 ,3.19)	0.000	3.74 (3.19 ,4.38)	0.000	2.95 (2.73 ,3.19)	0.000
Neurologic disorder, other					4.84 (4.66 ,5.03)	0.000	2.93 (2.88 ,2.99)	0.000	4.85 (4.67 ,5.04)	0.000	2.94 (2.88 ,2.99)	0.000
Chronic pulmonary disease					1.11 (1.08 ,1.14)	0.000	1.03 (1.02 ,1.05)	0.000	1.11 (1.08 ,1.14)	0.000	1.03 (1.02 ,1.05)	0.000
Diabetes, uncomplicated					1.2 (1.17 ,1.23)	0.000	1.04 (1.02 ,1.06)	0.000	1.2 (1.17 ,1.24)	0.000	1.04 (1.03 ,1.06)	0.000

© 2023 Glance LG et al. *JAMA Network Open.*

	Unadjusted				Patient Charactistics				Patient and Hospital Characteristics			
	STEMI		NSTEMI		STEMI		NSTEMI		STEMI		NSTEMI	
	OR (95% CI)	P	OR (95% CI)	P	OR (95% CI)	P	OR (95% CI)	P	OR (95% CI)	P	OR (95% CI)	P
Diabetes, complicated					1.43 (1.39 ,1.47)	0.000	1.3 (1.28 ,1.32)	0.000	1.42 (1.38 ,1.47)	0.000	1.3 (1.28 ,1.32)	0.000
Hypothyroidism					0.93 (0.9 ,0.96)	0.000	0.97 (0.96 ,0.98)	0.000	0.93 (0.91 ,0.96)	0.000	0.97 (0.96 ,0.98)	0.000
Renal failure					1.25 (1.21 ,1.29)	0.000	1.17 (1.15 ,1.19)	0.000	1.25 (1.21 ,1.29)	0.000	1.18 (1.16 ,1.2)	0.000
Liver disease					2.55 (2.42 ,2.69)	0.000	1.69 (1.64 ,1.75)	0.000	2.55 (2.42 ,2.69)	0.000	1.69 (1.64 ,1.75)	0.000
Peptic ulcer disease					1.08 (0.94 ,1.24)	0.288	1.12 (1.06 ,1.19)	0.000	1.08 (0.94 ,1.24)	0.29	1.12 (1.05 ,1.19)	0.000
AIDS/HIV					0.93 (0.63 ,1.37)	0.71	0.96 (0.76 ,1.2)	0.71	0.92 (0.62 ,1.37)	0.69	0.96 (0.76 ,1.2)	0.704
Lymphoma					1.19 (1.04 ,1.36)	0.013	0.99 (0.93 ,1.05)	0.656	1.18 (1.03 ,1.35)	0.02	0.98 (0.93 ,1.05)	0.626
Metastatic cancer					1.93 (1.75 ,2.12)	0.000	1.86 (1.77 ,1.95)	0.000	1.93 (1.75 ,2.12)	0.000	1.87 (1.78 ,1.96)	0.000
Solid tumor					1.35 (1.27 ,1.44)	0.000	1.2 (1.17 ,1.24)	0.000	1.35 (1.27 ,1.44)	0.000	1.2 (1.17 ,1.24)	0.000
Rheumatoid arthritis					0.95 (0.9 ,1.01)	0.098	1.04 (1.01 ,1.07)	0.01	0.95 (0.9 ,1.01)	0.10	1.04 (1.01 ,1.07)	0.007
Coagulopathy					1.69 (1.61 ,1.78)	0.000	1.41 (1.38 ,1.44)	0.000	1.69 (1.6 ,1.77)	0.000	1.4 (1.37 ,1.43)	0.000
Weight loss					2.35 (2.19 ,2.53)	0.000	2.5 (2.41 ,2.58)	0.000	2.36 (2.19 ,2.54)	0.000	2.51 (2.43 ,2.6)	0.000
Fluid and electrolyte disorder					2.58 (2.51 ,2.65)	0.000	1.86 (1.84 ,1.89)	0.000	2.58 (2.51 ,2.65)	0.000	1.86 (1.84 ,1.89)	0.000
Anemia, blood loss					1.26 (1.09 ,1.46)	0.00	1.21 (1.15 ,1.29)	0.00	1.26 (1.09 ,1.47)	0.002	1.22 (1.15 ,1.29)	0.000
Anemia, deficiency					0.97 (0.9 ,1.04)	0.346	1.05 (1.02 ,1.08)	0.000	0.97 (0.9 ,1.03)	0.34	1.05 (1.03 ,1.08)	0.000
Alcohol abuse					1.17 (1.08 ,1.26)	0.00	1.19 (1.15 ,1.24)	0.000	1.16 (1.08 ,1.25)	0.000	1.2 (1.15 ,1.24)	0.000
Drug abuse					0.93 (0.85 ,1.03)	0.162	0.9 (0.85 ,0.95)	0.000	0.93 (0.85 ,1.03)	0.1670	0.9 (0.85 ,0.95)	0.000
Psychoses					2.36 (2.02 ,2.77)	0.000	2.32 (2.17 ,2.49)	0.000	2.37 (2.02 ,2.78)	0.000	2.34 (2.19 ,2.51)	0.000
Depression					1.2 (1.16 ,1.25)	0.000	1.28 (1.26 ,1.31)	0.000	1.2 (1.16 ,1.25)	0.000	1.28 (1.26 ,1.31)	0.000
Volume of AMI hospitalizations												
<200									reference		reference	
200-499									0.98 (0.91 ,1.05)	0.55	1.04 (1 ,1.09)	0.071
500-999									1.09 (1.02 ,1.16)	0.01	1.18 (1.13 ,1.23)	0.000
1000-1999									1.12 (1.05 ,1.2)	0.001	1.23 (1.17 ,1.29)	0.000
2000-									1.07 (0.98 ,1.18)	0.14	1.13 (1.04 ,1.23)	0.003
Proportion of minority patients (AMI)												
<5%									reference		reference	
5.0-9.9%									1.05 (0.99 ,1.11)	0.09	1.01 (0.97 ,1.06)	0.62
10.0-24.9									1.17 (1.11 ,1.23)	0.000	1.1 (1.05 ,1.15)	0
25.0-49.9%									1.05 (0.98 ,1.11)	0.14	1.02 (0.97 ,1.08)	0.398
50.0%-									1.13 (1.03 ,1.24)	0.007	1.03 (0.94 ,1.13)	0.48
Disproportionate share percentage (DSH)												
0-9.9%									reference		reference	
10.0-24.9%									1.02 (0.94 ,1.1)	0.68	1.13 (1.05 ,1.21)	0.00
25.0-49.9%									1.03 (0.95 ,1.12)	0.49	1.14 (1.06 ,1.23)	0.00
50.0%-									1.03 (0.93 ,1.15)	0.56	1.15 (1.04 ,1.27)	0.01
Resident-to-bed ratio												
0									reference		reference	
>0-0.10									1 (0.96 ,1.05)	0.88	1 (0.96 ,1.04)	0.903
0.11-0.20									1.01 (0.95 ,1.07)	0.87	1.03 (0.98 ,1.08)	0.285
0.21-0.40									1 (0.94 ,1.06)	0.95	0.99 (0.93 ,1.05)	0.714
0.41-									1.07 (1 ,1.14)	0.04	0.95 (0.9 ,1)	0.069

© 2023 Glance LG et al. *JAMA Network Open.*

	Unadjusted				Patient Charactistics				Patient and Hospital Characteristics			
	STEMI		NSTEMI		STEMI		NSTEMI		STEMI		NSTEMI	
	OR (95% CI)	P	OR (95% CI)	P	OR (95% CI)	P	OR (95% CI)	P	OR (95% CI)	P	OR (95% CI)	P
Rurality												
Rural hospital									reference		reference	
Large urban hospital									0.99 (0.92 ,1.07)	0.850	1.08 (1.02 ,1.15)	0.000
Other urban hospital									1.05 (0.98 ,1.13)	0.14	1.08 (1.01 ,1.14)	0.077
Time	1 (1 ,1)	0	0.99 (0.99 ,1)	0.000	0.99 (0.99 ,0.99)	0.000	0.99 (0.99 ,0.99)	0.000	0.99 (0.99 ,0.99)	0.000	0.99 (0.99 ,0.99)	0.000
Admission month												
January	reference				reference				reference		reference	
February	1.05 (1.01 ,1.09)	0.02	0.97 (0.95 ,0.99)	0.01	1.05 (1 ,1.1)	0.05	0.98 (0.95 ,1)	0.09	1.05 (1 ,1.1)	0.05	0.98 (0.95 ,1)	0.08
March	1.01 (0.97 ,1.06)	0.50	0.97 (0.95 ,0.99)	0.01	1 (0.95 ,1.05)	1.00	0.98 (0.96 ,1.01)	0.16	1 (0.95 ,1.05)	0.99	0.98 (0.96 ,1.01)	0.15
April	1 (0.95 ,1.04)	0.83	0.94 (0.92 ,0.97)	0	1 (0.95 ,1.05)	0.86	0.96 (0.93 ,0.98)	0.00	1 (0.95 ,1.05)	0.88	0.96 (0.93 ,0.98)	0.00
May	1.01 (0.97 ,1.06)	0.54	0.92 (0.9 ,0.95)	0.000	0.99 (0.94 ,1.04)	0.78	0.93 (0.91 ,0.96)	0	0.99 (0.95 ,1.04)	0.80	0.93 (0.91 ,0.96)	0.000
June	1.01 (0.96 ,1.05)	0.76	0.92 (0.9 ,0.94)	0.000	1 (0.95 ,1.05)	0.94	0.93 (0.9 ,0.95)	0.000	1 (0.95 ,1.06)	0.88	0.93 (0.9 ,0.95)	0.000
July	1.01 (0.97 ,1.05)	0.68	0.92 (0.9 ,0.94)	0.000	0.99 (0.94 ,1.04)	0.78	0.94 (0.91 ,0.96)	0.000	1 (0.95 ,1.05)	0.85	0.94 (0.91 ,0.96)	0.000
August	0.96 (0.92 ,1.01)	0.12	0.94 (0.92 ,0.96)	0.000	0.94 (0.89 ,0.99)	0.02	0.96 (0.93 ,0.98)	0.001	0.94 (0.89 ,0.99)	0.02	0.96 (0.93 ,0.98)	0.001
September	1 (0.96 ,1.05)	0.97	0.95 (0.93 ,0.97)	0.000	0.98 (0.93 ,1.03)	0.40	0.96 (0.94 ,0.99)	0.007	0.98 (0.93 ,1.03)	0.45	0.96 (0.94 ,0.99)	0.01
October	1.06 (1.02 ,1.11)	0.01	0.97 (0.95 ,0.99)	0.02	1.07 (1.02 ,1.12)	0.01	0.99 (0.97 ,1.02)	0.55	1.07 (1.02 ,1.13)	0.01	0.99 (0.97 ,1.02)	0.66
November	1.06 (1.01 ,1.1)	0.02	0.96 (0.93 ,0.98)	0.00	1.04 (0.99 ,1.09)	0.15	0.97 (0.95 ,1)	0.05	1.04 (0.99 ,1.09)	0.12	0.97 (0.95 ,1)	0.05
December	0.94 (0.9 ,0.99)	0.011	0.82 (0.8 ,0.84)	0.00	0.95 (0.9 ,1.01)	0.081	0.85 (0.83 ,0.88)	0.000	0.96 (0.91 ,1.01)	0.102	0.85 (0.83 ,0.88)	0.00
COVID-19 month												
March	reference				reference				reference		reference	
April	0.95 (0.86 ,1.05)	0.32	0.87 (0.82 ,0.93)	0.000	0.96 (0.85 ,1.08)	0.466	0.88 (0.83 ,0.94)	0.000	0.96 (0.85 ,1.08)	0.481	0.89 (0.83 ,0.95)	0.000
May	0.87 (0.77 ,0.98)	0.03	0.82 (0.76 ,0.88)	0	0.84 (0.73 ,0.98)	0.02	0.82 (0.76 ,0.88)	0.00	0.86 (0.74 ,0.99)	0.036	0.83 (0.77 ,0.9)	0.000
June	0.88 (0.79 ,0.98)	0.03	0.84 (0.79 ,0.89)	0	0.83 (0.73 ,0.95)	0.01	0.83 (0.78 ,0.89)	0.00	0.84 (0.74 ,0.96)	0.01	0.84 (0.79 ,0.9)	0.000
July	0.95 (0.85 ,1.05)	0.32	0.86 (0.81 ,0.91)	0	0.89 (0.78 ,1.01)	0.06	0.87 (0.81 ,0.93)	0	0.9 (0.79 ,1.02)	0.09	0.88 (0.83 ,0.95)	0.000
August	0.94 (0.84 ,1.05)	0.25	0.86 (0.8 ,0.92)	0	0.95 (0.83 ,1.08)	0.42	0.83 (0.77 ,0.9)	0	0.96 (0.84 ,1.1)	0.55	0.85 (0.79 ,0.91)	0.000
September	0.94 (0.83 ,1.06)	0.30	0.97 (0.9 ,1.04)	0.348	0.92 (0.79 ,1.06)	0.25	0.96 (0.89 ,1.03)	0.256	0.93 (0.81 ,1.08)	0.34	0.98 (0.9 ,1.05)	0.53
October	0.97 (0.85 ,1.1)	0.65	0.87 (0.8 ,0.94)	0.00	0.91 (0.78 ,1.05)	0.19	0.87 (0.8 ,0.94)	0.00	0.92 (0.79 ,1.06)	0.24	0.89 (0.82 ,0.96)	0.005
November	0.95 (0.84 ,1.07)	0.39	0.85 (0.79 ,0.92)	0	0.88 (0.76 ,1.01)	0.07	0.85 (0.79 ,0.93)	0.00	0.89 (0.77 ,1.02)	0.10	0.87 (0.8 ,0.95)	0.001

© 2023 Glance LG et al. *JAMA Network Open.*

eTable 6. Revascularization after STEMI and NSTEMI

	Unadjusted				Patient Charactistics				Patient and Hospital Characteristics			
	STEMI		NSTEMI		STEMI		NSTEMI		STEMI		NSTEMI	
	OR (95% CI)	P	OR (95% CI)	P	OR (95% CI)	P	OR (95% CI)	P	OR (95% CI)	P	OR (95% CI)	P
Race												
White	reference				reference				reference		reference	
Black	0.55 (0.53 ,0.58)	0.000	0.56 (0.54 ,0.59)	0.000	0.72 (0.69 ,0.76)	0.000	0.69 (0.66 ,0.72)	0.000	0.76 (0.72 ,0.8)	0.000	0.73 (0.71 ,0.75)	0.000
Hispanic	0.65 (0.59 ,0.71)	0.000	0.64 (0.59 ,0.69)	0.000	0.73 (0.66 ,0.82)	0.000	0.69 (0.62 ,0.76)	0.000	0.81 (0.75 ,0.88)	0.000	0.81 (0.76 ,0.86)	0.000
Age												
65-69	reference				reference				reference		reference	
70-74	0.92 (0.89 ,0.95)	0.000	0.93 (0.92 ,0.94)	0.000	0.9 (0.87 ,0.93)	0.000	0.91 (0.89 ,0.92)	0.000	0.9 (0.87 ,0.93)	0.000	0.91 (0.9 ,0.92)	0.000
75-79	0.72 (0.7 ,0.74)	0.000	0.79 (0.78 ,0.8)	0.000	0.76 (0.74 ,0.78)	0.000	0.81 (0.79 ,0.82)	0.000	0.76 (0.74 ,0.79)	0.000	0.81 (0.8 ,0.82)	0.000
80-84	0.53 (0.51 ,0.55)	0.000	0.6 (0.59 ,0.61)	0.000	0.59 (0.57 ,0.61)	0.000	0.65 (0.64 ,0.66)	0.000	0.59 (0.57 ,0.62)	0.000	0.66 (0.65 ,0.67)	0.000
85-89	0.33 (0.31 ,0.34)	0.000	0.38 (0.37 ,0.39)	0.000	0.38 (0.36 ,0.39)	0.000	0.43 (0.42 ,0.44)	0.000	0.38 (0.37 ,0.4)	0.000	0.44 (0.43 ,0.45)	0.000
90-94	0.17 (0.16 ,0.17)	0.000	0.17 (0.17 ,0.18)	0.000	0.19 (0.18 ,0.2)	0.000	0.2 (0.2 ,0.21)	0.000	0.2 (0.19 ,0.21)	0.000	0.21 (0.2 ,0.22)	0.000
95-	0.07 (0.07 ,0.08)	0.000	0.06 (0.05 ,0.06)	0.000	0.09 (0.08 ,0.09)	0.000	0.07 (0.07 ,0.08)	0.000	0.09 (0.08 ,0.1)	0.000	0.08 (0.07 ,0.08)	0.000
Hospital Covid-19 burden												
Before Covid-19	reference				reference				reference		reference	
0.0-2.0%	0.91 (0.86 ,0.97)	0.004	0.95 (0.93 ,0.98)	0.001	0.93 (0.87 ,1)	0.04	0.96 (0.93 ,0.99)	0.006	0.94 (0.88 ,1.01)	0.09	0.96 (0.93 ,0.99)	0.01
2.1-10.0%	0.97 (0.88 ,1.08)	0.58	0.96 (0.91 ,1.02)	0.20	1.04 (0.93 ,1.16)	0.47	1.01 (0.96 ,1.07)	0.73	1.01 (0.9 ,1.13)	0.87	0.94 (0.89 ,0.99)	0.02
10.1-20.0%	0.9 (0.79 ,1.03)	0.13	0.88 (0.82 ,0.94)	0.000	0.96 (0.83 ,1.1)	0.53	0.95 (0.88 ,1.02)	0.16	0.96 (0.83 ,1.1)	0.55	0.95 (0.88 ,1.01)	0.12
20.1-30.0%	0.93 (0.77 ,1.12)	0.46	0.83 (0.75 ,0.91)	0.000	1.08 (0.88 ,1.33)	0.47	0.91 (0.83 ,1)	0.049	1.1 (0.89 ,1.36)	0.36	0.95 (0.86 ,1.04)	0.28
30.1%-	0.84 (0.66 ,1.06)	0.14	0.64 (0.56 ,0.73)	0.000	0.92 (0.71 ,1.19)	0.52	0.73 (0.64 ,0.83)	0.000	1.01 (0.78 ,1.3)	0.96	0.83 (0.73 ,0.95)	0.007
Sex												
Male					reference				reference		reference	
Female					0.72 (0.71 ,0.74)	0.000	0.67 (0.66 ,0.67)	0.000	0.72 (0.71 ,0.74)	0.000	0.67 (0.66 ,0.67)	0.000
Urgency												
Urgent					0.92 (0.87 ,0.97)	0.004	1.09 (1.03 ,1.16)	0.005	0.91 (0.86 ,0.96)	0.001	1.1 (1.03 ,1.16)	0.002
Emergent					reference				reference		reference	
Admission source												
Community					reference				reference		reference	
Hospital					0.91 (0.86 ,0.97)	0.004	1.67 (1.58 ,1.75)	0.000	0.84 (0.8 ,0.89)	0.000	1.33 (1.27 ,1.4)	0.000
SNF/Nursing Home					0.39 (0.35 ,0.43)	0.000	0.45 (0.42 ,0.48)	0.000	0.39 (0.35 ,0.43)	0.000	0.45 (0.42 ,0.48)	0.000
Other					0.9 (0.81 ,1)	0.05	1.32 (1.23 ,1.41)	0.000	0.87 (0.78 ,0.97)	0.01	1.16 (1.08 ,1.25)	0.000
Dual-eligible					0.77 (0.74 ,0.8)	0.000	0.77 (0.75 ,0.79)	0.000	0.78 (0.76 ,0.81)	0.000	0.8 (0.78 ,0.81)	0.000
Body Mass Index												
Underweight					0.59 (0.54 ,0.65)	0.000	0.63 (0.6 ,0.67)	0.000	0.58 (0.53 ,0.64)	0.00	0.61 (0.58 ,0.65)	0.000
Morbid obesity					1.01 (0.95 ,1.08)	0.70	1.03 (1 ,1.05)	0.045	1.01 (0.94 ,1.08)	0.79	1.02 (0.99 ,1.04)	0.12
Myocardial infarction location												
Inferior wall					reference		NA		reference		NA	
Anterior wall					0.99 (0.96 ,1.01)	0.31	NA		0.99 (0.97 ,1.02)	0.66	NA	
Lateral wall					0.69 (0.65 ,0.73)	0.000	NA		0.7 (0.66 ,0.74)	0.000	NA	
Unspecified					0.24 (0.23 ,0.25)	0.000	NA		0.25 (0.24 ,0.26)	0.000	NA	

© 2023 Glance LG et al. *JAMA Network Open.*

	Unadjusted				Patient Charactistics				Patient and Hospital Characteristics			
	STEMI		NSTEMI		STEMI		NSTEMI		STEMI		NSTEMI	
	OR (95% CI)	P	OR (95% CI)	P	OR (95% CI)	P	OR (95% CI)	P	OR (95% CI)	P	OR (95% CI)	P
Congestive heart failure												
Systolic					0.86 (0.83 ,0.89)	0.000	0.8 (0.79 ,0.82)	0.000	0.86 (0.83 ,0.89)	0.000	0.78 (0.76 ,0.79)	0.000
Diastolic					0.84 (0.8 ,0.88)	0.000	0.76 (0.74 ,0.78)	0.000	0.84 (0.8 ,0.88)	0.000	0.75 (0.73 ,0.76)	0.000
Systolic & Diastolic					0.83 (0.78 ,0.87)	0.000	0.81 (0.79 ,0.83)	0.000	0.82 (0.78 ,0.87)	0.000	0.79 (0.77 ,0.82)	0.000
Unspecified					0.63 (0.6 ,0.66)	0.000	0.66 (0.64 ,0.68)	0.000	0.65 (0.62 ,0.68)	0.000	0.69 (0.67 ,0.71)	0.000
Right heart failure					0.83 (0.68 ,1.02)	0.08	0.84 (0.74 ,0.96)	0.000	0.83 (0.68 ,1.03)	0.09	0.85 (0.74 ,0.96)	0.01
Biventricular heart failure					0.73 (0.59 ,0.9)	0.004	0.85 (0.75 ,0.96)	0.008	0.71 (0.57 ,0.88)	0.002	0.8 (0.71 ,0.91)	0.001
End-stage heart failure					0.54 (0.37 ,0.78)	0.001	0.65 (0.55 ,0.76)	0.01	0.54 (0.37 ,0.78)	0.001	0.63 (0.54 ,0.74)	0.000
Complications of AMI												
Ventricular septal defect					0.6 (0.5 ,0.7)	0.000	1 (0.6 ,1.5)	0.84	0.6 (0.5 ,0.7)	0.000	0.9 (0.5 ,1.4)	0.57
LV rupture					0.5 (0.4 ,0.6)	0.000	1.1 (0.7 ,1.7)	0.64	0.5 (0.3 ,0.6)	0.000	1.1 (0.7 ,1.8)	0.72
Papillary muscle rupture					0.9 (0.5 ,1.4)	0.56	1.8 (1.2 ,2.8)	0.005	0.8 (0.5 ,1.3)	0.48	1.7 (1.1 ,2.7)	0.02
Prior procedures												
PCI					0.92 (0.89 ,0.96)	0.000	1.01 (0.99 ,1.03)	0.50	0.92 (0.89 ,0.96)	0.000	0.99 (0.97 ,1)	0.14
CABG					0.45 (0.43 ,0.47)	0.000	0.6 (0.59 ,0.61)	0.000	0.44 (0.42 ,0.46)	0.000	0.58 (0.57 ,0.59)	0.000
Heart valve surgery					0.66 (0.6 ,0.72)	0.000	0.72 (0.7 ,0.75)	0.000	0.65 (0.6 ,0.72)	0.000	0.7 (0.68 ,0.73)	0.000
Dialysis					0.55 (0.51 ,0.6)	0.000	0.86 (0.83 ,0.88)	0.000	0.55 (0.51 ,0.6)	0.000	0.86 (0.83 ,0.88)	0.000
AICD					0.53 (0.48 ,0.58)	0.000	0.68 (0.66 ,0.7)	0.000	0.53 (0.48 ,0.58)	0.000	0.67 (0.65 ,0.68)	0.000
COVID					0.56 (0.45 ,0.71)	0.000	0.43 (0.37 ,0.5)	0.000	0.55 (0.44 ,0.7)	0.000	0.42 (0.36 ,0.49)	0.000
Functional Status												
Wheel-chair					0.51 (0.43 ,0.6)	0.000	0.59 (0.55 ,0.64)	0.000	0.51 (0.43 ,0.6)	0.000	0.57 (0.53 ,0.62)	0.000
Supplemental oxygen					0.67 (0.62 ,0.72)	0.000	0.7 (0.68 ,0.72)	0.000	0.68 (0.63 ,0.73)	0.000	0.71 (0.69 ,0.73)	0.000
Dependent on provider					0.32 (0.26 ,0.38)	0.000	0.35 (0.31 ,0.4)	0.000	0.33 (0.27 ,0.39)	0.000	0.36 (0.32 ,0.41)	0.000
Elixhauser comorbidities												
Cardiac arrhythmias					0.88 (0.86 ,0.9)	0.000	0.8 (0.79 ,0.81)	0.000	0.87 (0.85 ,0.89)	0.000	0.78 (0.77 ,0.79)	0.000
Valvular heart disease					0.89 (0.86 ,0.92)	0.000	0.98 (0.97 ,1)	0.04	0.87 (0.84 ,0.9)	0.000	0.94 (0.93 ,0.96)	0.000
Pulmonary circulation					0.77 (0.73 ,0.81)	0.000	0.84 (0.82 ,0.86)	0.000	0.76 (0.73 ,0.8)	0.000	0.83 (0.81 ,0.85)	0.000
Peripheral vascular disorder					0.85 (0.81 ,0.88)	0.000	1.05 (1.03 ,1.08)	0.000	0.83 (0.8 ,0.87)	0.000	1.03 (1 ,1.05)	0.03
Hypertension, uncomplicated					1.11 (1.08 ,1.14)	0.000	1.18 (1.16 ,1.2)	0.000	1.1 (1.06 ,1.13)	0.000	1.16 (1.14 ,1.18)	0.000
Hypertension, complicated					1.1 (1.06 ,1.14)	0.000	1.2 (1.17 ,1.23)	0.000	1.08 (1.04 ,1.12)	0.000	1.17 (1.14 ,1.19)	0.000
Paralysis					0.65 (0.56 ,0.74)	0.000	0.77 (0.72 ,0.83)	0.000	0.64 (0.56 ,0.74)	0.000	0.75 (0.7 ,0.81)	0.000
Neurologic disorder, other					0.48 (0.47 ,0.5)	0.000	0.58 (0.56 ,0.59)	0.000	0.48 (0.46 ,0.5)	0.000	0.57 (0.56 ,0.58)	0.000
Chronic pulmonary disease					0.86 (0.84 ,0.88)	0.000	0.84 (0.83 ,0.85)	0.000	0.86 (0.84 ,0.89)	0.000	0.84 (0.83 ,0.85)	0.000
Diabetes, uncomplicated					0.9 (0.88 ,0.93)	0.000	0.98 (0.97 ,1)	0.01	0.91 (0.88 ,0.94)	0.000	1 (0.98 ,1.01)	0.83

© 2023 Glance LG et al. *JAMA Network Open.*

	Unadjusted				Patient Charactistics				Patient and Hospital Characteristics			
	STEMI		NSTEMI		STEMI		NSTEMI		STEMI		NSTEMI	
	OR (95% CI)	P	OR (95% CI)	P	OR (95% CI)	P	OR (95% CI)	P	OR (95% CI)	P	OR (95% CI)	P
Diabetes, complicated					0.93 (0.9 ,0.96)	0.000	1.08 (1.07 ,1.1)	0.000	0.92 (0.89 ,0.95)	0.000	1.07 (1.06 ,1.09)	0.000
Hypothyroidism					0.99 (0.96 ,1.01)	0.32	0.99 (0.97 ,1)	0.03	0.98 (0.95 ,1.01)	0.21	0.98 (0.97 ,0.99)	0.001
Renal failure					0.84 (0.81 ,0.87)	0.000	0.81 (0.8 ,0.83)	0.000	0.85 (0.82 ,0.88)	0.000	0.81 (0.79 ,0.82)	0.000
Liver disease					0.73 (0.69 ,0.77)	0.000	0.74 (0.72 ,0.77)	0.000	0.72 (0.68 ,0.76)	0.000	0.73 (0.7 ,0.75)	0.000
Peptic ulcer disease					1.02 (0.87 ,1.18)	0.83	0.85 (0.81 ,0.9)	0.000	1.02 (0.87 ,1.19)	0.80	0.83 (0.79 ,0.88)	0.000
AIDS/HIV					1.2 (0.79 ,1.84)	0.39	1.01 (0.85 ,1.2)	0.92	1.24 (0.81 ,1.92)	0.32	0.98 (0.82 ,1.16)	0.81
Lymphoma					0.76 (0.67 ,0.87)	0.000	0.71 (0.67 ,0.74)	0.000	0.75 (0.66 ,0.85)	0.000	0.68 (0.65 ,0.72)	0.000
Metastatic cancer					0.42 (0.39 ,0.46)	0.000	0.49 (0.47 ,0.51)	0.000	0.42 (0.38 ,0.46)	0.000	0.48 (0.46 ,0.5)	0.000
Solid tumor					0.65 (0.61 ,0.69)	0.000	0.69 (0.67 ,0.71)	0.000	0.65 (0.61 ,0.69)	0.000	0.68 (0.66 ,0.7)	0.000
Rheumatoid arthritis					1.06 (1 ,1.13)	0.05	1 (0.98 ,1.02)	0.95	1.06 (1 ,1.13)	0.048	0.98 (0.95 ,1)	0.07
Coagulopathy					0.88 (0.84 ,0.93)	0.000	1.01 (0.98 ,1.03)	0.52	0.87 (0.83 ,0.92)	0.000	0.98 (0.96 ,1.01)	0.200
Weight loss					0.68 (0.64 ,0.73)	0.000	0.71 (0.68 ,0.74)	0.000	0.69 (0.64 ,0.74)	0.000	0.71 (0.69 ,0.74)	0.000
Fluid and electrolyte disorder					0.64 (0.62 ,0.65)	0.000	0.7 (0.69 ,0.71)	0.000	0.64 (0.62 ,0.66)	0.000	0.7 (0.69 ,0.71)	0.000
Anemia, blood loss					0.77 (0.67 ,0.88)	0.000	0.76 (0.72 ,0.81)	0.000	0.79 (0.69 ,0.91)	0.001	0.76 (0.72 ,0.81)	0.000
Anemia, deficiency					0.89 (0.83 ,0.95)	0.000	0.85 (0.83 ,0.88)	0.000	0.89 (0.83 ,0.95)	0.001	0.84 (0.82 ,0.87)	0.000
Alcohol abuse					0.8 (0.74 ,0.86)	0.000	0.8 (0.78 ,0.83)	0.000	0.79 (0.73 ,0.86)	0.00	0.81 (0.78 ,0.83)	0.000
Drug abuse					0.91 (0.83 ,1)	0.06	0.8 (0.76 ,0.83)	0.000	0.91 (0.83 ,1)	0.06	0.8 (0.76 ,0.83)	0.000
Psychoses					0.47 (0.41 ,0.53)	0.000	0.48 (0.44 ,0.51)	0.000	0.48 (0.41 ,0.55)	0.000	0.49 (0.45 ,0.52)	0.000
Depression					0.84 (0.81 ,0.87)	0.000	0.9 (0.88 ,0.92)	0.000	0.83 (0.8 ,0.87)	0.000	0.88 (0.87 ,0.9)	0.000
Volume of AMI hospitalizations												
<200									reference		reference	
200-499									2.52 (2.23 ,2.85)	0.000	3.14 (2.75 ,3.59)	0.000
500-999									3.07 (2.73 ,3.44)	0.000	4.63 (4.09 ,5.26)	0.000
1000-1999									3.19 (2.83 ,3.59)	0.000	5.37 (4.72 ,6.11)	0.000
2000-									2.96 (2.52 ,3.47)	0.000	5.43 (4.66 ,6.34)	0.000
Proportion of minority patients (AMI)												
<5%									reference		reference	
5.0-9.9%									1 (0.93 ,1.07)	0.92	0.98 (0.9 ,1.05)	0.52
10.0-24.9									1 (0.93 ,1.07)	0.99	1.05 (0.98 ,1.12)	0.20
25.0-49.9%									0.96 (0.88 ,1.05)	0.34	0.97 (0.89 ,1.06)	0.57
50.0%-									0.7 (0.58 ,0.83)	0.000	0.65 (0.55 ,0.76)	0.000
Disproportionate share percentage (DSH)												
0-9.9%									reference		reference	
10.0-24.9%									1.01 (0.91 ,1.12)	0.88	1 (0.88 ,1.13)	0.97
25.0-49.9%									1.05 (0.94 ,1.17)	0.39	1.01 (0.89 ,1.16)	0.84
50.0%-									1.32 (1.13 ,1.55)	0.00	1.18 (0.99 ,1.4)	0.07
Resident-to-bed ratio												
0									reference		reference	
>0-0.10									0.97 (0.91 ,1.04)	0.41	1.05 (0.98 ,1.12)	0.14
0.11-0.20									1.05 (0.97 ,1.13)	0.25	1.09 (1 ,1.18)	0.04
0.21-0.40									0.95 (0.87 ,1.04)	0.29	1.04 (0.95 ,1.15)	0.38
0.41-									0.94 (0.85 ,1.03)	0.19	1.2 (1.1 ,1.32)	0.000

© 2023 Glance LG et al. *JAMA Network Open.*

	Unadjusted				Patient Charactistics				Patient and Hospital Characteristics			
	STEMI		NSTEMI		STEMI		NSTEMI		STEMI		NSTEMI	
	OR (95% CI)	P	OR (95% CI)	P	OR (95% CI)	P	OR (95% CI)	P	OR (95% CI)	P	OR (95% CI)	P
Rurality												
Rural hospital									reference		reference	
Large urban hospital									1.1 (1 ,1.22)	0.047	1.16 (1.04 ,1.29)	0.008
Other urban hospital									1.1 (1 ,1.21)	0.048	1.21 (1.09 ,1.34)	0.000
Time	1.01 (1.01 ,1.01)	0	1.01 (1.01 ,1.01)	0.000	1.01 (1.01 ,1.01)	0.000	1.01 (1.01 ,1.01)	0.000	1.01 (1.01 ,1.01)	0.000	1.01 (1 ,1.01)	0.000
Admission month												
January	reference				reference				reference		reference	
February	0.99 (0.94 ,1.03)	0.56	1 (0.98 ,1.02)	0.76	1.01 (0.96 ,1.06)	0.83	1 (0.98 ,1.02)	0.99	1.01 (0.96 ,1.06)	0.77	1 (0.98 ,1.02)	0.70
March	0.99 (0.95 ,1.04)	0.73	1.02 (1 ,1.05)	0.03	1.01 (0.96 ,1.07)	0.66	1.02 (1 ,1.04)	0.07	1.01 (0.96 ,1.07)	0.59	1.01 (0.99 ,1.03)	0.31
April	1.01 (0.96 ,1.06)	0.79	1.04 (1.01 ,1.06)	0.001	1.01 (0.96 ,1.06)	0.77	1.02 (1 ,1.05)	0.03	1.01 (0.96 ,1.07)	0.69	1.02 (0.99 ,1.04)	0.14
May	0.96 (0.91 ,1.01)	0.09	1.04 (1.02 ,1.07)	0.000	0.97 (0.92 ,1.02)	0.26	1.03 (1.01 ,1.05)	0.005	0.97 (0.92 ,1.02)	0.27	1.02 (1 ,1.04)	0.06
June	0.96 (0.91 ,1.01)	0.09	1.02 (1 ,1.05)	0.03	0.97 (0.92 ,1.02)	0.23	1.01 (0.99 ,1.04)	0.24	0.97 (0.92 ,1.02)	0.29	1 (0.98 ,1.02)	0.98
July	0.96 (0.91 ,1)	0.08	1.01 (0.99 ,1.03)	0.42	0.97 (0.92 ,1.02)	0.30	0.99 (0.97 ,1.02)	0.63	0.98 (0.93 ,1.03)	0.36	0.98 (0.96 ,1.01)	0.16
August	0.94 (0.9 ,0.99)	0.02	1.01 (0.99 ,1.03)	0.40	0.96 (0.91 ,1.01)	0.12	1 (0.98 ,1.02)	0.99	0.96 (0.91 ,1.01)	0.16	0.99 (0.97 ,1.01)	0.44
September	0.9 (0.86 ,0.95)	0.000	0.98 (0.96 ,1)	0.04	0.92 (0.87 ,0.97)	0.002	0.97 (0.95 ,0.99)	0.006	0.92 (0.87 ,0.97)	0.00	0.96 (0.94 ,0.98)	0.000
October	0.96 (0.91 ,1)	0.07	1 (0.98 ,1.02)	0.92	0.96 (0.91 ,1.01)	0.15	0.99 (0.97 ,1.01)	0.38	0.96 (0.91 ,1.02)	0.19	0.99 (0.96 ,1.01)	0.25
November	0.92 (0.88 ,0.97)	0.001	0.99 (0.96 ,1.01)	0.17	0.94 (0.89 ,0.99)	0.02	0.98 (0.96 ,1)	0.05	0.95 (0.9 ,1)	0.04	0.97 (0.95 ,1)	0.02
December	0.91 (0.86 ,0.95)	0.000	0.9 (0.88 ,0.92)	0.00	0.9 (0.85 ,0.95)	0.000	0.87 (0.85 ,0.89)	0.000	0.91 (0.86 ,0.96)	0.000	0.87 (0.85 ,0.89)	0.000
COVID-19 month												
March	reference				reference				reference		reference	
April	0.98 (0.88 ,1.09)	0.67	0.99 (0.94 ,1.03)	0.55	0.94 (0.83 ,1.05)	0.28	0.96 (0.92 ,1.01)	0.14	0.94 (0.83 ,1.05)	0.271	0.97 (0.92 ,1.03)	0.31
May	0.97 (0.85 ,1.1)	0.61	1.12 (1.05 ,1.19)	0.000	0.92 (0.8 ,1.05)	0.23	1.08 (1.01 ,1.15)	0.02	0.93 (0.81 ,1.07)	0.32	1.12 (1.05 ,1.19)	0.00
June	0.99 (0.88 ,1.12)	0.90	1.06 (1 ,1.12)	0.05	1 (0.88 ,1.14)	1.00	1.04 (0.98 ,1.1)	0.18	1.02 (0.9 ,1.16)	0.77	1.08 (1.02 ,1.14)	0.01
July	1 (0.89 ,1.12)	0.97	1.05 (0.99 ,1.1)	0.09	1.01 (0.89 ,1.14)	0.91	1.03 (0.97 ,1.08)	0.34	1.03 (0.91 ,1.16)	0.67	1.08 (1.02 ,1.14)	0.01
August	0.99 (0.87 ,1.12)	0.86	1.02 (0.96 ,1.08)	0.60	0.98 (0.85 ,1.12)	0.73	1.01 (0.94 ,1.07)	0.85	0.99 (0.87 ,1.14)	0.94	1.04 (0.98 ,1.11)	0.18
September	0.99 (0.87 ,1.13)	0.87	1.02 (0.96 ,1.08)	0.61	0.98 (0.85 ,1.13)	0.81	1 (0.94 ,1.07)	0.99	1.01 (0.88 ,1.16)	0.89	1.04 (0.98 ,1.11)	0.20
October	0.97 (0.84 ,1.12)	0.70	0.98 (0.92 ,1.05)	0.64	0.98 (0.84 ,1.15)	0.82	0.96 (0.9 ,1.03)	0.26	1 (0.86 ,1.18)	0.97	1 (0.94 ,1.07)	0.92
November	0.94 (0.82 ,1.09)	0.43	1 (0.93 ,1.07)	0.94	0.94 (0.81 ,1.1)	0.43	0.97 (0.9 ,1.04)	0.35	0.96 (0.82 ,1.12)	0.62	1.01 (0.94 ,1.08)	0.82

© 2023 Glance LG et al. *JAMA Network Open.*

eTable 7. 30-day Mortality for STEMI and NSTEMI with the interaction of race/ethnicity and hospital COVID-19 burden

	Unadjusted				Patient Charactistics				Patient and Hospital Characteristics			
	STEMI		NSTEMI		STEMI		NSTEMI		STEMI		NSTEMI	
	OR (95% CI)	P	OR (95% CI)	P	OR (95% CI)	P	OR (95% CI)	P	OR (95% CI)	P	OR (95% CI)	P
Race												
White	reference				reference				reference		reference	
Black	1.28 (1.23 ,1.34)	0.000	0.98 (0.95 ,1.01)	0.14	0.94 (0.9 ,0.99)	0.027	0.78 (0.76 ,0.81)	0.000	0.92 (0.87 ,0.96)	0.001	0.77 (0.75 ,0.8)	0.000
Hispanic	1.24 (1.18 ,1.31)	0.000	1.06 (1.02 ,1.1)	0.004	1.13 (1.06 ,1.2)	0.000	1.03 (0.98 ,1.08)	0.31	1.06 (1 ,1.12)	0.04	0.97 (0.94 ,1.02)	0.23
Age												
65-69	reference				reference				reference		reference	
70-74	1.16 (1.12 ,1.2)	0.000	0.97 (0.94 ,0.99)	0.02	1.24 (1.2 ,1.29)	0.000	1.17 (1.13 ,1.2)	0.000	1.24 (1.2 ,1.29)	0.000	1.17 (1.13 ,1.2)	0.000
75-79	1.52 (1.47 ,1.57)	0.000	1.28 (1.25 ,1.31)	0.000	1.59 (1.54 ,1.65)	0.000	1.45 (1.42 ,1.49)	0.000	1.59 (1.54 ,1.65)	0.000	1.45 (1.41 ,1.49)	0.000
80-84	2.13 (2.06 ,2.2)	0.000	1.74 (1.7 ,1.79)	0.000	2.19 (2.11 ,2.27)	0.000	1.9 (1.85 ,1.96)	0.000	2.18 (2.1 ,2.26)	0.000	1.9 (1.85 ,1.95)	0.000
85-89	3.17 (3.06 ,3.28)	0.000	2.53 (2.48 ,2.59)	0.000	3.18 (3.06 ,3.31)	0.000	2.65 (2.58 ,2.72)	0.000	3.18 (3.05 ,3.31)	0.000	2.64 (2.57 ,2.71)	0.000
90-94	4.77 (4.59 ,4.96)	0.000	3.71 (3.61 ,3.81)	0.000	4.84 (4.63 ,5.07)	0.000	3.82 (3.71 ,3.94)	0.000	4.84 (4.62 ,5.07)	0.000	3.79 (3.68 ,3.91)	0.000
95-	7.29 (6.87 ,7.73)	0.000	5.5 (5.31 ,5.68)	0.000	7.51 (7.01 ,8.05)	0.000	5.57 (5.36 ,5.79)	0.000	7.47 (6.97 ,8)	0.000	5.52 (5.31 ,5.74)	0.000
Hospital Covid-19 burden												
Before Covid-19	reference				reference				reference		reference	
0.0-2.0%	1 (0.93 ,1.07)	0.96	0.93 (0.89 ,0.97)	0.001	0.98 (0.91 ,1.05)	0.50	0.92 (0.88 ,0.97)	0.001	0.97 (0.9 ,1.05)	0.48	0.92 (0.88 ,0.97)	0.001
2.1-10.0%	1 (0.9 ,1.11)	0.94	0.96 (0.89 ,1.03)	0.242	0.96 (0.85 ,1.08)	0.46	0.94 (0.87 ,1.02)	0.12	0.95 (0.85 ,1.07)	0.44	0.95 (0.88 ,1.03)	0.192
10.1-20.0%	1.15 (1.01 ,1.32)	0.04	1.1 (1 ,1.2)	0.046	1.14 (0.98 ,1.33)	0.09	1.07 (0.97 ,1.18)	0.16	1.12 (0.96 ,1.3)	0.14	1.06 (0.96 ,1.17)	0.23
20.1-30.0%	1 (0.82 ,1.23)	0.97	1.18 (1.02 ,1.36)	0.021	0.92 (0.74 ,1.15)	0.48	1.16 (1 ,1.35)	0.049	0.9 (0.72 ,1.13)	0.38	1.15 (0.99 ,1.33)	0.07
30.1%-	1.17 (0.91 ,1.5)	0.22	1.51 (1.27 ,1.78)	0.000	1.22 (0.94 ,1.58)	0.144	1.53 (1.28 ,1.83)	0.000	1.18 (0.9 ,1.53)	0.23	1.49 (1.25 ,1.77)	0.000
COVID Burden X Race												
0.0-2.0% X Black	1 (0.85 ,1.16)	0.97	1.09 (0.98 ,1.2)	0.10	1.03 (0.87 ,1.22)	0.72	1.08 (0.97 ,1.19)	0.15	1.03 (0.87 ,1.21)	0.75	1.08 (0.97 ,1.2)	0.14
2.1-10% X Black	1.1 (0.92 ,1.31)	0.29	1.18 (1.06 ,1.32)	0.004	1.05 (0.85 ,1.3)	0.65	1.13 (1 ,1.27)	0.049	1.05 (0.85 ,1.3)	0.63	1.12 (0.99 ,1.26)	0.06
10.1-20% X Black	0.85 (0.64 ,1.14)	0.29	1.29 (1.08 ,1.55)	0.005	0.82 (0.59 ,1.13)	0.22	1.23 (1.01 ,1.48)	0.04	0.82 (0.59 ,1.14)	0.23	1.21 (1 ,1.46)	0.06
20.1-30% X Black	0.99 (0.57 ,1.71)	0.96	1.3 (0.96 ,1.77)	0.09	0.8 (0.41 ,1.55)	0.50	1.3 (0.93 ,1.8)	0.12	0.8 (0.41 ,1.55)	0.51	1.26 (0.91 ,1.75)	0.17
30.1%- X Black	0.98 (0.46 ,2.07)	0.96	0.89 (0.57 ,1.4)	0.62	0.82 (0.35 ,1.89)	0.64	0.79 (0.5 ,1.27)	0.33	0.82 (0.35 ,1.91)	0.64	0.8 (0.5 ,1.27)	0.34
0.0-2.0% X Hispanic	0.98 (0.85 ,1.13)	0.80	1.05 (0.95 ,1.16)	0.30	0.93 (0.79 ,1.08)	0.34	1.04 (0.94 ,1.15)	0.44	0.93 (0.8 ,1.09)	0.37	1.03 (0.93 ,1.14)	0.60
2.1-10% X Hispanic	1.07 (0.9 ,1.28)	0.45	1.17 (1.06 ,1.3)	0.003	0.96 (0.78 ,1.17)	0.67	1.05 (0.94 ,1.18)	0.35	0.96 (0.79 ,1.18)	0.73	1.07 (0.96 ,1.19)	0.25
10.1-20% X Hispanic	0.7 (0.52 ,0.94)	0.02	1.1 (0.92 ,1.33)	0.30	0.62 (0.43 ,0.88)	0.008	1 (0.82 ,1.21)	0.96	0.62 (0.43 ,0.89)	0.01	1 (0.82 ,1.22)	0.98
20.1-30% X Hispanic	1.17 (0.75 ,1.84)	0.49	1.25 (0.9 ,1.73)	0.19	1.16 (0.7 ,1.93)	0.56	1.04 (0.73 ,1.5)	0.82	1.18 (0.71 ,1.95)	0.53	1.06 (0.74 ,1.52)	0.76
30.1%- X Hispanic	1.72 (1.12 ,2.63)	0.01	1.24 (0.92 ,1.65)	0.15	1.36 (0.82 ,2.26)	0.24	1.02 (0.74 ,1.4)	0.93	1.32 (0.79 ,2.22)	0.29	1.02 (0.74 ,1.39)	0.92
Sex												
Male					reference				reference		reference	
Female					1.17 (1.15 ,1.2)	0.000	0.93 (0.92 ,0.95)	0.000	1.17 (1.15 ,1.2)	0.000	0.93 (0.92 ,0.95)	0.000
Urgency												
Urgent					0.91 (0.87 ,0.95)	0.000	0.87 (0.84 ,0.9)	0.000	0.91 (0.88 ,0.95)	0.000	0.87 (0.84 ,0.89)	0.000
Emergent					reference				reference		reference	

© 2023 Glance LG et al. *JAMA Network Open.*

	Unadjusted				Patient Charactistics				Patient and Hospital Characteristics			
	STEMI		NSTEMI		STEMI		NSTEMI		STEMI		NSTEMI	
	OR (95% CI)	P	OR (95% CI)	P	OR (95% CI)	P	OR (95% CI)	P	OR (95% CI)	P	OR (95% CI)	P
Admission source												
Community					reference				reference		reference	
Hospital					1.08 (1.03 ,1.13)	0.001	1.08 (1.04 ,1.12)	0.000	1.1 (1.05 ,1.15)	0.000	1.14 (1.1 ,1.18)	0.000
SNF/Nursing Home					2.13 (1.92 ,2.35)	0.000	1.81 (1.72 ,1.9)	0.000	2.15 (1.95 ,2.38)	0.000	1.84 (1.75 ,1.94)	0.000
Other					1.12 (1.02 ,1.22)	0.01	1.23 (1.15 ,1.31)	0.000	1.12 (1.03 ,1.22)	0.01	1.25 (1.18 ,1.33)	0.000
Dual-eligible					1.05 (1.01 ,1.09)	0.000	0.92 (0.89 ,0.94)	0.000	1.03 (1 ,1.07)	0.000	0.91 (0.89 ,0.93)	0.000
Body Mass Index												
Underweight					1.02 (0.92 ,1.13)	0.77	1.18 (1.13 ,1.24)	0.000	1.02 (0.92 ,1.13)	0.74	1.19 (1.13 ,1.24)	0.000
Morbid obesity					1.15 (1.07 ,1.23)	0.000	1 (0.96 ,1.04)	0.948	1.15 (1.07 ,1.23)	0.000	1 (0.96 ,1.04)	1.00
Myocardial infarction location												
Inferior wall					reference		NA		reference		NA	
Anterior wall					1.38 (1.35 ,1.42)	0.000	NA		1.38 (1.35 ,1.42)	0.000	NA	
Lateral wall					1.4 (1.34 ,1.47)	0.000	NA		1.4 (1.34 ,1.46)	0.000	NA	
Unspecified					1.93 (1.87 ,1.99)	0.000	NA		1.9 (1.84 ,1.96)	0.000	NA	
Congestive heart failure												
Systolic					1.29 (1.24 ,1.33)	0.000	2.32 (2.27 ,2.37)	0.000	1.29 (1.24 ,1.34)	0.000	2.34 (2.28 ,2.39)	0.000
Diastolic					0.88 (0.83 ,0.93)	0.000	1.42 (1.38 ,1.46)	0.000	0.88 (0.84 ,0.94)	0.000	1.43 (1.4 ,1.47)	0.000
Systolic & Diastolic					1.15 (1.08 ,1.22)	0.000	2.11 (2.04 ,2.17)	0.000	1.15 (1.08 ,1.22)	0.000	2.12 (2.06 ,2.19)	0.000
Unspecified					2.32 (2.21 ,2.44)	0.000	2.54 (2.47 ,2.62)	0.000	2.3 (2.19 ,2.42)	0.000	2.5 (2.43 ,2.58)	0.000
Right heart failure					4.23 (3.47 ,5.15)	0.000	3.1 (2.64 ,3.64)	0.000	4.24 (3.48 ,5.17)	0.000	3.12 (2.65 ,3.66)	0.000
Biventricular heart failure					4.2 (3.34 ,5.27)	0.000	2.34 (2.01 ,2.73)	0.000	4.25 (3.39 ,5.33)	0.000	2.4 (2.06 ,2.78)	0.000
End-stage heart failure					2.74 (1.89 ,3.98)	0.000	2.91 (2.5 ,3.38)	0.000	2.73 (1.89 ,3.96)	0.000	2.91 (2.5 ,3.38)	0.000
Complications of AMI												
Ventricular septal defect					20.7 (16.3 ,26.3)	0.000	24.3 (14.2 ,41.8)	0.000	20.9 (16.4 ,26.5)	0.000	24.9 (14.5 ,42.7)	0.000
LV rupture					34.8 (25.2 ,48)	0.000	42.1 (22.4 ,79.1)	0.000	35.2 (25.5 ,48.6)	0.000	42.1 (22.4 ,79.1)	0.000
Papillary muscle rupture					10.6 (7.2 ,15.7)	0.000	7.5 (4.5 ,12.5)	0.000	10.7 (7.3 ,15.8)	0.000	7.9 (4.7 ,13)	0.000
Prior procedures												
PCI					0.82 (0.8 ,0.85)	0.000	0.73 (0.72 ,0.75)	0.000	0.82 (0.8 ,0.85)	0.000	0.74 (0.72 ,0.75)	0.000
CABG					1.15 (1.1 ,1.2)	0.000	0.99 (0.97 ,1.01)	0.46	1.15 (1.1 ,1.2)	0.000	0.99 (0.97 ,1.01)	0.33
Heart valve surgery					1.37 (1.25 ,1.51)	0.000	1.02 (0.98 ,1.07)	0.27	1.38 (1.25 ,1.51)	0.000	1.03 (0.99 ,1.08)	0.12
Dialysis					2.59 (2.38 ,2.83)	0.000	2.12 (2.04 ,2.2)	0.000	2.59 (2.37 ,2.82)	0.000	2.12 (2.04 ,2.2)	0.000
AICD					0.99 (0.88 ,1.1)	0.82	0.81 (0.78 ,0.85)	0.000	0.99 (0.88 ,1.1)	0.81	0.81 (0.78 ,0.85)	0.000
COVID					2.64 (2.08 ,3.35)	0.000	2.16 (1.81 ,2.59)	0.000	2.65 (2.08 ,3.36)	0.000	2.16 (1.8 ,2.58)	0.000
Functional Status												
Wheel-chair					1.34 (1.12 ,1.6)	0.001	1.25 (1.16 ,1.36)	0.000	1.35 (1.13 ,1.61)	0.001	1.27 (1.17 ,1.38)	0.000
Supplemental oxygen					1.4 (1.29 ,1.52)	0.000	1.38 (1.34 ,1.43)	0.000	1.4 (1.29 ,1.51)	0.000	1.38 (1.33 ,1.43)	0.000
Dependent on provider					2.15 (1.81 ,2.55)	0.000	2.19 (2.02 ,2.37)	0.000	2.12 (1.78 ,2.51)	0.000	2.14 (1.98 ,2.31)	0.000
Elixhauser comorbidities												
Cardiac arrhythmias					1.57 (1.54 ,1.61)	0.000	1.17 (1.16 ,1.19)	0.000	1.58 (1.54 ,1.61)	0.000	1.18 (1.16 ,1.2)	0.000
Valvular heart disease					0.93 (0.9 ,0.97)	0.000	1.06 (1.04 ,1.08)	0.000	0.94 (0.91 ,0.97)	0.000	1.07 (1.05 ,1.09)	0.000
Pulmonary circulation					1.16 (1.1 ,1.23)	0.000	1.12 (1.09 ,1.14)	0.000	1.16 (1.1 ,1.22)	0.000	1.12 (1.09 ,1.15)	0.000
Peripheral vascular disorder					1.34 (1.3 ,1.4)	0.000	1.16 (1.14 ,1.19)	0.000	1.35 (1.3 ,1.4)	0.000	1.17 (1.15 ,1.2)	0.000

© 2023 Glance LG et al. *JAMA Network Open*.

	Unadjusted				Patient Charactistics				Patient and Hospital Characteristics			
	STEMI		NSTEMI		STEMI		NSTEMI		STEMI		NSTEMI	
	OR (95% CI)	P	OR (95% CI)	P	OR (95% CI)	P	OR (95% CI)	P	OR (95% CI)	P	OR (95% CI)	P
Hypertension, uncomplicated					0.66 (0.64 ,0.68)	0.000	0.66 (0.64 ,0.68)	0.000	0.66 (0.64 ,0.68)	0.000	0.66 (0.65 ,0.68)	0.000
Hypertension, complicated					0.67 (0.64 ,0.69)	0.000	0.69 (0.67 ,0.71)	0.000	0.67 (0.64 ,0.69)	0.000	0.69 (0.67 ,0.71)	0.000
Paralysis					1.59 (1.37 ,1.84)	0.000	1.63 (1.49 ,1.78)	0.000	1.58 (1.37 ,1.83)	0.000	1.63 (1.49 ,1.79)	0.000
Neurologic disorder, other					3.43 (3.3 ,3.56)	0.000	2.22 (2.18 ,2.28)	0.000	3.44 (3.31 ,3.57)	0.000	2.23 (2.18 ,2.28)	0.000
Chronic pulmonary disease					1.12 (1.09 ,1.15)	0.000	1.11 (1.09 ,1.12)	0.000	1.12 (1.08 ,1.15)	0.000	1.1 (1.08 ,1.12)	0.000
Diabetes, uncomplicated					1.21 (1.17 ,1.25)	0.000	1.07 (1.05 ,1.09)	0.000	1.21 (1.17 ,1.24)	0.000	1.06 (1.04 ,1.09)	0.000
Diabetes, complicated					1.29 (1.25 ,1.33)	0.000	1.11 (1.09 ,1.13)	0.000	1.29 (1.25 ,1.33)	0.000	1.11 (1.09 ,1.14)	0.000
Hypothyroidism					0.85 (0.82 ,0.87)	0.000	0.88 (0.87 ,0.9)	0.000	0.85 (0.82 ,0.87)	0.000	0.88 (0.87 ,0.9)	0.000
Renal failure					1.25 (1.2 ,1.29)	0.000	1.37 (1.34 ,1.4)	0.000	1.25 (1.21 ,1.3)	0.000	1.37 (1.35 ,1.4)	0.000
Liver disease					2.67 (2.54 ,2.82)	0.000	2.32 (2.24 ,2.4)	0.000	2.68 (2.55 ,2.83)	0.000	2.34 (2.25 ,2.42)	0.000
Peptic ulcer disease					0.8 (0.67 ,0.95)	0.009	0.85 (0.78 ,0.92)	0.000	0.8 (0.67 ,0.95)	0.01	0.86 (0.79 ,0.93)	0.000
AIDS/HIV					1.1 (0.7 ,1.7)	0.69	1.19 (0.91 ,1.55)	0.21	1.1 (0.7 ,1.71)	0.687	1.23 (0.94 ,1.61)	0.13
Lymphoma					1.33 (1.16 ,1.53)	0.000	1.25 (1.16 ,1.34)	0.000	1.34 (1.16 ,1.54)	0.000	1.26 (1.18 ,1.36)	0.000
Metastatic cancer					2.56 (2.32 ,2.83)	0.000	2.96 (2.81 ,3.11)	0.000	2.57 (2.32 ,2.83)	0.000	2.97 (2.82 ,3.13)	0.000
Solid tumor					1.58 (1.49 ,1.69)	0.000	1.56 (1.51 ,1.63)	0.000	1.59 (1.49 ,1.69)	0.000	1.57 (1.51 ,1.63)	0.000
Rheumatoid arthritis					0.93 (0.87 ,1)	0.048	0.98 (0.95 ,1.02)	0.41	0.94 (0.88 ,1)	0.06	0.99 (0.95 ,1.03)	0.62
Coagulopathy					1.4 (1.33 ,1.48)	0.000	1.3 (1.26 ,1.33)	0.000	1.41 (1.33 ,1.48)	0.000	1.3 (1.26 ,1.34)	0.000
Weight loss					1.35 (1.26 ,1.45)	0.000	1.88 (1.81 ,1.94)	0.000	1.35 (1.26 ,1.45)	0.000	1.88 (1.82 ,1.95)	0.000
Fluid and electrolyte disorder					2.62 (2.54 ,2.69)	0.000	1.94 (1.91 ,1.97)	0.000	2.61 (2.54 ,2.69)	0.000	1.94 (1.91 ,1.97)	0.000
Anemia, blood loss					0.83 (0.71 ,0.98)	0.03	0.91 (0.84 ,0.99)	0.02	0.83 (0.7 ,0.97)	0.019	0.91 (0.84 ,0.99)	0.02
Anemia, deficiency					0.73 (0.68 ,0.79)	0.000	0.85 (0.82 ,0.88)	0.000	0.73 (0.68 ,0.79)	0.000	0.86 (0.83 ,0.89)	0.000
Alcohol abuse					0.99 (0.91 ,1.07)	0.81	0.85 (0.8 ,0.89)	0.000	0.99 (0.91 ,1.07)	0.78	0.85 (0.8 ,0.89)	0.000
Drug abuse					0.74 (0.66 ,0.84)	0.000	0.77 (0.72 ,0.84)	0.000	0.74 (0.66 ,0.84)	0.000	0.78 (0.72 ,0.84)	0.000
Psychoses					1.32 (1.12 ,1.56)	0.001	1.09 (0.99 ,1.2)	0.07	1.32 (1.12 ,1.56)	0.001	1.09 (0.99 ,1.2)	0.07
Depression					0.86 (0.82 ,0.89)	0.000	0.93 (0.91 ,0.95)	0.000	0.86 (0.82 ,0.9)	0.000	0.94 (0.91 ,0.96)	0.000
Volume of AMI hospitalizations												
<200									reference		reference	
200-499									0.83 (0.78 ,0.89)	0.000	0.92 (0.89 ,0.96)	0.000
500-999									0.85 (0.79 ,0.9)	0.000	0.91 (0.88 ,0.95)	0.000
1000-1999									0.84 (0.79 ,0.9)	0.000	0.88 (0.84 ,0.92)	0.000
2000-									0.8 (0.73 ,0.87)	0.000	0.82 (0.77 ,0.88)	0.000
Proportion of minority patients (AMI)												
<5%									reference		reference	
5.0-9.9%									1 (0.95 ,1.05)	0.984	1 (0.97 ,1.04)	0.965
10.0-24.9									1.07 (1.03 ,1.11)	0.001	1.05 (1.02 ,1.09)	0.003
25.0-49.9%									1.04 (0.99 ,1.1)	0.10	1.05 (1.01 ,1.09)	0.024
50.0%-									1.21 (1.1 ,1.32)	0.000	1.24 (1.15 ,1.32)	0.000

© 2023 Glance LG et al. *JAMA Network Open.*

	Unadjusted				Patient Charactistics				Patient and Hospital Characteristics			
	STEMI		NSTEMI		STEMI		NSTEMI		STEMI		NSTEMI	
	OR (95% CI)	P	OR (95% CI)	P	OR (95% CI)	P	OR (95% CI)	P	OR (95% CI)	P	OR (95% CI)	P
Disproportionate share percentage (DSH)												
0-9.9%									reference		reference	
10.0-24.9%									1.03 (0.96 ,1.11)	0.37	1.03 (0.97 ,1.09)	0.41
25.0-49.9%									1.1 (1.02 ,1.18)	0.02	1.07 (1 ,1.14)	0.04
50.0%-									1.08 (0.98 ,1.19)	0.12	0.98 (0.9 ,1.06)	0.57
Resident-to-bed ratio												
0									reference		reference	
>0-0.10									0.96 (0.92 ,1)	0.04	0.97 (0.95 ,1)	0.09
0.11-0.20									0.96 (0.92 ,1.02)	0.17	0.97 (0.94 ,1.01)	0.17
0.21-0.40									0.95 (0.91 ,1)	0.07	0.9 (0.86 ,0.94)	0.000
0.41-									0.97 (0.92 ,1.03)	0.33	0.85 (0.8 ,0.89)	0.000
Rurality												
Rural hospital									reference		reference	
Large urban hospital									0.89 (0.84 ,0.95)	0.000	0.84 (0.8 ,0.88)	0.000
Other urban hospital									0.97 (0.91 ,1.02)	0.25	0.91 (0.87 ,0.96)	0.000
Time	1 (1 ,1)	0.008	1 (1 ,1)	0.000	1 (1 ,1)	0.000	1 (1 ,1)	0.000	1 (1 ,1)	0.000	1 (1 ,1)	0.000
Admission month												
January	reference				reference				reference		reference	
February	1.04 (0.99 ,1.09)	0.12	1.01 (0.97 ,1.04)	0.75	1.03 (0.97 ,1.08)	0.31	1.02 (0.99 ,1.05)	0.23	1.03 (0.97 ,1.08)	0.31	1.02 (0.99 ,1.05)	0.20
March	0.98 (0.93 ,1.03)	0.38	0.98 (0.94 ,1.01)	0.17	0.96 (0.91 ,1.02)	0.21	0.99 (0.95 ,1.02)	0.51	0.96 (0.91 ,1.02)	0.20	0.99 (0.96 ,1.03)	0.60
April	0.98 (0.93 ,1.03)	0.50	0.95 (0.92 ,0.98)	0.002	0.98 (0.93 ,1.04)	0.58	0.97 (0.93 ,1)	0.07	0.98 (0.93 ,1.04)	0.57	0.97 (0.94 ,1.01)	0.10
May	0.98 (0.93 ,1.03)	0.44	0.92 (0.89 ,0.96)	0.000	0.96 (0.9 ,1.02)	0.15	0.94 (0.91 ,0.97)	0.001	0.96 (0.9 ,1.01)	0.14	0.94 (0.91 ,0.98)	0.001
June	0.99 (0.94 ,1.05)	0.77	0.91 (0.88 ,0.94)	0.000	0.99 (0.93 ,1.05)	0.69	0.93 (0.9 ,0.97)	0.000	0.99 (0.93 ,1.05)	0.70	0.93 (0.9 ,0.97)	0.000
July	0.97 (0.92 ,1.02)	0.30	0.89 (0.86 ,0.92)	0.000	0.95 (0.9 ,1.01)	0.10	0.91 (0.88 ,0.95)	0.000	0.95 (0.9 ,1.01)	0.10	0.91 (0.88 ,0.95)	0.000
August	0.96 (0.91 ,1.01)	0.11	0.92 (0.89 ,0.95)	0.000	0.94 (0.89 ,1)	0.04	0.94 (0.9 ,0.97)	0.000	0.94 (0.89 ,1)	0.04	0.94 (0.91 ,0.97)	0.001
September	0.99 (0.94 ,1.04)	0.65	0.93 (0.9 ,0.96)	0.000	0.97 (0.92 ,1.03)	0.35	0.95 (0.92 ,0.98)	0.005	0.97 (0.92 ,1.03)	0.36	0.95 (0.92 ,0.99)	0.009
October	1.01 (0.96 ,1.07)	0.63	0.99 (0.95 ,1.02)	0.44	1.01 (0.95 ,1.07)	0.71	1.01 (0.98 ,1.05)	0.56	1.01 (0.96 ,1.07)	0.69	1.01 (0.98 ,1.05)	0.47
November	1.05 (0.99 ,1.1)	0.09	1.01 (0.97 ,1.04)	0.70	1.02 (0.97 ,1.08)	0.40	1.03 (0.99 ,1.06)	0.15	1.03 (0.97 ,1.08)	0.38	1.03 (0.99 ,1.07)	0.12
December	1.11 (1.05 ,1.17)	0.000	1.02 (0.99 ,1.06)	0.21	1.15 (1.08 ,1.22)	0.000	1.09 (1.05 ,1.13)	0.000	1.15 (1.08 ,1.22)	0.000	1.09 (1.05 ,1.13)	0.000
COVID-19 month												
March	reference				reference				reference		reference	
April	1.15 (1.03 ,1.29)	0.01	1.2 (1.11 ,1.3)	0.000	1.2 (1.06 ,1.35)	0.005	1.25 (1.15 ,1.35)	0.000	1.2 (1.06 ,1.35)	0.005	1.24 (1.15 ,1.35)	0.000
May	1.03 (0.9 ,1.18)	0.68	1.11 (1.01 ,1.22)	0.026	1.04 (0.89 ,1.21)	0.61	1.12 (1.02 ,1.24)	0.02	1.05 (0.9 ,1.22)	0.558	1.12 (1.02 ,1.24)	0.02
June	1.03 (0.9 ,1.16)	0.70	1.12 (1.03 ,1.21)	0.008	1.02 (0.89 ,1.17)	0.77	1.11 (1.02 ,1.21)	0.02	1.02 (0.89 ,1.18)	0.73	1.11 (1.02 ,1.21)	0.02
July	1.07 (0.95 ,1.2)	0.26	1.13 (1.04 ,1.22)	0.004	1.03 (0.9 ,1.17)	0.66	1.16 (1.07 ,1.27)	0.001	1.03 (0.91 ,1.17)	0.64	1.16 (1.06 ,1.26)	0.001
August	1.06 (0.93 ,1.2)	0.38	1.16 (1.06 ,1.27)	0.001	1.1 (0.95 ,1.27)	0.20	1.15 (1.05 ,1.27)	0.003	1.1 (0.96 ,1.28)	0.17	1.15 (1.05 ,1.27)	0.003
September	1.05 (0.92 ,1.2)	0.49	1.15 (1.05 ,1.26)	0.002	1.06 (0.91 ,1.23)	0.46	1.15 (1.05 ,1.27)	0.004	1.06 (0.91 ,1.23)	0.44	1.15 (1.04 ,1.26)	0.007
October	1.02 (0.88 ,1.18)	0.79	1.11 (1.01 ,1.21)	0.04	0.97 (0.82 ,1.14)	0.69	1.13 (1.02 ,1.25)	0.02	0.97 (0.82 ,1.14)	0.70	1.12 (1.02 ,1.24)	0.02
November	1.12 (0.97 ,1.29)	0.12	1.05 (0.95 ,1.16)	0.34	1.08 (0.93 ,1.27)	0.32	1.06 (0.96 ,1.18)	0.25	1.09 (0.93 ,1.27)	0.30	1.06 (0.96 ,1.18)	0.24

© 2023 Glance LG et al. *JAMA Network Open.*

eTable 8. 30-day Readmissions for STEMI and NSTEMI with the interaction of race/ethnicity and hospital COVID-19 burden.

	Unadjusted				Patient Charactistics				Patient and Hospital Characteristics			
	STEMI		NSTEMI		STEMI		NSTEMI		STEMI		NSTEMI	
	OR (95% CI)	P	OR (95% CI)	P	OR (95% CI)	P	OR (95% CI)	P	OR (95% CI)	P	OR (95% CI)	P
Race												
White	reference				reference				reference		reference	
Black	1.28 (1.23 ,1.34)	0.000	1.15 (1.11 ,1.19)	0.000	1.06 (1.01 ,1.11)	0.01	0.98 (0.95 ,1.01)	0.16	1.03 (0.98 ,1.07)	0.27	0.93 (0.91 ,0.95)	0.000
Hispanic	1.21 (1.15 ,1.28)	0.000	1.13 (1.07 ,1.19)	0.000	1.05 (1 ,1.11)	0.06	1 (0.96 ,1.05)	0.86	0.98 (0.94 ,1.03)	0.52	0.94 (0.91 ,0.98)	0.001
Age												
65-69	reference				reference				reference		reference	
70-74	1.08 (1.05 ,1.11)	0.000	0.97 (0.96 ,0.99)	0.00	1.1 (1.07 ,1.13)	0.000	1.06 (1.04 ,1.07)	0.000	1.1 (1.06 ,1.13)	0.000	1.05 (1.03 ,1.07)	0.000
75-79	1.24 (1.21 ,1.28)	0.000	1.05 (1.04 ,1.07)	0.000	1.22 (1.18 ,1.25)	0.000	1.12 (1.1 ,1.14)	0.000	1.21 (1.18 ,1.25)	0.000	1.11 (1.09 ,1.13)	0.000
80-84	1.33 (1.29 ,1.38)	0.000	1.06 (1.04 ,1.08)	0.000	1.26 (1.21 ,1.3)	0.000	1.11 (1.09 ,1.13)	0.000	1.25 (1.21 ,1.29)	0.000	1.08 (1.06 ,1.1)	0.000
85-89	1.33 (1.28 ,1.38)	0.000	1.01 (0.99 ,1.03)	0.51	1.21 (1.16 ,1.26)	0.000	1.04 (1.02 ,1.06)	0.000	1.19 (1.14 ,1.24)	0.000	0.98 (0.96 ,1)	0.09
90-94	1.23 (1.18 ,1.3)	0.000	0.84 (0.82 ,0.86)	0.000	1.09 (1.04 ,1.15)	0.001	0.85 (0.83 ,0.88)	0.000	1.06 (1 ,1.11)	0.03	0.78 (0.76 ,0.8)	0.000
95-	0.88 (0.81 ,0.95)	0.002	0.64 (0.61 ,0.67)	0.000	0.76 (0.7 ,0.83)	0.000	0.65 (0.62 ,0.68)	0.000	0.72 (0.66 ,0.79)	0.000	0.57 (0.54 ,0.6)	0.000
Hospital Covid-19 burden												
Before Covid-19	reference				reference				reference		reference	
0.0-2.0%	0.98 (0.92 ,1.05)	0.61	0.99 (0.96 ,1.02)	0.50	0.98 (0.92 ,1.06)	0.66	0.98 (0.95 ,1.02)	0.36	0.98 (0.91 ,1.05)	0.51	0.98 (0.95 ,1.02)	0.28
2.1-10.0%	1.04 (0.94 ,1.16)	0.47	0.95 (0.89 ,1.01)	0.08	1.02 (0.92 ,1.14)	0.67	0.93 (0.87 ,0.98)	0.01	1.02 (0.92 ,1.14)	0.71	0.97 (0.92 ,1.02)	0.26
10.1-20.0%	1.15 (0.99 ,1.32)	0.06	1.01 (0.94 ,1.1)	0.71	1.14 (0.99 ,1.32)	0.07	0.98 (0.91 ,1.06)	0.65	1.11 (0.96 ,1.28)	0.16	0.97 (0.91 ,1.05)	0.49
20.1-30.0%	1.09 (0.89 ,1.34)	0.42	1.01 (0.9 ,1.13)	0.83	1.08 (0.88 ,1.32)	0.49	0.98 (0.87 ,1.09)	0.68	1.01 (0.82 ,1.24)	0.95	0.94 (0.84 ,1.04)	0.23
30.1%-	1.04 (0.78 ,1.4)	0.77	1.16 (1.01 ,1.35)	0.04	1.04 (0.78 ,1.39)	0.79	1.12 (0.97 ,1.3)	0.13	0.96 (0.71 ,1.28)	0.77	0.98 (0.85 ,1.13)	0.73
COVID Burden X Race												
0.0-2.0% X Black	1.02 (0.88 ,1.18)	0.78	0.97 (0.91 ,1.04)	0.35	1.02 (0.88 ,1.18)	0.79	0.96 (0.9 ,1.03)	0.25	1.02 (0.88 ,1.19)	0.76	0.98 (0.91 ,1.05)	0.54
2.1-10% X Black	1.11 (0.94 ,1.32)	0.22	1.01 (0.94 ,1.09)	0.81	1.08 (0.91 ,1.3)	0.37	1 (0.93 ,1.08)	0.95	1.09 (0.91 ,1.31)	0.34	1.01 (0.94 ,1.1)	0.76
10.1-20% X Black	0.95 (0.72 ,1.25)	0.71	0.96 (0.84 ,1.09)	0.51	0.91 (0.69 ,1.2)	0.52	0.96 (0.84 ,1.09)	0.53	0.9 (0.68 ,1.19)	0.46	0.96 (0.84 ,1.1)	0.57
20.1-30% X Black	1.14 (0.67 ,1.94)	0.64	1.14 (0.9 ,1.44)	0.27	1.07 (0.61 ,1.85)	0.82	1.17 (0.92 ,1.49)	0.19	1.06 (0.61 ,1.85)	0.83	1.16 (0.9 ,1.49)	0.25
30.1%- X Black	1.21 (0.58 ,2.54)	0.61	0.97 (0.69 ,1.38)	0.89	1.19 (0.55 ,2.55)	0.66	0.97 (0.69 ,1.37)	0.85	1.29 (0.6 ,2.79)	0.52	1 (0.7 ,1.43)	1.00
0.0-2.0% X Hispanic	0.93 (0.81 ,1.07)	0.30	0.99 (0.92 ,1.06)	0.70	0.91 (0.79 ,1.05)	0.22	0.99 (0.93 ,1.06)	0.81	0.92 (0.8 ,1.07)	0.29	0.98 (0.92 ,1.05)	0.61
2.1-10% X Hispanic	1.04 (0.86 ,1.26)	0.68	1.02 (0.93 ,1.12)	0.60	1.04 (0.85 ,1.26)	0.71	1.01 (0.93 ,1.11)	0.79	1.06 (0.87 ,1.29)	0.54	1.03 (0.94 ,1.13)	0.49
10.1-20% X Hispanic	0.94 (0.71 ,1.24)	0.67	0.91 (0.79 ,1.05)	0.22	0.93 (0.7 ,1.23)	0.61	0.91 (0.79 ,1.04)	0.18	0.92 (0.69 ,1.23)	0.57	0.91 (0.79 ,1.05)	0.20
20.1-30% X Hispanic	1.32 (0.86 ,2.02)	0.20	1 (0.74 ,1.34)	0.98	1.3 (0.84 ,2.04)	0.24	0.96 (0.72 ,1.28)	0.78	1.36 (0.86 ,2.15)	0.19	0.93 (0.69 ,1.25)	0.63
30.1%- X Hispanic	0.75 (0.42 ,1.34)	0.33	1.16 (0.89 ,1.51)	0.28	0.71 (0.4 ,1.29)	0.26	1.07 (0.82 ,1.4)	0.63	0.74 (0.41 ,1.35)	0.33	1.21 (0.92 ,1.58)	0.17
Sex												
Male					reference				reference		reference	
Female					1.06 (1.03 ,1.08)	0.000	0.98 (0.96 ,0.99)	0.000	1.06 (1.03 ,1.08)	0.000	0.96 (0.95 ,0.97)	0.000
Urgency												
Urgent					0.99 (0.96 ,1.03)	0.78	0.97 (0.93 ,1.02)	0.197	1.01 (0.97 ,1.05)	0.58	0.97 (0.93 ,1.01)	0.10
Emergent					reference				reference		reference	

© 2023 Glance LG et al. *JAMA Network Open.*

	Unadjusted				Patient Charactistics				Patient and Hospital Characteristics			
	STEMI		NSTEMI		STEMI		NSTEMI		STEMI		NSTEMI	
	OR (95% CI)	P	OR (95% CI)	P	OR (95% CI)	P	OR (95% CI)	P	OR (95% CI)	P	OR (95% CI)	P
Admission source												
Community					reference				reference		reference	
Hospital					0.83 (0.8 ,0.87)	0.000	0.67 (0.64 ,0.7)	0.000	0.94 (0.9 ,0.98)	0.003	0.84 (0.81 ,0.87)	0.000
SNF/Nursing Home					0.67 (0.6 ,0.75)	0.000	0.89 (0.85 ,0.93)	0.000	0.66 (0.59 ,0.75)	0.000	0.86 (0.81 ,0.9)	0.000
Other					0.94 (0.86 ,1.03)	0.16	0.77 (0.72 ,0.83)	0.000	0.98 (0.9 ,1.07)	0.69	0.87 (0.81 ,0.93)	0.000
Dual-eligible					1.22 (1.18 ,1.26)	0.000	1.18 (1.16 ,1.2)	0.000	1.21 (1.17 ,1.24)	0.000	1.14 (1.12 ,1.15)	0.000
Body Mass Index												
Underweight					0.9 (0.81 ,1)	0.046	0.92 (0.88 ,0.96)	0.000	0.91 (0.82 ,1.01)	0.07	0.93 (0.89 ,0.97)	0.001
Morbid obesity					1 (0.94 ,1.06)	0.96	1 (0.97 ,1.02)	0.87	1.01 (0.94 ,1.07)	0.87	1.01 (0.98 ,1.03)	0.64
Myocardial infarction location												
Inferior wall					reference		NA		reference		NA	
Anterior wall					1.16 (1.13 ,1.18)	0.000	NA		1.15 (1.12 ,1.18)	0.000	NA	
Lateral wall					1.1 (1.06 ,1.15)	0.000	NA		1.1 (1.05 ,1.15)	0.000	NA	
Unspecified					1.27 (1.23 ,1.32)	0.000	NA		1.2 (1.16 ,1.24)	0.000	NA	
Congestive heart failure												
Systolic					1.51 (1.46 ,1.56)	0.000	1.25 (1.23 ,1.28)	0.000	1.54 (1.49 ,1.59)	0.000	1.28 (1.26 ,1.3)	0.000
Diastolic					1.35 (1.28 ,1.41)	0.000	1.26 (1.24 ,1.29)	0.000	1.35 (1.29 ,1.42)	0.000	1.28 (1.25 ,1.3)	0.000
Systolic & Diastolic					1.51 (1.43 ,1.6)	0.000	1.25 (1.22 ,1.28)	0.000	1.53 (1.45 ,1.61)	0.000	1.27 (1.24 ,1.3)	0.000
Unspecified					1.27 (1.21 ,1.33)	0.000	1.33 (1.3 ,1.36)	0.000	1.24 (1.18 ,1.3)	0.000	1.26 (1.24 ,1.29)	0.000
Right heart failure					1.29 (1.05 ,1.59)	0.01	0.94 (0.82 ,1.08)	0.000	1.31 (1.07 ,1.61)	0.01	0.93 (0.81 ,1.07)	0.29
Biventricular heart failure					0.99 (0.79 ,1.24)	0.94	0.86 (0.76 ,0.97)	0.40	1.03 (0.82 ,1.3)	0.78	0.91 (0.8 ,1.03)	0.14
End-stage heart failure					0.67 (0.45 ,1)	0.049	0.65 (0.55 ,0.77)	0.02	0.67 (0.46 ,1)	0.049	0.66 (0.56 ,0.79)	0.000
Complications of AMI												
Ventricular septal defect					1.9 (1.6 ,2.3)	0.000	1.8 (1.2 ,2.9)	0.01	2 (1.7 ,2.4)	0.000	2 (1.3 ,3.3)	0.003
LV rupture					0.6 (0.4 ,0.8)	0.004	0.6 (0.4 ,1.1)	0.10	0.6 (0.4 ,0.9)	0.005	0.7 (0.4 ,1.2)	0.163
Papillary muscle rupture					1.6 (1.1 ,2.4)	0.013	1.3 (0.8 ,2.2)	0.24	1.7 (1.2 ,2.5)	0.007	1.4 (0.9 ,2.4)	0.148
Prior procedures												
PCI					1.02 (0.99 ,1.05)	0.20	1 (0.98 ,1.01)	0.77	1.02 (0.99 ,1.05)	0.21	1.01 (1 ,1.03)	0.032
CABG					0.99 (0.95 ,1.03)	0.57	0.91 (0.9 ,0.93)	0.000	0.99 (0.95 ,1.03)	0.59	0.92 (0.9 ,0.93)	0.00
Heart valve surgery					1.14 (1.03 ,1.26)	0.01	1.12 (1.08 ,1.15)	0.000	1.15 (1.03 ,1.27)	0.009	1.13 (1.1 ,1.17)	0.00
Dialysis					1.64 (1.52 ,1.78)	0.000	1.58 (1.54 ,1.62)	0.000	1.62 (1.5 ,1.75)	0.000	1.58 (1.54 ,1.62)	0.000
AICD					1.17 (1.06 ,1.28)	0.002	0.98 (0.96 ,1.01)	0.28	1.16 (1.06 ,1.28)	0.002	0.99 (0.96 ,1.02)	0.52
COVID					0.92 (0.7 ,1.21)	0.57	0.97 (0.83 ,1.13)	0.69	0.94 (0.71 ,1.24)	0.66	0.96 (0.83 ,1.13)	0.65
Functional Status												
Wheel-chair					0.92 (0.76 ,1.1)	0.35	0.91 (0.85 ,0.97)	0.005	0.92 (0.77 ,1.11)	0.39	0.92 (0.85 ,0.98)	0.02
Supplemental oxygen					1.07 (0.99 ,1.15)	0.08	1.13 (1.1 ,1.16)	0.000	1.06 (0.98 ,1.14)	0.13	1.12 (1.09 ,1.15)	0.000
Dependent on provider					0.83 (0.69 ,1.01)	0.06	0.89 (0.83 ,0.96)	0.003	0.8 (0.66 ,0.97)	0.02	0.86 (0.8 ,0.93)	0.000
Elixhauser comorbidities												
Cardiac arrhythmias					1.09 (1.07 ,1.12)	0.000	1.05 (1.04 ,1.06)	0.000	1.1 (1.07 ,1.12)	0.000	1.06 (1.05 ,1.07)	0.000
Valvular heart disease					1.18 (1.15 ,1.22)	0.000	1.17 (1.15 ,1.19)	0.000	1.2 (1.17 ,1.24)	0.000	1.21 (1.2 ,1.23)	0.000
Pulmonary circulation					1.09 (1.04 ,1.15)	0.000	1.04 (1.02 ,1.06)	0.000	1.1 (1.04 ,1.15)	0.000	1.04 (1.02 ,1.06)	0.000
Peripheral vascular disorder					1.16 (1.12 ,1.2)	0.000	1.12 (1.1 ,1.14)	0.000	1.17 (1.13 ,1.21)	0.000	1.15 (1.13 ,1.17)	0.000

© 2023 Glance LG et al. *JAMA Network Open.*

	Unadjusted				Patient Charactistics				Patient and Hospital Characteristics			
	STEMI		NSTEMI		STEMI		NSTEMI		STEMI		NSTEMI	
	OR (95% CI)	P	OR (95% CI)	P	OR (95% CI)	P	OR (95% CI)	P	OR (95% CI)	P	OR (95% CI)	P
Hypertension, uncomplicated					1.08 (1.05 ,1.11)	0.000	0.98 (0.96 ,1)	0.04	1.08 (1.05 ,1.12)	0.000	1.01 (0.99 ,1.02)	0.41
Hypertension, complicated					1.14 (1.1 ,1.18)	0.000	1.02 (1 ,1.04)	0.03	1.15 (1.11 ,1.19)	0.000	1.06 (1.04 ,1.08)	0.000
Paralysis					1.56 (1.37 ,1.78)	0.000	1.35 (1.26 ,1.45)	0.000	1.56 (1.36 ,1.78)	0.000	1.38 (1.28 ,1.48)	0.000
Neurologic disorder, other					0.87 (0.83 ,0.91)	0.000	0.93 (0.91 ,0.95)	0.000	0.87 (0.83 ,0.9)	0.000	0.92 (0.9 ,0.94)	0.000
Chronic pulmonary disease					1.18 (1.15 ,1.22)	0.000	1.15 (1.14 ,1.17)	0.000	1.18 (1.15 ,1.21)	0.000	1.15 (1.13 ,1.16)	0.000
Diabetes, uncomplicated					1.14 (1.11 ,1.17)	0.000	1.17 (1.16 ,1.19)	0.000	1.13 (1.1 ,1.17)	0.000	1.16 (1.15 ,1.18)	0.000
Diabetes, complicated					1.29 (1.25 ,1.32)	0.000	1.23 (1.22 ,1.25)	0.000	1.3 (1.26 ,1.34)	0.000	1.26 (1.24 ,1.27)	0.000
Hypothyroidism					1.01 (0.98 ,1.04)	0.69	0.99 (0.97 ,1)	0.06	1.01 (0.98 ,1.04)	0.51	0.99 (0.98 ,1.01)	0.37
Renal failure					1.12 (1.08 ,1.16)	0.000	1.11 (1.09 ,1.13)	0.000	1.12 (1.08 ,1.16)	0.000	1.11 (1.09 ,1.13)	0.000
Liver disease					0.89 (0.84 ,0.94)	0.000	0.97 (0.94 ,1)	0.09	0.89 (0.84 ,0.95)	0.000	0.98 (0.95 ,1.01)	0.27
Peptic ulcer disease					1.11 (0.96 ,1.27)	0.16	1.16 (1.09 ,1.22)	0.000	1.1 (0.96 ,1.27)	0.17	1.18 (1.11 ,1.24)	0.000
AIDS/HIV					1.19 (0.85 ,1.67)	0.30	1.09 (0.92 ,1.3)	0.32	1.19 (0.85 ,1.67)	0.30	1.11 (0.93 ,1.32)	0.25
Lymphoma					1.1 (0.95 ,1.27)	0.18	1.21 (1.15 ,1.28)	0.000	1.12 (0.97 ,1.3)	0.11	1.23 (1.17 ,1.3)	0.000
Metastatic cancer					1.13 (1.02 ,1.25)	0.02	1.03 (0.98 ,1.08)	0.21	1.14 (1.03 ,1.26)	0.01	1.02 (0.97 ,1.07)	0.35
Solid tumor					1.19 (1.11 ,1.27)	0.000	1.13 (1.09 ,1.16)	0.000	1.18 (1.11 ,1.26)	0.000	1.13 (1.1 ,1.16)	0.000
Rheumatoid arthritis					1.09 (1.03 ,1.16)	0.004	1.06 (1.04 ,1.09)	0.000	1.1 (1.04 ,1.17)	0.001	1.08 (1.06 ,1.11)	0.000
Coagulopathy					1.08 (1.03 ,1.14)	0.004	1.02 (0.99 ,1.04)	0.21	1.08 (1.03 ,1.14)	0.003	1.03 (1.01 ,1.06)	0.008
Weight loss					1.13 (1.06 ,1.21)	0.000	1 (0.97 ,1.03)	0.89	1.12 (1.05 ,1.2)	0.001	0.97 (0.94 ,1.01)	0.12
Fluid and electrolyte disorder					1.03 (1.01 ,1.06)	0.020	1.07 (1.05 ,1.08)	0.000	1.02 (0.99 ,1.05)	0.15	1.05 (1.04 ,1.06)	0.000
Anemia, blood loss					1.55 (1.37 ,1.76)	0.000	1.23 (1.17 ,1.3)	0.000	1.53 (1.35 ,1.75)	0.000	1.22 (1.16 ,1.29)	0.000
Anemia, deficiency					1.28 (1.21 ,1.36)	0.000	1.13 (1.1 ,1.16)	0.000	1.29 (1.21 ,1.37)	0.000	1.14 (1.11 ,1.17)	0.000
Alcohol abuse					1.09 (1.01 ,1.17)	0.02	1.06 (1.02 ,1.1)	0.001	1.09 (1.01 ,1.18)	0.02	1.05 (1.01 ,1.09)	0.008
Drug abuse					1.22 (1.11 ,1.34)	0.000	1.1 (1.05 ,1.15)	0.000	1.2 (1.1 ,1.32)	0.000	1.09 (1.04 ,1.14)	0.000
Psychoses					1.34 (1.15 ,1.57)	0.000	1.23 (1.16 ,1.31)	0.000	1.32 (1.13 ,1.54)	0.001	1.18 (1.11 ,1.25)	0.000
Depression					1.08 (1.04 ,1.12)	0.000	1.01 (0.99 ,1.03)	0.22	1.09 (1.05 ,1.13)	0.000	1.02 (1.01 ,1.04)	0.009
Volume of AMI hospitalizations												
<200									reference		reference	
200-499									0.78 (0.73 ,0.83)	0.000	0.59 (0.56 ,0.63)	0.000
500-999									0.65 (0.61 ,0.69)	0.000	0.43 (0.41 ,0.46)	0.000
1000-1999									0.57 (0.53 ,0.61)	0.000	0.35 (0.33 ,0.37)	0.000
2000-									0.53 (0.49 ,0.58)	0.000	0.31 (0.29 ,0.34)	0.000
Proportion of minority patients (AMI)												
<5%									reference		reference	
5.0-9.9%									1.1 (1.04 ,1.16)	0	1.04 (0.98 ,1.1)	0.167
10.0-24.9									1.05 (1 ,1.1)	0.049	1 (0.95 ,1.06)	0.882
25.0-49.9%									1.05 (1 ,1.11)	0.07	1.02 (0.96 ,1.07)	0.585
50.0%-									1.11 (1.01 ,1.21)	0.029	1.09 (1 ,1.19)	0.056

© 2023 Glance LG et al. *JAMA Network Open.*

	Unadjusted				Patient Charactistics				Patient and Hospital Characteristics			
	STEMI		NSTEMI		STEMI		NSTEMI		STEMI		NSTEMI	
	OR (95% CI)	P	OR (95% CI)	P	OR (95% CI)	P	OR (95% CI)	P	OR (95% CI)	P	OR (95% CI)	P
Disproportionate share percentage (DSH)												
0-9.9%									reference		reference	
10.0-24.9%									1.09 (1.01 ,1.17)	0.03	1 (0.91 ,1.09)	0.93
25.0-49.9%									1.06 (0.98 ,1.14)	0.12	0.96 (0.88 ,1.05)	0.39
50.0%-									1.18 (1.07 ,1.3)	0.00	0.97 (0.87 ,1.08)	0.56
Resident-to-bed ratio												
0									reference		reference	
>0-0.10									0.89 (0.85 ,0.93)	0.000	0.93 (0.89 ,0.98)	0.002
0.11-0.20									0.9 (0.85 ,0.95)	0.000	0.94 (0.89 ,1)	0.05
0.21-0.40									0.9 (0.85 ,0.95)	0.000	0.98 (0.91 ,1.05)	0.56
0.41-									0.84 (0.79 ,0.89)	0.000	0.91 (0.85 ,0.97)	0.004
Rurality												
Rural hospital									reference		reference	
Large urban hospital									0.93 (0.87 ,1)	0.048	0.96 (0.9 ,1.03)	0.28
Other urban hospital									0.84 (0.79 ,0.9)	0.00	0.83 (0.78 ,0.89)	0.000
Time	1 (1 ,1)	0.019	1 (1 ,1)	0.000	1 (1 ,1)	0.000	1 (1 ,1)	0.000	1 (1 ,1)	0.002	1 (1 ,1)	0.12
Admission month												
January	reference				reference				reference		reference	
February	0.98 (0.93 ,1.03)	0.41	0.98 (0.96 ,1)	0.11	0.98 (0.93 ,1.02)	0.32	0.99 (0.97 ,1.01)	0.22	0.98 (0.93 ,1.02)	0.33	0.99 (0.97 ,1.01)	0.35
March	0.98 (0.93 ,1.03)	0.36	0.98 (0.96 ,1)	0.08	0.98 (0.93 ,1.03)	0.36	0.98 (0.96 ,1.01)	0.18	0.97 (0.93 ,1.03)	0.31	0.99 (0.97 ,1.01)	0.43
April	0.95 (0.9 ,1)	0.07	0.97 (0.94 ,0.99)	0.004	0.95 (0.9 ,1)	0.06	0.97 (0.95 ,1)	0.04	0.95 (0.9 ,1)	0.05	0.98 (0.96 ,1.01)	0.12
May	0.97 (0.92 ,1.02)	0.18	0.97 (0.95 ,0.99)	0.013	0.96 (0.91 ,1.01)	0.12	0.98 (0.96 ,1.01)	0.13	0.96 (0.91 ,1.01)	0.13	0.99 (0.97 ,1.01)	0.40
June	0.95 (0.91 ,1)	0.07	0.97 (0.94 ,0.99)	0.005	0.96 (0.91 ,1.01)	0.12	0.98 (0.96 ,1.01)	0.14	0.96 (0.91 ,1.01)	0.11	0.99 (0.97 ,1.02)	0.54
July	1 (0.95 ,1.06)	0.88	0.94 (0.92 ,0.97)	0.000	1.01 (0.96 ,1.06)	0.77	0.96 (0.94 ,0.99)	0.003	1.01 (0.95 ,1.06)	0.84	0.97 (0.95 ,1)	0.02
August	0.99 (0.94 ,1.04)	0.76	0.94 (0.92 ,0.97)	0.000	0.99 (0.94 ,1.04)	0.70	0.96 (0.94 ,0.98)	0.001	0.99 (0.94 ,1.04)	0.71	0.97 (0.94 ,0.99)	0.006
September	0.99 (0.94 ,1.04)	0.56	0.95 (0.93 ,0.98)	0.000	0.98 (0.93 ,1.03)	0.50	0.97 (0.95 ,0.99)	0.007	0.98 (0.93 ,1.03)	0.46	0.97 (0.95 ,1)	0.03
October	0.96 (0.92 ,1.01)	0.14	0.96 (0.94 ,0.98)	0.001	0.96 (0.91 ,1.01)	0.12	0.97 (0.95 ,1)	0.02	0.96 (0.91 ,1.01)	0.11	0.97 (0.95 ,1)	0.03
November	0.93 (0.88 ,0.98)	0.01	0.94 (0.92 ,0.96)	0.000	0.92 (0.87 ,0.97)	0.002	0.95 (0.93 ,0.97)	0.000	0.92 (0.87 ,0.97)	0.001	0.95 (0.93 ,0.97)	0.000
December	0.74 (0.7 ,0.79)	0.000	0.78 (0.76 ,0.8)	0.000	0.75 (0.71 ,0.79)	0.000	0.79 (0.77 ,0.81)	0.000	0.74 (0.7 ,0.79)	0.000	0.78 (0.76 ,0.8)	0.000
COVID-19 month												
March	reference				reference				reference		reference	
April	0.83 (0.73 ,0.94)	0.00	0.8 (0.76 ,0.85)	0.000	0.83 (0.73 ,0.94)	0.003	0.81 (0.76 ,0.85)	0.000	0.83 (0.74 ,0.94)	0.004	0.79 (0.75 ,0.84)	0.000
May	0.95 (0.83 ,1.09)	0.48	0.95 (0.88 ,1.01)	0.12	0.96 (0.83 ,1.1)	0.55	0.97 (0.9 ,1.04)	0.35	0.97 (0.84 ,1.12)	0.68	0.95 (0.89 ,1.02)	0.16
June	0.99 (0.88 ,1.11)	0.86	1.01 (0.94 ,1.07)	0.86	0.99 (0.88 ,1.12)	0.88	1.02 (0.96 ,1.08)	0.59	0.99 (0.88 ,1.12)	0.92	1 (0.94 ,1.06)	0.94
July	1.03 (0.92 ,1.16)	0.61	0.99 (0.93 ,1.05)	0.72	1.02 (0.91 ,1.15)	0.72	1 (0.94 ,1.06)	0.94	1.03 (0.91 ,1.16)	0.64	0.97 (0.92 ,1.03)	0.36
August	0.94 (0.82 ,1.07)	0.32	1.01 (0.94 ,1.08)	0.81	0.94 (0.82 ,1.07)	0.35	1.02 (0.95 ,1.09)	0.59	0.95 (0.83 ,1.09)	0.47	1 (0.93 ,1.07)	0.96
September	0.81 (0.71 ,0.93)	0.00	1.03 (0.96 ,1.1)	0.40	0.81 (0.71 ,0.93)	0.003	1.05 (0.98 ,1.12)	0.19	0.81 (0.71 ,0.93)	0.004	1.02 (0.96 ,1.09)	0.53
October	1.03 (0.89 ,1.18)	0.70	1.07 (1 ,1.15)	0.07	1.03 (0.89 ,1.19)	0.71	1.08 (1.01 ,1.16)	0.03	1.04 (0.9 ,1.2)	0.60	1.06 (0.99 ,1.13)	0.11
November	0.92 (0.79 ,1.06)	0.24	0.97 (0.9 ,1.04)	0.35	0.91 (0.79 ,1.05)	0.21	0.99 (0.92 ,1.06)	0.75	0.92 (0.8 ,1.07)	0.28	0.96 (0.9 ,1.03)	0.31

© 2023 Glance LG et al. *JAMA Network Open.*

eTable 9. Non-Home Discharges for STEMI and NSTEMI with the interaction of race/ethnicity and hospital COVID-19 burden

	Unadjusted				Patient Charactistics				Patient and Hospital Characteristics			
	STEMI		NSTEMI		STEMI		NSTEMI		STEMI		NSTEMI	
	OR (95% CI)	P	OR (95% CI)	P	OR (95% CI)	P	OR (95% CI)	P	OR (95% CI)	P	OR (95% CI)	P
Race												
White	reference				reference				reference		reference	
Black	1.41 (1.36 ,1.48)	0.000	1.12 (1.09 ,1.15)	0.000	0.94 (0.9 ,0.99)	0.02	0.78 (0.75 ,0.8)	0.000	0.92 (0.87 ,0.96)	0.001	0.77 (0.75 ,0.79)	0.000
Hispanic	1.08 (1.03 ,1.14)	0.003	0.8 (0.75 ,0.86)	0.000	0.86 (0.81 ,0.9)	0.000	0.65 (0.62 ,0.68)	0.000	0.84 (0.8 ,0.88)	0.000	0.64 (0.62 ,0.67)	0.000
Age												
65-69	reference				reference				reference		reference	
70-74	1.21 (1.18 ,1.25)	0.000	1.1 (1.08 ,1.12)	0.000	1.33 (1.29 ,1.38)	0.000	1.31 (1.29 ,1.34)	0.000	1.33 (1.29 ,1.38)	0.000	1.32 (1.29 ,1.34)	0.000
75-79	1.73 (1.69 ,1.78)	0.000	1.49 (1.46 ,1.52)	0.000	1.89 (1.83 ,1.95)	0.000	1.76 (1.73 ,1.8)	0.000	1.89 (1.83 ,1.95)	0.000	1.77 (1.74 ,1.8)	0.000
80-84	2.62 (2.55 ,2.7)	0.000	2.08 (2.04 ,2.12)	0.000	2.83 (2.74 ,2.93)	0.000	2.44 (2.39 ,2.49)	0.000	2.84 (2.75 ,2.94)	0.000	2.45 (2.4 ,2.51)	0.000
85-89	4.24 (4.11 ,4.38)	0.000	3.01 (2.94 ,3.07)	0.000	4.47 (4.3 ,4.64)	0.000	3.4 (3.33 ,3.48)	0.000	4.49 (4.32 ,4.66)	0.000	3.45 (3.37 ,3.53)	0.000
90-94	6.75 (6.49 ,7.02)	0.000	4.38 (4.28 ,4.5)	0.000	7.04 (6.71 ,7.37)	0.000	4.84 (4.7 ,4.98)	0.000	7.11 (6.78 ,7.45)	0.000	4.93 (4.79 ,5.07)	0.000
95-	10.61 (9.98 ,11.28)	0.000	6.89 (6.67 ,7.12)	0.000	10.95 (10.2 ,11.76)	0.000	7.32 (7.05 ,7.6)	0.000	11.13 (10.36 ,11.95)	0.000	7.51 (7.23 ,7.8)	0.000
Hospital Covid-19 burden												
Before Covid-19	reference				reference				reference		reference	
0.0-2.0%	0.99 (0.94 ,1.05)	0.80	1.01 (0.97 ,1.04)	0.67	0.98 (0.91 ,1.04)	0.50	1 (0.96 ,1.03)	0.82	0.98 (0.92 ,1.05)	0.53	1 (0.96 ,1.03)	0.92
2.1-10.0%	0.97 (0.88 ,1.07)	0.57	1.02 (0.97 ,1.08)	0.46	0.93 (0.83 ,1.04)	0.21	0.99 (0.93 ,1.05)	0.78	0.91 (0.81 ,1.02)	0.10	0.96 (0.91 ,1.02)	0.22
10.1-20.0%	1.04 (0.92 ,1.18)	0.49	1.05 (0.97 ,1.13)	0.23	1.03 (0.89 ,1.19)	0.68	1.01 (0.93 ,1.1)	0.76	1.01 (0.87 ,1.16)	0.91	0.99 (0.91 ,1.08)	0.82
20.1-30.0%	0.85 (0.7 ,1.03)	0.10	1.04 (0.93 ,1.17)	0.46	0.75 (0.59 ,0.94)	0.01	1 (0.88 ,1.12)	0.94	0.74 (0.59 ,0.93)	0.01	0.98 (0.87 ,1.11)	0.77
30.1%-	0.89 (0.69 ,1.14)	0.35	1.31 (1.13 ,1.51)	0.000	0.89 (0.68 ,1.18)	0.43	1.32 (1.13 ,1.55)	0.001	0.89 (0.67 ,1.17)	0.39	1.34 (1.14 ,1.57)	0.000
COVID Burden X Race												
0.0-2.0% X Black	1.12 (0.98 ,1.28)	0.09	1.03 (0.95 ,1.11)	0.48	1.16 (1 ,1.35)	0.05	1.02 (0.94 ,1.11)	0.56	1.16 (0.99 ,1.34)	0.06	1.02 (0.94 ,1.11)	0.65
2.1-10% X Black	1.15 (0.97 ,1.35)	0.10	1.07 (0.98 ,1.17)	0.11	1.08 (0.88 ,1.32)	0.48	1.03 (0.93 ,1.13)	0.57	1.08 (0.88 ,1.33)	0.44	1.03 (0.94 ,1.14)	0.51
10.1-20% X Black	0.99 (0.75 ,1.3)	0.93	1.2 (1.03 ,1.39)	0.02	0.95 (0.68 ,1.32)	0.76	1.13 (0.97 ,1.33)	0.12	0.96 (0.69 ,1.34)	0.82	1.14 (0.98 ,1.34)	0.10
20.1-30% X Black	1.03 (0.65 ,1.64)	0.90	1.26 (0.97 ,1.64)	0.09	0.78 (0.48 ,1.28)	0.32	1.29 (0.96 ,1.72)	0.09	0.78 (0.48 ,1.29)	0.34	1.3 (0.97 ,1.74)	0.08
30.1%- X Black	1.54 (0.78 ,3.06)	0.21	0.99 (0.68 ,1.45)	0.97	1.47 (0.69 ,3.16)	0.32	0.92 (0.61 ,1.37)	0.67	1.45 (0.68 ,3.09)	0.34	0.92 (0.62 ,1.38)	0.70
0.0-2.0% X Hispanic	1.02 (0.89 ,1.18)	0.75	1.01 (0.93 ,1.1)	0.82	0.95 (0.8 ,1.11)	0.50	1.05 (0.95 ,1.15)	0.34	0.94 (0.8 ,1.11)	0.46	1.04 (0.95 ,1.14)	0.36
2.1-10% X Hispanic	1.17 (0.99 ,1.39)	0.07	1.26 (1.13 ,1.4)	0.000	1.04 (0.85 ,1.27)	0.71	1.14 (1.02 ,1.28)	0.02	1.04 (0.85 ,1.27)	0.70	1.15 (1.03 ,1.29)	0.02
10.1-20% X Hispanic	0.96 (0.76 ,1.21)	0.72	1.33 (1.12 ,1.58)	0.001	0.86 (0.64 ,1.15)	0.31	1.21 (1.02 ,1.44)	0.03	0.86 (0.64 ,1.16)	0.33	1.21 (1.01 ,1.44)	0.04
20.1-30% X Hispanic	1.27 (0.81 ,2)	0.30	1.23 (0.93 ,1.63)	0.14	1.21 (0.69 ,2.11)	0.51	0.99 (0.73 ,1.36)	0.97	1.22 (0.7 ,2.13)	0.49	1.01 (0.74 ,1.38)	0.96
30.1%- X Hispanic	1.8 (1.06 ,3.04)	0.03	1.36 (1 ,1.84)	0.05	1.31 (0.73 ,2.34)	0.37	1.04 (0.75 ,1.45)	0.81	1.28 (0.71 ,2.3)	0.41	1.02 (0.73 ,1.42)	0.92
Sex												
Male					reference				reference		reference	
Female					1.31 (1.28 ,1.34)	0.000	1.08 (1.06 ,1.09)	0.000	1.32 (1.29 ,1.34)	0.000	1.08 (1.06 ,1.09)	0.000
Urgency												
Urgent					0.96 (0.92 ,1.01)	0.09	0.92 (0.88 ,0.95)	0.000	0.96 (0.92 ,1.01)	0.09	0.92 (0.88 ,0.95)	0.000
Emergent					reference				reference		reference	

© 2023 Glance LG et al. *JAMA Network Open.*

	Unadjusted				Patient Charactistics				Patient and Hospital Characteristics			
	STEMI		NSTEMI		STEMI		NSTEMI		STEMI		NSTEMI	
	OR (95% CI)	P	OR (95% CI)	P	OR (95% CI)	P	OR (95% CI)	P	OR (95% CI)	P	OR (95% CI)	P
Admission source												
Community					reference				reference		reference	
Hospital					1.18 (1.13 ,1.24)	0.000	1.31 (1.26 ,1.37)	0.000	1.16 (1.11 ,1.22)	0.000	1.29 (1.24 ,1.34)	0.000
SNF/Nursing Home					5.08 (4.43 ,5.82)	0.000	4.95 (4.65 ,5.26)	0.000	5.11 (4.44 ,5.87)	0.000	5.01 (4.71 ,5.33)	0.000
Other					1.25 (1.14 ,1.38)	0.000	1.47 (1.37 ,1.57)	0.000	1.24 (1.13 ,1.36)	0.000	1.45 (1.36 ,1.55)	0.000
Dual-eligible					1.54 (1.49 ,1.58)	0.000	1.64 (1.61 ,1.67)	0.000	1.53 (1.49 ,1.58)	0.000	1.65 (1.62 ,1.69)	0.000
Body Mass Index												
Underweight					0.95 (0.86 ,1.06)	0.38	1.1 (1.05 ,1.15)	0.000	0.95 (0.86 ,1.06)	0.36	1.1 (1.06 ,1.15)	0.000
Morbid obesity					1.51 (1.43 ,1.6)	0.000	1.42 (1.38 ,1.46)	0.000	1.52 (1.43 ,1.61)	0.000	1.43 (1.39 ,1.47)	0.000
Myocardial infarction location												
Inferior wall					reference		NA		reference		NA	
Anterior wall					1.2 (1.18 ,1.23)	0.000	NA		1.21 (1.18 ,1.24)	0.000	NA	
Lateral wall					1.23 (1.18 ,1.28)	0.000	NA		1.23 (1.19 ,1.28)	0.000	NA	
Unspecified					1.62 (1.57 ,1.67)	0.000	NA		1.63 (1.58 ,1.69)	0.000	NA	
Congestive heart failure												
Systolic					1.93 (1.86 ,1.99)	0.000	2.13 (2.09 ,2.17)	0.000	1.92 (1.86 ,1.99)	0.000	2.13 (2.09 ,2.17)	0.000
Diastolic					1.42 (1.35 ,1.49)	0.000	1.61 (1.58 ,1.65)	0.000	1.42 (1.35 ,1.49)	0.000	1.62 (1.59 ,1.65)	0.000
Systolic & Diastolic					1.91 (1.8 ,2.02)	0.000	2.13 (2.08 ,2.18)	0.000	1.91 (1.8 ,2.02)	0.000	2.13 (2.08 ,2.18)	0.000
Unspecified					2.27 (2.16 ,2.37)	0.000	1.82 (1.78 ,1.87)	0.000	2.27 (2.17 ,2.38)	0.000	1.84 (1.79 ,1.89)	0.000
Right heart failure					3.92 (3.17 ,4.86)	0.000	2.7 (2.34 ,3.12)	0.000	3.91 (3.16 ,4.84)	0.000	2.73 (2.36 ,3.15)	0.000
Biventricular heart failure					3.68 (2.95 ,4.59)	0.000	1.82 (1.59 ,2.08)	0.000	3.65 (2.92 ,4.55)	0.000	1.82 (1.6 ,2.08)	0.000
End-stage heart failure					2.88 (1.93 ,4.31)	0.000	2.91 (2.5 ,3.4)	0.000	2.88 (1.93 ,4.31)	0.000	2.91 (2.49 ,3.4)	0.000
Complications of AMI												
Ventricular septal defect					7.9 (6.4 ,9.8)	0.000	4.6 (2.7 ,7.8)	0.000	7.8 (6.3 ,9.7)	0.000	4.5 (2.7 ,7.6)	0.000
LV rupture					38.1 (25.8 ,56.4)	0.000	25.2 (13.5 ,47)	0.000	38.4 (26 ,56.9)	0.000	25.2 (13.5 ,47.1)	0.000
Papillary muscle rupture					9.4 (6.1 ,14.7)	0.000	9.9 (5.6 ,17.4)	0.000	9.3 (6 ,14.5)	0.000	9.9 (5.7 ,17.4)	0.000
Prior procedures												
PCI					0.8 (0.77 ,0.82)	0.000	0.66 (0.65 ,0.67)	0.000	0.8 (0.77 ,0.82)	0.000	0.66 (0.65 ,0.67)	0.000
CABG					0.92 (0.89 ,0.96)	0.000	0.73 (0.72 ,0.75)	0.000	0.92 (0.89 ,0.96)	0.000	0.73 (0.72 ,0.75)	0.000
Heart valve surgery					1.05 (0.95 ,1.15)	0.33	0.85 (0.82 ,0.89)	0.000	1.05 (0.95 ,1.15)	0.35	0.85 (0.82 ,0.88)	0.000
Dialysis					2.23 (2.05 ,2.43)	0.000	1.77 (1.71 ,1.83)	0.000	2.24 (2.06 ,2.44)	0.000	1.77 (1.71 ,1.83)	0.000
AICD					0.74 (0.67 ,0.82)	0.000	0.66 (0.63 ,0.68)	0.000	0.74 (0.67 ,0.82)	0.000	0.66 (0.63 ,0.68)	0.000
COVID					2.75 (2.18 ,3.48)	0.000	2.31 (1.98 ,2.69)	0.000	2.75 (2.17 ,3.49)	0.000	2.32 (1.99 ,2.71)	0.000
Functional Status												
Wheel-chair					1.73 (1.45 ,2.07)	0.000	1.73 (1.61 ,1.85)	0.000	1.73 (1.45 ,2.07)	0.000	1.72 (1.6 ,1.84)	0.000
Supplemental oxygen					1.29 (1.2 ,1.4)	0.000	1.08 (1.05 ,1.11)	0.000	1.3 (1.2 ,1.4)	0.000	1.09 (1.06 ,1.12)	0.000
Dependent on provider					2.01 (1.68 ,2.41)	0.000	2.18 (2.01 ,2.36)	0.000	2.01 (1.67 ,2.41)	0.000	2.19 (2.02 ,2.38)	0.000
Elixhauser comorbidities												
Cardiac arrhythmias					1.62 (1.58 ,1.65)	0.000	1.17 (1.15 ,1.18)	0.000	1.62 (1.58 ,1.65)	0.000	1.17 (1.15 ,1.18)	0.000
Valvular heart disease					0.95 (0.92 ,0.99)	0.005	1.04 (1.02 ,1.05)	0.000	0.95 (0.92 ,0.98)	0.003	1.03 (1.02 ,1.05)	0.000
Pulmonary circulation					1.17 (1.11 ,1.23)	0.000	1.14 (1.11 ,1.16)	0.000	1.17 (1.11 ,1.23)	0.000	1.14 (1.11 ,1.16)	0.000
Peripheral vascular disorder					1.32 (1.27 ,1.37)	0.000	1.1 (1.08 ,1.13)	0.000	1.32 (1.27 ,1.37)	0.000	1.1 (1.08 ,1.12)	0.000

© 2023 Glance LG et al. *JAMA Network Open.*

	Unadjusted				Patient Charactistics				Patient and Hospital Characteristics			
	STEMI		NSTEMI		STEMI		NSTEMI		STEMI		NSTEMI	
	OR (95% CI)	P	OR (95% CI)	P	OR (95% CI)	P	OR (95% CI)	P	OR (95% CI)	P	OR (95% CI)	P
Hypertension, uncomplicated					0.74 (0.72 ,0.76)	0.000	0.78 (0.77 ,0.8)	0.000	0.74 (0.72 ,0.76)	0.000	0.78 (0.76 ,0.79)	0.000
Hypertension, complicated					0.79 (0.76 ,0.81)	0.000	0.84 (0.82 ,0.86)	0.000	0.78 (0.75 ,0.81)	0.000	0.83 (0.81 ,0.85)	0.000
Paralysis					3.73 (3.19 ,4.37)	0.000	2.95 (2.74 ,3.19)	0.000	3.74 (3.19 ,4.38)	0.000	2.95 (2.73 ,3.19)	0.000
Neurologic disorder, other					4.84 (4.66 ,5.03)	0.000	2.93 (2.87 ,2.99)	0.000	4.85 (4.67 ,5.04)	0.000	2.93 (2.88 ,2.99)	0.000
Chronic pulmonary disease					1.11 (1.08 ,1.14)	0.000	1.03 (1.02 ,1.05)	0.000	1.11 (1.08 ,1.14)	0.000	1.03 (1.02 ,1.05)	0.000
Diabetes, uncomplicated					1.2 (1.17 ,1.23)	0.000	1.04 (1.03 ,1.06)	0.000	1.2 (1.17 ,1.24)	0.000	1.04 (1.03 ,1.06)	0.000
Diabetes, complicated					1.43 (1.39 ,1.47)	0.000	1.3 (1.28 ,1.32)	0.000	1.42 (1.38 ,1.47)	0.000	1.3 (1.28 ,1.32)	0.000
Hypothyroidism					0.93 (0.91 ,0.96)	0.000	0.97 (0.96 ,0.98)	0.000	0.93 (0.91 ,0.96)	0.000	0.97 (0.96 ,0.98)	0.000
Renal failure					1.25 (1.21 ,1.29)	0.000	1.17 (1.15 ,1.19)	0.000	1.25 (1.21 ,1.29)	0.000	1.18 (1.16 ,1.2)	0.000
Liver disease					2.55 (2.42 ,2.69)	0.000	1.69 (1.64 ,1.75)	0.000	2.55 (2.42 ,2.69)	0.000	1.69 (1.64 ,1.74)	0.000
Peptic ulcer disease					1.08 (0.94 ,1.24)	0.29	1.12 (1.06 ,1.19)	0.000	1.08 (0.94 ,1.24)	0.29	1.12 (1.05 ,1.19)	0.000
AIDS/HIV					0.93 (0.62 ,1.37)	0.70	0.96 (0.76 ,1.2)	0.71	0.92 (0.62 ,1.36)	0.68	0.96 (0.76 ,1.2)	0.70
Lymphoma					1.19 (1.04 ,1.36)	0.01	0.99 (0.93 ,1.05)	0.65	1.18 (1.03 ,1.35)	0.02	0.98 (0.93 ,1.05)	0.62
Metastatic cancer					1.93 (1.75 ,2.12)	0.000	1.86 (1.77 ,1.95)	0.000	1.93 (1.75 ,2.12)	0.000	1.87 (1.78 ,1.96)	0.000
Solid tumor					1.35 (1.27 ,1.44)	0.000	1.2 (1.16 ,1.24)	0.000	1.35 (1.27 ,1.44)	0.000	1.2 (1.17 ,1.24)	0.000
Rheumatoid arthritis					0.95 (0.9 ,1.01)	0.10	1.04 (1.01 ,1.07)	0.007	0.95 (0.9 ,1.01)	0.10	1.04 (1.01 ,1.07)	0.01
Coagulopathy					1.69 (1.61 ,1.78)	0.000	1.41 (1.38 ,1.44)	0.000	1.69 (1.6 ,1.77)	0.000	1.4 (1.37 ,1.44)	0.000
Weight loss					2.35 (2.19 ,2.53)	0.000	2.5 (2.41 ,2.58)	0.000	2.36 (2.19 ,2.54)	0.000	2.51 (2.43 ,2.6)	0.000
Fluid and electrolyte disorder					2.58 (2.51 ,2.65)	0.000	1.86 (1.84 ,1.89)	0.000	2.58 (2.51 ,2.65)	0.000	1.86 (1.84 ,1.89)	0.000
Anemia, blood loss					1.26 (1.09 ,1.46)	0.002	1.21 (1.15 ,1.29)	0.000	1.26 (1.09 ,1.47)	0.002	1.22 (1.15 ,1.29)	0.000
Anemia, deficiency					0.97 (0.9 ,1.03)	0.34	1.05 (1.02 ,1.08)	0.000	0.97 (0.9 ,1.03)	0.33	1.05 (1.03 ,1.08)	0.000
Alcohol abuse					1.17 (1.09 ,1.26)	0.00	1.19 (1.15 ,1.24)	0.000	1.16 (1.08 ,1.26)	0.00	1.2 (1.15 ,1.24)	0.000
Drug abuse					0.93 (0.85 ,1.03)	0.16	0.9 (0.85 ,0.95)	0.000	0.93 (0.85 ,1.03)	0.17	0.9 (0.85 ,0.95)	0.000
Psychoses					2.36 (2.01 ,2.77)	0.000	2.32 (2.17 ,2.49)	0.000	2.37 (2.02 ,2.77)	0.000	2.34 (2.19 ,2.51)	0.000
Depression					1.2 (1.16 ,1.25)	0.000	1.28 (1.26 ,1.31)	0.000	1.2 (1.16 ,1.25)	0.000	1.28 (1.26 ,1.31)	0.000
Volume of AMI hospitalizations												
<200									reference		reference	
200-499									0.98 (0.91 ,1.05)	0.55	1.04 (1 ,1.09)	0.07
500-999									1.09 (1.02 ,1.16)	0.01	1.18 (1.13 ,1.23)	0.000
1000-1999									1.12 (1.05 ,1.2)	0.001	1.23 (1.17 ,1.29)	0.000
2000-									1.07 (0.98 ,1.18)	0.14	1.13 (1.04 ,1.23)	0.003
Proportion of minority patients (AMI)												
<5%									reference		reference	
5.0-9.9%									1.05 (0.99 ,1.11)	0.09	1.01 (0.97 ,1.06)	0.62
10.0-24.9									1.17 (1.11 ,1.23)	0.000	1.1 (1.05 ,1.15)	0.000
25.0-49.9%									1.05 (0.98 ,1.11)	0.14	1.02 (0.97 ,1.08)	0.40
50.0%-									1.13 (1.03 ,1.24)	0.007	1.03 (0.94 ,1.13)	0.47

© 2023 Glance LG et al. *JAMA Network Open.*

	Unadjusted				Patient Charactistics				Patient and Hospital Characteristics			
	STEMI		NSTEMI		STEMI		NSTEMI		STEMI		NSTEMI	
	OR (95% CI)	P	OR (95% CI)	P	OR (95% CI)	P	OR (95% CI)	P	OR (95% CI)	P	OR (95% CI)	P
Disproportionate share percentage (DSH)												
0-9.9%									reference		reference	
10.0-24.9%									1.02 (0.94 ,1.1)	0.68	1.13 (1.05 ,1.21)	0.00
25.0-49.9%									1.03 (0.95 ,1.12)	0.49	1.14 (1.06 ,1.23)	0.00
50.0%-									1.03 (0.92 ,1.15)	0.57	1.15 (1.04 ,1.27)	0.01
Resident-to-bed ratio												
0									reference		reference	
>0-0.10									1 (0.96 ,1.05)	0.88	1 (0.96 ,1.04)	0.90
0.11-0.20									1.01 (0.95 ,1.07)	0.87	1.03 (0.98 ,1.08)	0.29
0.21-0.40									1 (0.94 ,1.06)	0.96	0.99 (0.93 ,1.05)	0.71
0.41-									1.07 (1 ,1.14)	0.04	0.95 (0.9 ,1)	0.07
Rurality												
Rural hospital									reference		reference	
Large urban hospital									0.99 (0.92 ,1.07)	0.85	1.08 (1.02 ,1.15)	0.01
Other urban hospital									1.05 (0.98 ,1.13)	0.14	1.08 (1.01 ,1.14)	0.02
Time	1 (1 ,1)	0	0.99 (0.99 ,1)	0.000	0.99 (0.99 ,0.99)	0.000	0.99 (0.99 ,0.99)	0.000	0.99 (0.99 ,0.99)	0.000	0.99 (0.99 ,0.99)	0.000
Admission month												
January	reference				reference				reference		reference	
February	1.05 (1.01 ,1.09)	0.02	0.97 (0.95 ,0.99)	0.01	1.05 (1 ,1.1)	0.05	0.98 (0.95 ,1)	0.09	1.05 (1 ,1.1)	0.05	0.98 (0.95 ,1)	0.08
March	1.01 (0.97 ,1.06)	0.51	0.97 (0.95 ,0.99)	0.01	1 (0.95 ,1.05)	0.99	0.98 (0.96 ,1.01)	0.16	1 (0.95 ,1.05)	0.99	0.98 (0.96 ,1.01)	0.15
April	1 (0.95 ,1.04)	0.83	0.94 (0.92 ,0.97)	0.000	1 (0.95 ,1.05)	0.85	0.96 (0.93 ,0.98)	0.001	1 (0.95 ,1.05)	0.88	0.96 (0.93 ,0.98)	0.002
May	1.01 (0.97 ,1.06)	0.54	0.92 (0.9 ,0.95)	0.000	0.99 (0.94 ,1.04)	0.78	0.93 (0.91 ,0.96)	0.000	0.99 (0.95 ,1.04)	0.80	0.93 (0.91 ,0.96)	0.000
June	1.01 (0.96 ,1.05)	0.76	0.92 (0.9 ,0.94)	0.000	1 (0.95 ,1.05)	0.95	0.93 (0.9 ,0.95)	0.000	1 (0.95 ,1.06)	0.88	0.93 (0.9 ,0.95)	0.000
July	1.01 (0.97 ,1.05)	0.68	0.92 (0.9 ,0.94)	0.000	0.99 (0.94 ,1.04)	0.78	0.94 (0.91 ,0.96)	0.000	0.99 (0.95 ,1.05)	0.84	0.94 (0.91 ,0.96)	0.000
August	0.96 (0.92 ,1.01)	0.12	0.94 (0.92 ,0.96)	0.000	0.94 (0.89 ,0.99)	0.02	0.96 (0.93 ,0.98)	0.001	0.94 (0.89 ,0.99)	0.02	0.96 (0.93 ,0.98)	0.001
September	1 (0.96 ,1.05)	0.97	0.95 (0.93 ,0.97)	0.000	0.98 (0.93 ,1.03)	0.40	0.96 (0.94 ,0.99)	0.007	0.98 (0.93 ,1.03)	0.45	0.96 (0.94 ,0.99)	0.008
October	1.06 (1.02 ,1.11)	0.01	0.97 (0.95 ,0.99)	0.02	1.07 (1.02 ,1.12)	0.01	0.99 (0.97 ,1.02)	0.54	1.07 (1.02 ,1.13)	0.01	0.99 (0.97 ,1.02)	0.65
November	1.06 (1.01 ,1.1)	0.02	0.96 (0.93 ,0.98)	0.000	1.04 (0.99 ,1.09)	0.15	0.97 (0.95 ,1)	0.05	1.04 (0.99 ,1.09)	0.12	0.97 (0.95 ,1)	0.05
December	0.94 (0.9 ,0.99)	0.011	0.82 (0.8 ,0.84)	0.000	0.95 (0.9 ,1.01)	0.08	0.85 (0.83 ,0.88)	0.000	0.96 (0.91 ,1.01)	0.10	0.85 (0.83 ,0.88)	0.000
COVID-19 month												
March	reference				reference				reference		reference	
April	0.95 (0.86 ,1.05)	0.32	0.87 (0.82 ,0.93)	0.000	0.96 (0.85 ,1.08)	0.48	0.88 (0.83 ,0.94)	0.000	0.96 (0.86 ,1.08)	0.49	0.89 (0.83 ,0.95)	0.000
May	0.87 (0.77 ,0.99)	0.03	0.82 (0.76 ,0.88)	0.000	0.85 (0.73 ,0.98)	0.03	0.82 (0.76 ,0.89)	0.000	0.86 (0.74 ,0.99)	0.04	0.83 (0.77 ,0.9)	0.000
June	0.88 (0.79 ,0.99)	0.03	0.84 (0.79 ,0.89)	0.000	0.84 (0.73 ,0.95)	0.01	0.83 (0.78 ,0.89)	0.000	0.84 (0.74 ,0.96)	0.01	0.84 (0.79 ,0.9)	0.000
July	0.95 (0.85 ,1.06)	0.35	0.86 (0.81 ,0.91)	0.000	0.89 (0.79 ,1.01)	0.07	0.87 (0.81 ,0.93)	0.000	0.9 (0.79 ,1.02)	0.10	0.88 (0.83 ,0.95)	0.000
August	0.94 (0.84 ,1.05)	0.25	0.86 (0.81 ,0.92)	0.000	0.95 (0.83 ,1.08)	0.42	0.84 (0.78 ,0.9)	0.000	0.96 (0.84 ,1.1)	0.55	0.85 (0.79 ,0.92)	0.000
September	0.94 (0.83 ,1.06)	0.33	0.97 (0.9 ,1.04)	0.34	0.92 (0.8 ,1.06)	0.27	0.96 (0.89 ,1.03)	0.27	0.94 (0.81 ,1.08)	0.37	0.98 (0.9 ,1.05)	0.55
October	0.97 (0.86 ,1.11)	0.69	0.87 (0.8 ,0.94)	0.000	0.91 (0.78 ,1.05)	0.20	0.87 (0.8 ,0.95)	0.001	0.92 (0.79 ,1.07)	0.26	0.89 (0.82 ,0.96)	0.01
November	0.95 (0.84 ,1.07)	0.42	0.85 (0.79 ,0.92)	0.000	0.88 (0.76 ,1.01)	0.07	0.86 (0.79 ,0.93)	0.000	0.89 (0.77 ,1.03)	0.11	0.87 (0.81 ,0.95)	0.001

© 2023 Glance LG et al. *JAMA Network Open.*

eTable 10. Revascularization with the interaction of race/ethnicity and hospital COVID-19 burden

	Unadjusted				Patient Charactistics				Patient and Hospital Characteristics			
	STEMI		NSTEMI		STEMI		NSTEMI		STEMI		NSTEMI	
	OR (95% CI)	P	OR (95% CI)	P	OR (95% CI)	P	OR (95% CI)	P	OR (95% CI)	P	OR (95% CI)	P
**eTable 10. Revascularization with the interaction of race/ethnicity and hospital COVID-19 burden**												
White	reference				reference				reference		reference	
Black	0.56 (0.53 ,0.58)	0.000	0.57 (0.55 ,0.59)	0.00	0.73 (0.69 ,0.77)	0.000	0.7 (0.67 ,0.73)	0.000	0.76 (0.72 ,0.8)	0.000	0.74 (0.72 ,0.76)	0.000
Hispanic	0.64 (0.58 ,0.71)	0.000	0.65 (0.6 ,0.7)	0.000	0.73 (0.65 ,0.82)	0.000	0.69 (0.63 ,0.76)	0.000	0.8 (0.74 ,0.88)	0.000	0.82 (0.77 ,0.87)	0.000
Age												
65-69	reference				reference				reference		reference	
70-74	0.92 (0.89 ,0.95)	0.000	0.93 (0.92 ,0.94)	0.000	0.9 (0.87 ,0.93)	0.000	0.91 (0.89 ,0.92)	0.000	0.9 (0.87 ,0.93)	0.000	0.91 (0.9 ,0.92)	0.000
75-79	0.72 (0.7 ,0.74)	0.000	0.79 (0.78 ,0.8)	0.000	0.76 (0.74 ,0.78)	0.000	0.81 (0.8 ,0.82)	0.000	0.76 (0.74 ,0.79)	0.000	0.81 (0.8 ,0.82)	0.000
80-84	0.53 (0.51 ,0.55)	0.000	0.6 (0.59 ,0.61)	0.000	0.59 (0.57 ,0.61)	0.000	0.65 (0.64 ,0.66)	0.000	0.59 (0.57 ,0.62)	0.000	0.66 (0.65 ,0.67)	0.000
85-89	0.33 (0.31 ,0.34)	0.000	0.38 (0.37 ,0.39)	0.000	0.38 (0.36 ,0.39)	0.000	0.43 (0.42 ,0.44)	0.000	0.38 (0.37 ,0.4)	0.000	0.44 (0.43 ,0.45)	0.000
90-94	0.17 (0.16 ,0.17)	0.000	0.17 (0.17 ,0.18)	0.000	0.19 (0.18 ,0.2)	0.000	0.2 (0.2 ,0.21)	0.000	0.2 (0.19 ,0.21)	0.000	0.21 (0.2 ,0.22)	0.000
95-	0.07 (0.07 ,0.08)	0.000	0.06 (0.05 ,0.06)	0.000	0.09 (0.08 ,0.09)	0.000	0.07 (0.07 ,0.08)	0.000	0.09 (0.08 ,0.1)	0.000	0.08 (0.07 ,0.08)	0.000
Hospital Covid-19 burden												
Before Covid-19	reference				reference				reference		reference	
0.0-2.0%	0.92 (0.86 ,0.99)	0.02	0.98 (0.95 ,1.01)	0.13	0.94 (0.87 ,1.01)	0.09	0.99 (0.96 ,1.02)	0.36	0.95 (0.89 ,1.02)	0.19	0.99 (0.96 ,1.02)	0.47
2.1-10.0%	0.97 (0.87 ,1.08)	0.56	0.97 (0.92 ,1.03)	0.33	1.03 (0.92 ,1.15)	0.63	1.01 (0.96 ,1.07)	0.70	1 (0.89 ,1.12)	1.00	0.94 (0.89 ,0.99)	0.03
10.1-20.0%	0.89 (0.78 ,1.02)	0.09	0.88 (0.82 ,0.95)	0.001	0.93 (0.81 ,1.08)	0.35	0.95 (0.88 ,1.02)	0.18	0.93 (0.81 ,1.08)	0.36	0.95 (0.88 ,1.02)	0.13
20.1-30.0%	0.9 (0.73 ,1.1)	0.30	0.83 (0.75 ,0.91)	0.000	1 (0.8 ,1.25)	0.99	0.91 (0.82 ,1)	0.05	1.03 (0.82 ,1.3)	0.78	0.94 (0.85 ,1.04)	0.20
30.1%-	0.84 (0.63 ,1.1)	0.21	0.65 (0.56 ,0.76)	0.000	0.86 (0.64 ,1.17)	0.34	0.73 (0.63 ,0.84)	0.000	0.97 (0.72 ,1.3)	0.83	0.84 (0.73 ,0.98)	0.03
COVID Burden X Race												
0.0-2.0% X Black	0.98 (0.84 ,1.14)	0.78	0.94 (0.88 ,1)	0.04	0.99 (0.84 ,1.16)	0.88	0.94 (0.88 ,1)	0.05	0.99 (0.84 ,1.17)	0.93	0.91 (0.86 ,0.97)	0.005
2.1-10% X Black	0.91 (0.77 ,1.09)	0.33	0.92 (0.86 ,0.99)	0.03	0.92 (0.76 ,1.13)	0.43	0.95 (0.88 ,1.02)	0.15	0.92 (0.76 ,1.13)	0.43	0.94 (0.87 ,1.02)	0.12
10.1-20% X Black	0.94 (0.73 ,1.22)	0.65	0.94 (0.84 ,1.05)	0.28	0.98 (0.75 ,1.3)	0.91	0.94 (0.83 ,1.06)	0.32	1.02 (0.76 ,1.35)	0.91	0.95 (0.84 ,1.08)	0.44
20.1-30% X Black	0.97 (0.61 ,1.54)	0.89	1.04 (0.84 ,1.29)	0.74	1.2 (0.75 ,1.94)	0.45	1.04 (0.83 ,1.3)	0.73	1.22 (0.74 ,2.01)	0.43	1.09 (0.87 ,1.37)	0.47
30.1%- X Black	1.14 (0.57 ,2.28)	0.70	1.08 (0.77 ,1.52)	0.67	1.23 (0.58 ,2.6)	0.59	1.09 (0.76 ,1.57)	0.63	1.18 (0.55 ,2.52)	0.67	1.15 (0.78 ,1.69)	0.48
0.0-2.0% X Hispanic	0.93 (0.8 ,1.07)	0.31	0.83 (0.77 ,0.9)	0.00	0.92 (0.79 ,1.08)	0.31	0.82 (0.76 ,0.89)	0.000	0.91 (0.78 ,1.06)	0.22	0.84 (0.78 ,0.91)	0.000
2.1-10% X Hispanic	1.15 (0.97 ,1.37)	0.11	1.05 (0.97 ,1.14)	0.25	1.27 (1.04 ,1.54)	0.02	1.11 (1.01 ,1.22)	0.03	1.24 (1.02 ,1.5)	0.03	1.07 (0.98 ,1.18)	0.14
10.1-20% X Hispanic	1.24 (0.93 ,1.65)	0.14	1.08 (0.93 ,1.24)	0.32	1.26 (0.91 ,1.74)	0.17	1.11 (0.95 ,1.29)	0.19	1.25 (0.9 ,1.72)	0.18	1.1 (0.95 ,1.28)	0.22
20.1-30% X Hispanic	1.52 (0.92 ,2.51)	0.10	0.99 (0.79 ,1.24)	0.93	1.71 (0.96 ,3.06)	0.07	1.05 (0.83 ,1.33)	0.71	1.56 (0.86 ,2.83)	0.15	1.1 (0.85 ,1.41)	0.48
30.1%- X Hispanic	0.94 (0.58 ,1.53)	0.80	0.84 (0.63 ,1.14)	0.26	1.23 (0.71 ,2.11)	0.46	0.96 (0.71 ,1.3)	0.79	1.13 (0.66 ,1.94)	0.65	0.87 (0.63 ,1.19)	0.38
Sex												
Male					reference				reference		reference	
Female					0.72 (0.71 ,0.74)	0.000	0.67 (0.66 ,0.67)	0.000	0.72 (0.71 ,0.74)	0.000	0.67 (0.66 ,0.67)	0.000
Urgency												
Urgent					0.92 (0.87 ,0.97)	0.004	1.09 (1.03 ,1.16)	0.005	0.91 (0.86 ,0.96)	0.001	1.1 (1.03 ,1.16)	0.002
Emergent					reference				reference		reference	

© 2023 Glance LG et al. *JAMA Network Open.*

	Unadjusted				Patient Charactistics				Patient and Hospital Characteristics			
	STEMI		NSTEMI		STEMI		NSTEMI		STEMI		NSTEMI	
	OR (95% CI)	P	OR (95% CI)	P	OR (95% CI)	P	OR (95% CI)	P	OR (95% CI)	P	OR (95% CI)	P
Admission source												
Community					reference				reference		reference	
Hospital					0.91 (0.86 ,0.97)	0.004	1.67 (1.58 ,1.75)	0.000	0.84 (0.8 ,0.89)	0.000	1.33 (1.27 ,1.4)	0.000
SNF/Nursing Home					0.39 (0.35 ,0.43)	0.000	0.45 (0.42 ,0.48)	0.000	0.39 (0.35 ,0.43)	0.000	0.45 (0.42 ,0.48)	0.000
Other					0.9 (0.81 ,1)	0.05	1.32 (1.23 ,1.41)	0.000	0.87 (0.78 ,0.97)	0.01	1.16 (1.08 ,1.25)	0.000
Dual-eligible					0.77 (0.74 ,0.8)	0.000	0.77 (0.75 ,0.79)	0.000	0.78 (0.76 ,0.81)	0.000	0.8 (0.78 ,0.81)	0.000
Body Mass Index												
Underweight					0.59 (0.54 ,0.65)	0.000	0.63 (0.6 ,0.67)	0.000	0.58 (0.53 ,0.64)	0.00	0.61 (0.58 ,0.65)	0.000
Morbid obesity					1.01 (0.95 ,1.08)	0.693	1.03 (1 ,1.05)	0.046	1.01 (0.94 ,1.08)	0.78	1.02 (0.99 ,1.04)	0.12
Myocardial infarction location												
Inferior wall					reference		NA		reference		NA	
Anterior wall					0.99 (0.96 ,1.01)	0.31	NA		0.99 (0.97 ,1.02)	0.67	NA	
Lateral wall					0.69 (0.65 ,0.73)	0.000	NA		0.7 (0.66 ,0.74)	0.000	NA	
Unspecified					0.24 (0.23 ,0.25)	0.000	NA		0.25 (0.24 ,0.26)	0.000	NA	
Congestive heart failure												
Systolic					0.86 (0.83 ,0.89)	0.000	0.8 (0.79 ,0.82)	0.000	0.86 (0.83 ,0.89)	0.000	0.78 (0.76 ,0.79)	0.000
Diastolic					0.84 (0.8 ,0.88)	0.000	0.76 (0.74 ,0.78)	0.000	0.84 (0.8 ,0.88)	0.000	0.75 (0.73 ,0.76)	0.000
Systolic & Diastolic					0.82 (0.78 ,0.87)	0.000	0.81 (0.79 ,0.83)	0.000	0.82 (0.78 ,0.87)	0.000	0.79 (0.77 ,0.82)	0.000
Unspecified					0.63 (0.6 ,0.66)	0.000	0.66 (0.64 ,0.68)	0.000	0.65 (0.62 ,0.68)	0.000	0.69 (0.67 ,0.71)	0.000
Right heart failure					0.83 (0.68 ,1.02)	0.08	0.84 (0.74 ,0.96)	0.000	0.83 (0.68 ,1.03)	0.088	0.84 (0.74 ,0.96)	0.01
Biventricular heart failure					0.73 (0.59 ,0.9)	0.004	0.85 (0.75 ,0.96)	0.008	0.71 (0.57 ,0.88)	0.002	0.8 (0.71 ,0.91)	0.001
End-stage heart failure					0.54 (0.37 ,0.78)	0.001	0.65 (0.55 ,0.76)	0.011	0.54 (0.37 ,0.78)	0.001	0.63 (0.54 ,0.74)	0.000
Complications of AMI												
Ventricular septal defect					0.6 (0.5 ,0.7)	0.000	1 (0.6 ,1.5)	0.84	0.6 (0.5 ,0.7)	0.000	0.9 (0.5 ,1.4)	0.56
LV rupture					0.5 (0.4 ,0.6)	0.000	1.1 (0.7 ,1.7)	0.64	0.5 (0.3 ,0.6)	0.000	1.1 (0.7 ,1.8)	0.72
Papillary muscle rupture					0.9 (0.5 ,1.4)	0.56	1.8 (1.2 ,2.8)	0.005	0.8 (0.5 ,1.3)	0.48	1.7 (1.1 ,2.7)	0.02
Prior procedures												
PCI					0.92 (0.89 ,0.96)	0.000	1.01 (0.99 ,1.03)	0.50	0.92 (0.89 ,0.96)	0.000	0.99 (0.97 ,1)	0.14
CABG					0.45 (0.43 ,0.47)	0.000	0.6 (0.59 ,0.61)	0.000	0.44 (0.42 ,0.46)	0.000	0.58 (0.57 ,0.59)	0.000
Heart valve surgery					0.66 (0.6 ,0.72)	0.000	0.72 (0.7 ,0.75)	0.000	0.66 (0.6 ,0.72)	0.000	0.7 (0.68 ,0.73)	0.000
Dialysis					0.55 (0.51 ,0.6)	0.000	0.86 (0.83 ,0.88)	0.000	0.55 (0.51 ,0.6)	0.000	0.86 (0.83 ,0.88)	0.000
AICD					0.53 (0.48 ,0.58)	0.000	0.68 (0.66 ,0.7)	0.000	0.53 (0.48 ,0.58)	0.000	0.67 (0.65 ,0.69)	0.000
COVID					0.55 (0.44 ,0.7)	0.000	0.43 (0.37 ,0.49)	0.000	0.55 (0.43 ,0.69)	0.000	0.42 (0.36 ,0.48)	0.000
Functional Status												
Wheel-chair					0.51 (0.43 ,0.6)	0.000	0.59 (0.55 ,0.64)	0.000	0.51 (0.43 ,0.6)	0.000	0.57 (0.53 ,0.62)	0.000
Supplemental oxygen					0.67 (0.62 ,0.72)	0.000	0.7 (0.68 ,0.72)	0.000	0.68 (0.63 ,0.73)	0.000	0.71 (0.69 ,0.73)	0.000
Dependent on provider					0.32 (0.26 ,0.38)	0.000	0.35 (0.31 ,0.4)	0.000	0.33 (0.27 ,0.39)	0.000	0.36 (0.32 ,0.41)	0.000
Elixhauser comorbidities												
Cardiac arrhythmias					0.88 (0.86 ,0.9)	0.000	0.8 (0.79 ,0.81)	0.000	0.87 (0.85 ,0.89)	0.000	0.78 (0.77 ,0.79)	0.000
Valvular heart disease					0.89 (0.86 ,0.92)	0.000	0.98 (0.97 ,1)	0.04	0.87 (0.84 ,0.9)	0.000	0.94 (0.93 ,0.96)	0.000
Pulmonary circulation					0.77 (0.73 ,0.81)	0.000	0.84 (0.82 ,0.86)	0.000	0.76 (0.73 ,0.8)	0.000	0.83 (0.81 ,0.85)	0.000
Peripheral vascular disorder					0.85 (0.81 ,0.88)	0.000	1.05 (1.03 ,1.08)	0.000	0.83 (0.8 ,0.87)	0.000	1.03 (1 ,1.05)	0.03

© 2023 Glance LG et al. *JAMA Network Open.*

	Unadjusted				Patient Charactistics				Patient and Hospital Characteristics			
	STEMI		NSTEMI		STEMI		NSTEMI		STEMI		NSTEMI	
	OR (95% CI)	P	OR (95% CI)	P	OR (95% CI)	P	OR (95% CI)	P	OR (95% CI)	P	OR (95% CI)	P
Hypertension, uncomplicated					1.11 (1.08 ,1.14)	0.000	1.18 (1.16 ,1.2)	0.000	1.1 (1.06 ,1.13)	0.000	1.16 (1.14 ,1.18)	0.000
Hypertension, complicated					1.1 (1.06 ,1.14)	0.000	1.2 (1.17 ,1.23)	0.000	1.08 (1.04 ,1.12)	0.000	1.17 (1.14 ,1.19)	0.000
Paralysis					0.65 (0.56 ,0.74)	0.000	0.77 (0.72 ,0.83)	0.000	0.64 (0.56 ,0.74)	0.000	0.75 (0.7 ,0.81)	0.000
Neurologic disorder, other					0.48 (0.47 ,0.5)	0.000	0.58 (0.56 ,0.59)	0.000	0.48 (0.46 ,0.5)	0.000	0.57 (0.56 ,0.58)	0.000
Chronic pulmonary disease					0.86 (0.84 ,0.88)	0.000	0.84 (0.83 ,0.85)	0.000	0.86 (0.84 ,0.89)	0.000	0.84 (0.83 ,0.85)	0.000
Diabetes, uncomplicated					0.9 (0.88 ,0.93)	0.000	0.98 (0.97 ,1)	0.01	0.91 (0.88 ,0.94)	0.000	1 (0.98 ,1.01)	0.83
Diabetes, complicated					0.93 (0.9 ,0.96)	0.000	1.08 (1.07 ,1.1)	0.000	0.92 (0.89 ,0.95)	0.000	1.07 (1.06 ,1.09)	0.000
Hypothyroidism					0.99 (0.96 ,1.02)	0.33	0.99 (0.97 ,1)	0.03	0.98 (0.95 ,1.01)	0.22	0.98 (0.97 ,0.99)	0.001
Renal failure					0.84 (0.81 ,0.87)	0.00	0.81 (0.8 ,0.83)	0.000	0.85 (0.82 ,0.88)	0.000	0.81 (0.79 ,0.82)	0.000
Liver disease					0.72 (0.69 ,0.77)	0.00	0.75 (0.72 ,0.77)	0.000	0.72 (0.68 ,0.76)	0.000	0.73 (0.7 ,0.75)	0.000
Peptic ulcer disease					1.02 (0.87 ,1.18)	0.83	0.85 (0.81 ,0.9)	0.000	1.02 (0.87 ,1.19)	0.80	0.83 (0.79 ,0.87)	0.000
AIDS/HIV					1.2 (0.79 ,1.83)	0.40	1.01 (0.85 ,1.2)	0.93	1.24 (0.8 ,1.91)	0.33	0.98 (0.82 ,1.16)	0.80
Lymphoma					0.76 (0.67 ,0.87)	0.000	0.71 (0.67 ,0.74)	0.000	0.75 (0.66 ,0.86)	0.000	0.68 (0.65 ,0.72)	0.000
Metastatic cancer					0.42 (0.39 ,0.46)	0.000	0.49 (0.47 ,0.51)	0.000	0.42 (0.38 ,0.46)	0.000	0.48 (0.46 ,0.5)	0.000
Solid tumor					0.65 (0.61 ,0.69)	0.000	0.69 (0.67 ,0.71)	0.000	0.65 (0.61 ,0.69)	0.000	0.68 (0.66 ,0.7)	0.000
Rheumatoid arthritis					1.06 (1 ,1.13)	0.05	1 (0.98 ,1.02)	0.95	1.06 (1 ,1.13)	0.049	0.98 (0.95 ,1)	0.07
Coagulopathy					0.88 (0.84 ,0.93)	0.000	1.01 (0.98 ,1.03)	0.53	0.87 (0.83 ,0.92)	0.000	0.98 (0.96 ,1.01)	0.20
Weight loss					0.68 (0.64 ,0.73)	0.000	0.71 (0.68 ,0.74)	0.000	0.69 (0.64 ,0.74)	0.000	0.71 (0.69 ,0.74)	0.000
Fluid and electrolyte disorder					0.64 (0.62 ,0.65)	0.000	0.7 (0.69 ,0.71)	0.000	0.64 (0.62 ,0.66)	0.000	0.7 (0.69 ,0.71)	0.000
Anemia, blood loss					0.77 (0.67 ,0.88)	0.000	0.76 (0.72 ,0.81)	0.000	0.79 (0.69 ,0.91)	0.001	0.76 (0.72 ,0.81)	0.000
Anemia, deficiency					0.89 (0.83 ,0.95)	0.000	0.85 (0.83 ,0.88)	0.000	0.89 (0.83 ,0.95)	0.001	0.84 (0.82 ,0.87)	0.000
Alcohol abuse					0.8 (0.74 ,0.86)	0.000	0.8 (0.78 ,0.83)	0.000	0.79 (0.73 ,0.86)	0.000	0.81 (0.78 ,0.83)	0.000
Drug abuse					0.91 (0.83 ,1)	0.06	0.8 (0.76 ,0.83)	0.000	0.91 (0.83 ,1)	0.06	0.8 (0.76 ,0.83)	0.000
Psychoses					0.47 (0.41 ,0.53)	0.000	0.48 (0.44 ,0.51)	0.000	0.48 (0.41 ,0.55)	0.000	0.49 (0.45 ,0.52)	0.000
Depression					0.84 (0.81 ,0.87)	0.000	0.9 (0.88 ,0.92)	0.000	0.83 (0.8 ,0.87)	0.000	0.88 (0.87 ,0.9)	0.000
Volume of AMI hospitalizations												
<200									reference		reference	
200-499									2.52 (2.23 ,2.84)	0.000	3.14 (2.75 ,3.59)	0.000
500-999									3.07 (2.73 ,3.44)	0.000	4.63 (4.09 ,5.26)	0.000
1000-1999									3.18 (2.83 ,3.59)	0.000	5.37 (4.72 ,6.11)	0.000
2000-									2.96 (2.52 ,3.47)	0.000	5.43 (4.66 ,6.34)	0.000
Proportion of minority patients (AMI)												
<5%									reference		reference	
5.0-9.9%									1 (0.93 ,1.07)	0.93	0.98 (0.9 ,1.05)	0.52
10.0-24.9									1 (0.93 ,1.07)	0.98	1.05 (0.98 ,1.13)	0.20
25.0-49.9%									0.96 (0.88 ,1.05)	0.35	0.98 (0.89 ,1.06)	0.57
50.0%-									0.7 (0.58 ,0.83)	0.000	0.65 (0.55 ,0.76)	0.000

© 2023 Glance LG et al. *JAMA Network Open.*

	Unadjusted				Patient Charactistics				Patient and Hospital Characteristics			
	STEMI		NSTEMI		STEMI		NSTEMI		STEMI		NSTEMI	
	OR (95% CI)	P	OR (95% CI)	P	OR (95% CI)	P	OR (95% CI)	P	OR (95% CI)	P	OR (95% CI)	P
Disproportionate share percentage (DSH)												
0-9.9%									reference		reference	
10.0-24.9%									1.01 (0.91 ,1.12)	0.88	1 (0.88 ,1.13)	0.96
25.0-49.9%									1.05 (0.94 ,1.17)	0.39	1.01 (0.89 ,1.16)	0.84
50.0%-									1.32 (1.12 ,1.54)	0.00	1.17 (0.99 ,1.4)	0.07
Resident-to-bed ratio												
0									reference		reference	
>0-0.10									0.97 (0.91 ,1.04)	0.41	1.05 (0.98 ,1.12)	0.14
0.11-0.20									1.05 (0.97 ,1.13)	0.25	1.09 (1 ,1.18)	0.04
0.21-0.40									0.95 (0.87 ,1.04)	0.29	1.04 (0.95 ,1.15)	0.38
0.41-									0.94 (0.85 ,1.03)	0.19	1.2 (1.1 ,1.32)	0.000
Rurality												
Rural hospital									reference		reference	
Large urban hospital									1.1 (1 ,1.22)	0.046	1.16 (1.04 ,1.29)	0.008
Other urban hospital									1.1 (1 ,1.21)	0.048	1.21 (1.09 ,1.34)	0.000
Time	1.01 (1.01 ,1.01)	0	1.01 (1.01 ,1.01)	0.000	1.01 (1.01 ,1.01)	0.000	1.01 (1.01 ,1.01)	0.000	1.01 (1.01 ,1.01)	0.000	1.01 (1 ,1.01)	0.000
Admission month												
January	reference				reference				reference		reference	
February	0.99 (0.94 ,1.03)	0.56	1 (0.98 ,1.02)	0.77	1.01 (0.96 ,1.06)	0.83	1 (0.98 ,1.02)	0.98	1.01 (0.96 ,1.06)	0.77	1 (0.98 ,1.02)	0.69
March	0.99 (0.95 ,1.04)	0.73	1.02 (1 ,1.05)	0.03	1.01 (0.96 ,1.07)	0.67	1.02 (1 ,1.04)	0.08	1.01 (0.96 ,1.07)	0.59	1.01 (0.99 ,1.03)	0.31
April	1.01 (0.96 ,1.06)	0.79	1.04 (1.01 ,1.06)	0.001	1.01 (0.96 ,1.06)	0.78	1.02 (1 ,1.05)	0.03	1.01 (0.96 ,1.07)	0.69	1.02 (0.99 ,1.04)	0.14
May	0.96 (0.91 ,1.01)	0.09	1.04 (1.02 ,1.07)	0.000	0.97 (0.92 ,1.02)	0.26	1.03 (1.01 ,1.05)	0.005	0.97 (0.92 ,1.02)	0.27	1.02 (1 ,1.04)	0.06
June	0.96 (0.91 ,1.01)	0.09	1.02 (1 ,1.05)	0.025	0.97 (0.92 ,1.02)	0.23	1.01 (0.99 ,1.04)	0.24	0.97 (0.92 ,1.02)	0.29	1 (0.98 ,1.02)	0.98
July	0.96 (0.91 ,1)	0.08	1.01 (0.99 ,1.03)	0.418	0.97 (0.92 ,1.02)	0.30	0.99 (0.97 ,1.02)	0.63	0.98 (0.93 ,1.03)	0.36	0.98 (0.96 ,1.01)	0.16
August	0.94 (0.9 ,0.99)	0.02	1.01 (0.99 ,1.03)	0.396	0.96 (0.91 ,1.01)	0.12	1 (0.98 ,1.02)	0.99	0.96 (0.91 ,1.01)	0.15	0.99 (0.97 ,1.01)	0.44
September	0.9 (0.86 ,0.95)	0.00	0.98 (0.96 ,1)	0.037	0.92 (0.87 ,0.97)	0.00	0.97 (0.95 ,0.99)	0.006	0.92 (0.87 ,0.97)	0.00	0.96 (0.94 ,0.98)	0.000
October	0.96 (0.91 ,1)	0.07	1 (0.98 ,1.02)	0.92	0.96 (0.91 ,1.01)	0.15	0.99 (0.97 ,1.01)	0.38	0.96 (0.91 ,1.02)	0.19	0.99 (0.96 ,1.01)	0.25
November	0.92 (0.88 ,0.97)	0.00	0.99 (0.96 ,1.01)	0.17	0.94 (0.89 ,0.99)	0.02	0.98 (0.96 ,1)	0.051	0.95 (0.9 ,1)	0.04	0.97 (0.95 ,1)	0.02
December	0.91 (0.86 ,0.95)	0.000	0.9 (0.88 ,0.92)	0.00	0.9 (0.85 ,0.95)	0.000	0.87 (0.85 ,0.89)	0.000	0.91 (0.86 ,0.96)	0.000	0.87 (0.85 ,0.89)	0.000
COVID-19 month												
March	reference				reference				reference		reference	
April	0.98 (0.88 ,1.09)	0.67	0.98 (0.94 ,1.03)	0.495	0.94 (0.83 ,1.05)	0.28	0.96 (0.91 ,1.01)	0.12	0.94 (0.83 ,1.05)	0.27	0.97 (0.92 ,1.02)	0.27
May	0.97 (0.85 ,1.1)	0.59	1.11 (1.04 ,1.18)	0.001	0.92 (0.8 ,1.05)	0.22	1.08 (1.01 ,1.15)	0.03	0.93 (0.81 ,1.07)	0.31	1.11 (1.04 ,1.18)	0.00
June	0.99 (0.88 ,1.12)	0.87	1.05 (1 ,1.11)	0.063	1 (0.87 ,1.13)	0.95	1.04 (0.98 ,1.1)	0.22	1.02 (0.89 ,1.16)	0.81	1.07 (1.02 ,1.14)	0.01
July	1 (0.89 ,1.12)	0.98	1.04 (0.99 ,1.1)	0.14	1 (0.89 ,1.14)	0.95	1.02 (0.97 ,1.08)	0.46	1.02 (0.9 ,1.16)	0.72	1.07 (1.01 ,1.13)	0.01
August	0.99 (0.87 ,1.12)	0.85	1.01 (0.95 ,1.08)	0.69	0.97 (0.85 ,1.12)	0.71	1 (0.94 ,1.07)	0.95	0.99 (0.86 ,1.14)	0.92	1.04 (0.98 ,1.11)	0.22
September	0.99 (0.87 ,1.12)	0.84	1.01 (0.95 ,1.08)	0.73	0.98 (0.85 ,1.13)	0.79	0.99 (0.93 ,1.06)	0.86	1.01 (0.88 ,1.16)	0.91	1.04 (0.97 ,1.1)	0.28
October	0.97 (0.84 ,1.12)	0.69	0.98 (0.92 ,1.05)	0.54	0.98 (0.84 ,1.15)	0.81	0.96 (0.89 ,1.02)	0.20	1 (0.86 ,1.17)	0.98	1 (0.93 ,1.07)	0.95
November	0.94 (0.82 ,1.09)	0.43	0.99 (0.93 ,1.06)	0.84	0.94 (0.81 ,1.1)	0.44	0.96 (0.9 ,1.03)	0.30	0.96 (0.83 ,1.12)	0.63	1 (0.94 ,1.07)	0.92

© 2023 Glance LG et al. *JAMA Network Open.*
