# Supplementary material for: The COVID-19 Pandemic and Associated Inequities in Acute Myocardial Infarction Treatment and Outcomes
Source: JAMA Netw Open. 2023 Aug 25;6(8):e2330327. doi: 10.1001/jamanetworkopen.2023.30327 (PMC10457721; doi:10.1001/jamanetworkopen.2023.30327)
Supplement: Supplement 2. — Data Sharing Statement [file jamanetwopen-e2330327-s002.pdf]

Data Sharing Statement

Glance. The COVID-19 Pandemic and Associated Inequities in Acute Myocardial Infarction Treatment and Outcomes. *JAMA Netw Open*. Published online August 25, 2023. doi:10.1001/jamanetworkopen.2023.30327

Data
Data available: No
